# LAG-1: A dynamic, integrative model of learning, attention, and gaze

**DOI:** 10.1371/journal.pone.0259511

**Published:** 2022-03-17

**Authors:** Jordan Barnes, Mark R. Blair, R. Calen Walshe, Paul F. Tupper

**Affiliations:** 1 Department of Psychology, Simon Fraser University, Burnaby, BC, Canada; 2 Center for Perceptual Systems, University of Texas, Austin, Texas, United States of America; 3 Department of Mathematics, Simon Fraser University, Burnaby, BC, Canada; The University of Queensland, AUSTRALIA

## Abstract

It is clear that learning and attention interact, but it is an ongoing challenge to integrate their psychological and neurophysiological descriptions. Here we introduce LAG-1, a dynamic neural field model of learning, attention and gaze, that we fit to human learning and eye-movement data from two category learning experiments. LAG-1 comprises three control systems: one for visuospatial attention, one for saccadic timing and control, and one for category learning. The model is able to extract a kind of information gain from pairwise differences in simple associations between visual features and categories. Providing this gain as a reentrant signal with bottom-up visual information, and in top-down spatial priority, appropriately influences the initiation of saccades. LAG-1 provides a moment-by-moment simulation of the interactions of learning and gaze, and thus simultaneously produces phenomena on many timescales, from the duration of saccades and gaze fixations, to the response times for trials, to the slow optimization of attention toward task relevant information across a whole experiment. With only three free parameters (learning rate, trial impatience, and fixation impatience) LAG-1 produces qualitatively correct fits for learning, behavioural timing and eye movement measures, and also for previously unmodelled empirical phenomena (e.g., fixation orders showing stimulus-specific attention, and decreasing fixation counts during feedback). Because LAG-1 is built to capture attention and gaze generally, we demonstrate how it can be applied to other phenomena of visual cognition such as the free viewing of visual stimuli, visual search, and covert attention.

## Introduction

An important part of learning is learning what’s important. Experts can be distinguished from novices based on the sources of information they use to make classifications. For example, expert bird watchers use subtle but predictive features of birdsong to distinguish between different bird species, identify birds using their specific names, and describe birds using behavioural traits, whereas novices do not [1, 2]. Analogous findings about the features used by experts and novices to make classifications have been found in biology [3, 4], physics [5], and computer programming [6], among many other areas. Knowledge about what’s important guides where people look. In reading, for example, many indicators of a new, or struggling, reader are obvious in their eye movements: excess fixations, fixations to previous words, or to determiners like “the” [7]. Similarly, novice drivers tend to look more at the road ahead and underutilize other important sources of information, relative to more experienced drivers [8]. Laboratory studies looking at eye movements during learning tend to show that the overall number of gaze fixations, the fixations to irrelevant information, and the durations of fixations decrease as skills are increased [9–11]. Learning influences the allocation of gaze and attention, but the reverse is also true. Providing novices with attentional instruction can speedily close their performance gap with experts; for example, in a classic study of chicken sexing—a notoriously difficult perceptual challenge—researchers were able to markedly improve novice performance by training them how to prioritize the most informative parts [12].

One paradigm that is excellent for eliciting the interactions of learning and attention is category learning [13–18]. Experiments with this paradigm are simple enough to be modelled, yet constitute a full sweep through the cognitive system, including: perception, attention, memory, and decision making, all packed into hundreds of incremental learning trials occurring over cognitively distinguished time scales [19, 20]. In a typical category learning study, the stimulus that is presented to the participant is a category exemplar composed of 3–4 visual features. Participants then choose one of the possible categories (usually between 2–4 categories). At first, participants are just guessing: they do not yet know which features are indicative of which categories. Each successive trial presents a new stimulus, solicits a response, and typically provides corrective feedback, enabling participants, over hundreds of trials, to learn how to categorize accurately. Usually, some of the features are more relevant than others for predicting the correct category, leading participants to eventually selectively attend to those features.

Category learning researchers have measured attention using both indirect methods—like with knowledge transfer tasks, wherein a research participant is required to categorize unseen exemplars after experience with a training set, and thus reveal what they deemed important (e.g. [21])—and direct methods, that require participants to explicitly reveal stimulus information using mouse movements or clicks for example [22–25]. Probably the most natural way of capturing the deployment of attention during category learning, however, is using eye tracking. Many general eye tracking findings have now been documented (see [26] for a summary of measures across 10 different experiments). Perhaps the simplest and most common finding is that the number of eye movements to stimulus features in a categorization trial starts high and drops over the course of an experiment. In most cases, it settles to just above the number of features necessary to consistently generate a correct classification of the stimulus [10, 27, 28]. The duration of individual fixations to features has also been shown to decrease as participants learn [29]. Participants spend more of their time looking at relevant features than irrelevant ones [10, 11], and they fixate important features earlier in a trial [10, 11, 25, 29]. Finally, the amount of time that participants spend viewing features during feedback drops as the participants learn how to more efficiently attend in the task [30]. In all of these findings, behavioural measures of attention are strongly related to behavioral measures of learning.

The full set of findings relating to learning and attention in category learning is challengingly diverse. Participants are choosing what features to look at and for how long, and in what order. They are deciding when they know enough to choose a category and which category is correct. They are then choosing if, when, and how long to look at the feedback and represented stimulus features, and when to move on to the next trial. Participant’s sampling of information is continuous, and intertwined with the learning process itself. Further, behaviour adapts at a wide variety of timescales: saccades operate on the order of 100 ms, fixations about 200–400 ms, task length fixation ordering 1000–5000 ms, and learning-related changes reveal themselves over whole experiments lasting 180,000 ms. We know of no existing model which links learning with attention and gaze that can make quantitative predictions about the full set of findings relating learning, and gaze in these tasks. This is in no small part because no one has tried. Instead, researchers have chosen to carve off smaller portions of the problem, trading generality for tractability. Some examples of findings and their corresponding models can be found in Table 1. To us, these phenomena seem to naturally arise from the interaction of a simple learning mechanism, a simple attentional priority map, and a simple saccade timing system.

**Table 1:** Eye tracking, timing and category learning findings.

Finding	Empirical source	Model
Reaction time reduction	Homa and Fish (1975), McColeman et al. (2014) [26, 31].	Lamberts (1998), Logan (2002), Nosofsky and Palmeri (1997) [32–34].
Fixation count reduction	McColeman et al. (2014), Rehder and Hoffman (2005) [10, 26].	Barnes, McColeman, Blair, and Walshe (2014), Nelson and Cottrell (2007) [28, 35].
Fixation duration reduction	Blair, Watson, Walshe, and Maj (2009), McColeman et al. (2014) [11, 26].	N/A
Fixation ordering	Blair, Watson, Walshe, and Maj (2009), Chen, Meier, Blair, Watson, and Wood (2013), Rehder and Hoffman (2005) [10, 11, 29].	Rombouts, Bohte, Martinez-Trujillo, and Roelfsema (2015) [36].
Reduced feedback use	Bourne, Guy, Dodd, and Justesen (1965), Watson and Blair (2008) [30, 37].	N/A

N/A indicates findings that are modelled for the first time here.

## LAG-1

In the present paper, we introduce LAG-1, a computational model of learning, attention and gaze designed to process common experimental manipulations in category learning tasks. The name emphasizes the three cognitive components we are attempting to combine. It also emphasizes the temporal nature of the integration of these processes: learning, attention and gaze, moment by moment. In this section, we describe the model in detail. We start with a high level description of its core theoretical commitments, and then explain how its structure relates to both the theoretical claim and to its behavioural predictions by showing how activation flows through the various components of the model. Finally, for those looking for exact implementational details and correspondences with neurophysiology, we provide some description of the neurophysiological and behavioural studies relevant to each component of the model and provide the equations for each component in the supplementary information.

At the heart of the model is a theoretical idea about how learning, attention, and gaze interact: simple associative learning affords an additional source of information about the importance of features beyond just the base-rate driven weights: later in learning, the contrast of these weights also tells you how diagnostic the features are. Measures of expected information gain are commonly employed to model visual attention (e.g., [38]), where this sort of quantity is integrated with bottom-up visual signals in an attentional priority map (e.g., [39]), that strongly influences saccade initiation [40]. According to LAG-1, these linkages between learning and attention, and between attention and saccadic initiation are the primary cause of the documented covariation between learning and gaze seen in the myriad measures of participant behaviour in the category learning paradigm. Thus, implementing these ideas in a computational framework should allow us to predict, at least qualitatively, the changes to gaze that correspond with the changes in participant performance.

To test LAG-1, we simulate two category learning experiments that also recorded eye gaze [10, 25]. The empirical findings relevant to these simulations are listed in Table 1. Because the central phenomenon of interest to LAG-1 is the interrelationship between attention and learning, we report learning performance, via category responses and stimulus generalization. There are also a number of generic timing measures that can be fit, such as how long participants spend before making a categorization response, or how long they spend viewing feedback before continuing on to the next trial.

Implementing a model that is continuous in space and time and also neurally plausible—one of our chief design considerations—requires a suitable modelling framework. We implement LAG-1 using ideas from Dynamic Neural Field Theory (DNFT). This framework has an extensive track record of model-based explanations in developmental [41–44] and cognitive psychology [45–47]. Neural fields are well-suited to modelling the brain’s numerous topographic maps, like those implicated in the making of eye movements [48, 49]. For example, the characteristic properties of dynamic neural fields have been used to explain the critical distances inherent to averaging saccades by modelling the functional relationships of the superior colliculus [46]. DNFT models have also provided important insights into the temporal dynamics of cognitive processes like object recognition, by naturally reflecting the time needed to resolve competition proportionate to the multidimensional metric confusability of object features (the “Lyupanov time” [50]), e.g., distinguishing a lime from a lemon takes longer than distinguishing a lime from banana) [51]. We note that, while the logic of modelling with DNFT is compelling, there are alternatives, such as simplifying the lateral influences of spatial configuration using discrete dynamical units that still preserve the temporal dynamics of interest, e.g., [52]. By building LAG-1 using DNFT however, the underlying structure of the model naturally accommodates any temporal and spatial aspects of cognitive processes in ways that match the computational properties of real neural systems.

We designed LAG-1 primarily to process visual category learning experiments, but very few changes were needed in order to have it process another important class of attention tasks: visual search. The benchmark measures for visual search include very different kinds of phenomena than in category learning, such as the distractor-heterogeneity effect, and the feature-target similarity effect, as well as several other findings which have been well documented elsewhere, e.g., [53]. It is noteworthy that temporal models of visual search are accounting for benchmark phenomena by allowing knowledge of the critical features of a target to amplify those particular feature dimensions as search items compete with one another for attention [54, 55]: attention weights have a long history in category learning models, and this suggests that progress in one domain may naturally complement the other.

As there are by now many dynamical cognitive process models of attention and learning, it is encouraging to see models try to build on previous efforts, especially when it comes to identifying neural processes that collectively act as a functional subsystem untethered from a specific model. For example, [56] builds on the influential category learning model “COmpetition between Verbal and Implicit Systems” (COVIS) [57], by replacing the original visual processing methods of the model with more precise models of cortical and subcortical structures at each level of the visual processing hierarchy. By exploiting the modularity of these visual processes the extended model increased the category learning phenomena that could in principle be accounted for, such as: the effect of feedback delays, or dimensional relevance shifts. LAG-1 similarly aims to be “plug-n-play”, at the level of neurophysiologically plausible subsystems, where for example, the comparatively simple associative learning subsystem in LAG-1 could be swapped out or extended by a more complex feedback-driven learning system capable of learning non-linear category structures.

LAG-1 is thus part of a wider group of dynamic models of cognition that are taking on the challenge of capturing the temporal nature of cognitive processes by directly modelling neural processes at the level of sensorimotor interactions between learning and attention.

**Fig 1:**
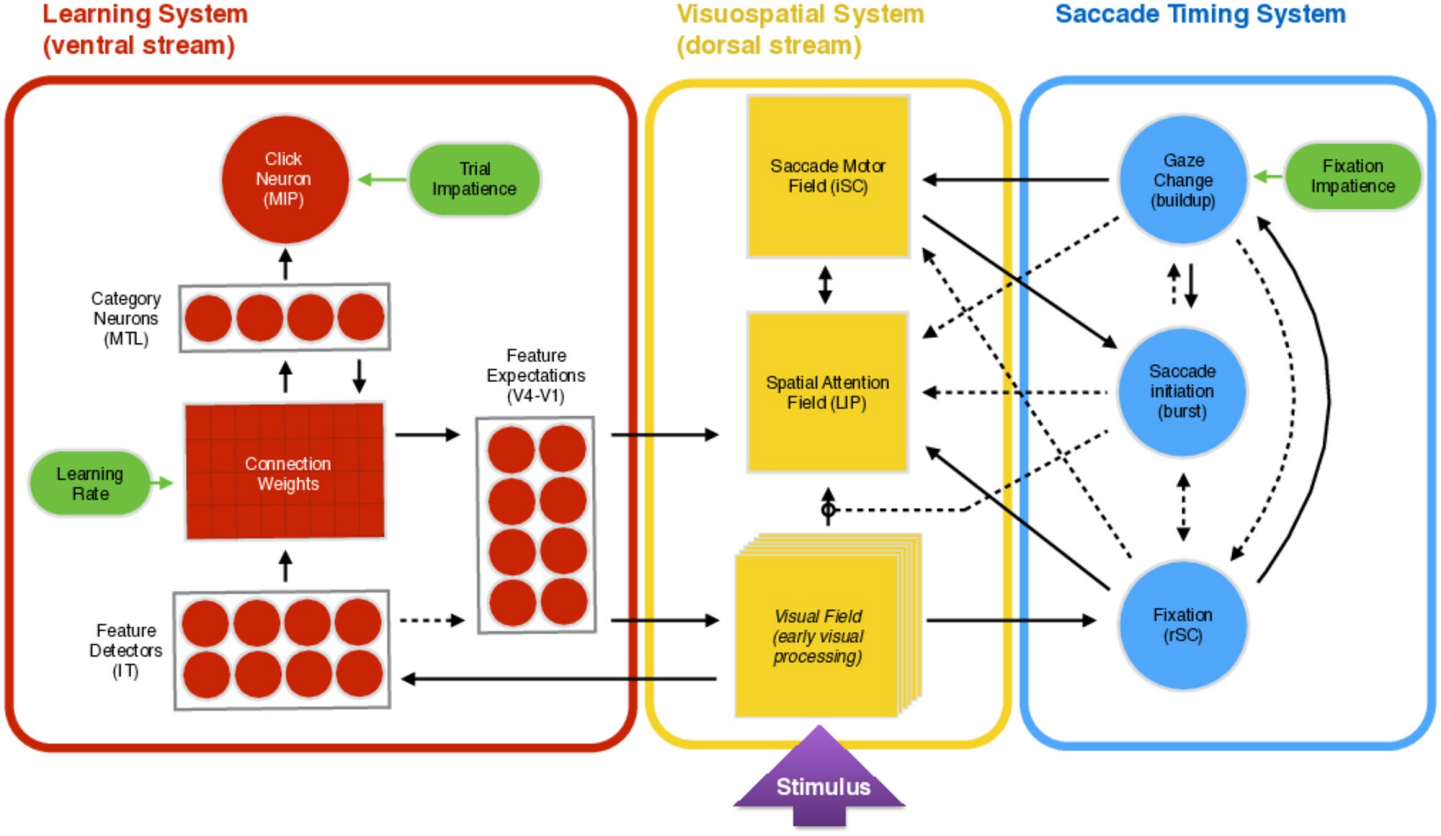
Model structure. The major components of LAG-1 and the excitatory (solid) and inhibitory (dashed) connections between them. There are three functional subsystems: Visuospatial Processing, Learning, and Saccade Timing. Labels in brackets refer to neuroanatomical regions that have similar functions. Green ovals represent the free parameters we varied to fit individual subjects. Circles represent single model neurons, rectangles denote fields, and circles within rectangles specify single neurons with some level of lateral interaction.

The simplest illustration of the full LAG-1 model, and one that may be sufficient for readers not interested in the mathematical aspects of the work, is shown in Fig 1. We embedded the primary theoretical ideas in a model of how the three relevant subsystems (learning, attention, and gaze) might interact. LAG-1 actively samples the visual field, forms associations between categories and features values, and initiates actions influenced by both the sampling and the learning. The labels and connections shown in the figure are meant to broadly conform to what is known about the structure and function of the relevant aspects of the human brain.

### Information flow through LAG-1

In the category learning experiments modelled, the task comprises several hundred learning trials, each of which has two phases: a response phase and a feedback phase. In the response phase, a fixation cross is first shown in order to center the participant’s gaze, after which a stimulus is presented. The participant then views the features of the stimulus, and makes a response by clicking a button associated with one of the categories. The category response then initiates the feedback phase wherein both the same stimulus and the correct category are presented. When the participant is ready to start the next trial, they click a button, and the fixation cross for the next trial appears. Both of the phases of the trial are self-paced: the stimulus and the correct category can be observed for as long as the subject (or the model) wishes.

The way that LAG-1 processes information during the experiment is depicted in Fig 1. At the start of each trial a stimulus is presented to the model as input to the Visual Field. Stimulus features have particular values, coupled to that particular location on the screen, and the model, because it represents everything spatially, is sensitive to the locations associated with particular combinations of feature values. As spatial competition for attention gets resolved by the lateral dynamics of local-excitation and global-inhibition, gaze will shift to a saccade threshold-crossing peripheral location of the Visual Field, and extract the value of the feature there. This information then passes into the Learning System, activating the appropriate Feature Detectors whose self-sustaining activation acts as a kind of visual working memory. Feature Detectors then propagate their activation through a weight matrix and into the Category Neurons. Recurrent connections from the Category Neurons (propagating backward through a copy of the same weight matrix) pass activation to Feature Expectation Neurons that are associated with those active categories. A critical feature of the system is that activation of these neurons is modulated by a gain mechanism that gives additional boosts of activation to features based on their expected information gain. The calculation of gain is explained in detail in the “Category Neurons” section of the Learning System overview. This top-down information is integrated with both bottom-up information propagating in from the Visual Field, and directly on to the Spatial Attention Field, such that active categories are driving attentional choices, and not just adapting what is salient [39]. Activity on the Spatial Attention Field passes into the Saccade Motor Field, which, because of an inhibitory signal at the current fixation location, can be thought of as a map of possible saccade targets. The pressure to initiate a saccade (and thus target the peak location in the Saccade Motor Field) is controlled by a trio of neurons, and builds up slowly from the beginning of a fixation. Strong activation at the location currently activated can delay saccades, and strong activation at potential saccade target locations can speed them up. Because correct categorization requires information about the value of more than a single feature in these experiments, and because only one feature can be fixated at a time, the model must make a series of eye movements, as humans must, in order to gather the information necessary to make a correct classification. As the model views the features of the stimulus, information is sustained by both the Feature Detectors and the Category Neurons, until activation of a motor response crosses a fixed threshold (the Decision Click Neuron); in the human experiments this would be like a mouse click or button press response.

Once a category response has been chosen, the feedback phase of the trial begins. The correct category enters the Visuospatial Processing system as a button that appears in the periphery of the Visual Field. If viewed, a boosting signal excites the Category Neuron corresponding to the correct category. LAG-1 may continue to look at features of the stimulus, boosting their activation among the Feature Detectors, and countering any memory decay for those values that may have occurred since having last looked at them. During the feedback phase the connections between active features and active categories are strengthened according to simple Hebbian learning. The longer LAG-1 studies the feedback, the more the synaptic weights connecting the active features and categories are strengthened. Once the Click Decision Neuron has again exceeded threshold, the next trial is initiated.

As changes to the associative weights between the Feature Detector Neurons and the Category Neurons accumulate over learning trials, the dynamics of the system begin to change. The strength of an association, reflecting base rates, and the category selectivity of Feature Detectors, reflecting information gain, work together to modify top-down attention. These top-down signals decrease the chance that irrelevant information will be fixated, as well as decreasing fixation durations. Increased activation of the Category Neurons, and faster accessing of the useful information leads to faster reaction times. Increased activation of the Category Neurons also leads to less time being spent during feedback. LAG-1 thus reflects the idea that learning influences attention and attention influences gaze, as well as the inverse relation: what is viewed, and for how long, changes what is learned.

LAG-1 has only three fitted individual subject parameters—shown in green in Fig 1: Learning Rate, Fixation Impatience, and Trial Impatience. The Learning Rate modulates how rapidly the connections between Feature Detectors and categories change per unit time. The Fixation Impatience parameter modulates the growth rate of the pressure to initiate a saccade, which is known to be dynamically adjusted to improve reward rate (c.f., [58, 59]), and the Trial Impatience similarly controls the growth rate of the pressure to make a response (i.e., either by clicking with a category response, and thus initiating the feedback phase, or, during the feedback phase, by clicking to initiate the next trial). These parameters have the effect of preventing the model from perseverating on a difficult decision and ensuring a level of global stability. These impatience parameters could be thought of similarly to “urgency signals” found in the speeded choice literature (e.g., [60, 61]): the idea being to keep the system moving by applying a strong compulsion to move the eye or make a decision after a consistent amount of time has elapsed.

### Detailed description of LAG-1 and neurophysiologial context

In this section we provide the formal description of the model, as well as identify research relevant to its structure and functioning. The model structure, illustrated in Fig 1, can be a useful map to the three subsystems, their component parts, and can even help one parse the various model equations (e.g., it can help to compare the inputs in the equation to the inputs shown in the figure). Readers that are less interested in the computational details may still wish to read the overviews provided for the three subsystems to get a feel for how they work. For readers who are interested in the technical details but may be unfamiliar with, or could benefit from a refresher on, the differential equations that form the foundation of the model, we have produced a detailed primer that can be found in the supplementary information: “S1 Appendix: A Primer to Dynamic Neural Field Theory”.

In the supplementary information titled “S2 Appendix: Companion equations for the formal description of LAG-1and neurophysiologial context.”, we provide the equations for each of the components described at a high-level here. Wherever possible we have attempted to give model parameters intuitive labels—for example, the Fixation Neuron is labeled *u*_*x*_, the Gaze Change Neuron is *u*_*g*_, and the Feature Detection Neurons are $u_{{\text{ft}}_{\text{det}}}\left(j,t\right)$. The output of each neuron and field is bounded by a sigmoid function. Values having been transformed by a sigmoid function *f* are indicated with a *, e.g., u* = *f*(*u*). More information about this is, provided in the supplementary information S1 Appendix. Finally, references to structural components of the model are capitalized, such as the Visual Field, as opposed to generically talking about the visual field of view.

Modelling complex non-linear systems like LAG-1 requires that the correct balance be found between the various forces affecting the activity of a neuron or field. In LAG-1 we use *u*_*c*_ to indicate a simple scaling parameter. There are many of these throughout the model equations as they are a convenient way to adjust the relative magnitudes between forces at play in the equation. That being said, once fields/neurons were stabilized, these scaling parameters were not changed. That is, *u*_*c*_ parameters were not adjusted at all during the data fitting process. We think of these parameters as a mathematical (and practical) necessity, but not critical for understanding the work as a theory; for this reason they are not discussed in detail at each occurrence. Values for scaling parameters used in any model equations throughout this paper can be found in the supplementary information titled “S5 Appendix: Parameter tables and best fits.”

#### Visuospatial Processing system overview

Experiment input to LAG-1 is represented as a stack of two dimensional layers having the same size as the Visual Field. The location of a red feature, for example, is represented as a set of 1s among an array of zeros at the appropriate spatial location in the layer that represent red input. LAG-1 extracts information from this input in two places, the Visual Field, which codes the spatial location of all elements in the stimulus regardless of color value, and the Feature Detection Neurons, which code the feature values for any feature that is currently fixated. On the Visual Field, information about the stimulus is coded in retinotopic coordinates, where the fovea centers the frame of reference. At this early stage of processing the featural dimensions of a stimulus are not intrinsically bound with location. Similar to the “what”/“where”, ventral/dorsal, processing streams observed in the brain, LAG-1 must use “complementary processes” that have hierarchical and laminar components to integrate different dimensions of the input into a sensible package [62, 63].

Spatial information from the Visual Field projects into the Spatial Attention Field of LAG-1 where spatial competition for attentional priority resolves. Priority maps in the parietal cortex are known to predict movement behaviours given a wide range of considerations such as goals, reward history, novelty, as well as category decisions and information gain [39]. Parietal priority maps connect those ideas to actual choices to do things in the real world. Activity on the Spatial Attention Field can be thought of as both selective and predictive of eye movements. Where area LIP projects to the SC, and through a thalamic route receives input from the SC, the Spatial Attention Field and Saccade Motor Field in LAG-1 also have bidirectional relationships. In both the model and the brain, these structures are essential to relating overt and covert attentional dynamics [64].

Converting representations between different frames of reference can be accomplished with the aid of a movement shift operator or coupling fields that represent information in differing frames of reference. An earlier DNFT model of saccadic remapping during multiple-object-tracking (MOT), offers a precise approach to the mechanics of reference frame transformations between neural fields [65]. In the present version of LAG-1 we simplify things by directly coding a transformation operator between the spatiotopic frames of reference used by the Spatial Attenion and Saccade Motor Fields, and the retinotopic coordinates of the Visual Field.

#### Visual Field

The Visual Field in LAG-1 is a retinotopic map that represents the locations of features relative to the fovea. The center of the Visual Field defined in Equation 11 is always the fovea, while the activations of the Visual Field are translated in space to reflect their change in distance from the center as the eye moves. Experiment stimulus inputs provided to the Visual Field are transformed by Equation 12 The processes comprised by the Visual Field and the input stimulus transformation are an abstraction of the kind of early visual processes that begin at the retina and culminate at the psychologically interpretable visual categories represented by complex neurons in V4 and IT [66].

#### Spatial Attention Field

Turning knowledge into behaviour at the right place, and at the right time, is a large part of the functions of the priority maps in the parietal cortex [39, 67]. The posterior parietal region of the parietal cortex coordinates a number of motor actions like reaching, grabbing and saccade targeting [68]. Neurons in lateral intraparietal area (LIP) have been shown to vary systematically with the location [69], category [70], reward [71], posterior likelihood [38, 72] and task relevance [73, 74] of individual eye movements. This makes LIP a very important source of attention related projections to more proximate motor control of the eye like the superior colliculus [75, 76].

**Fig 2:**
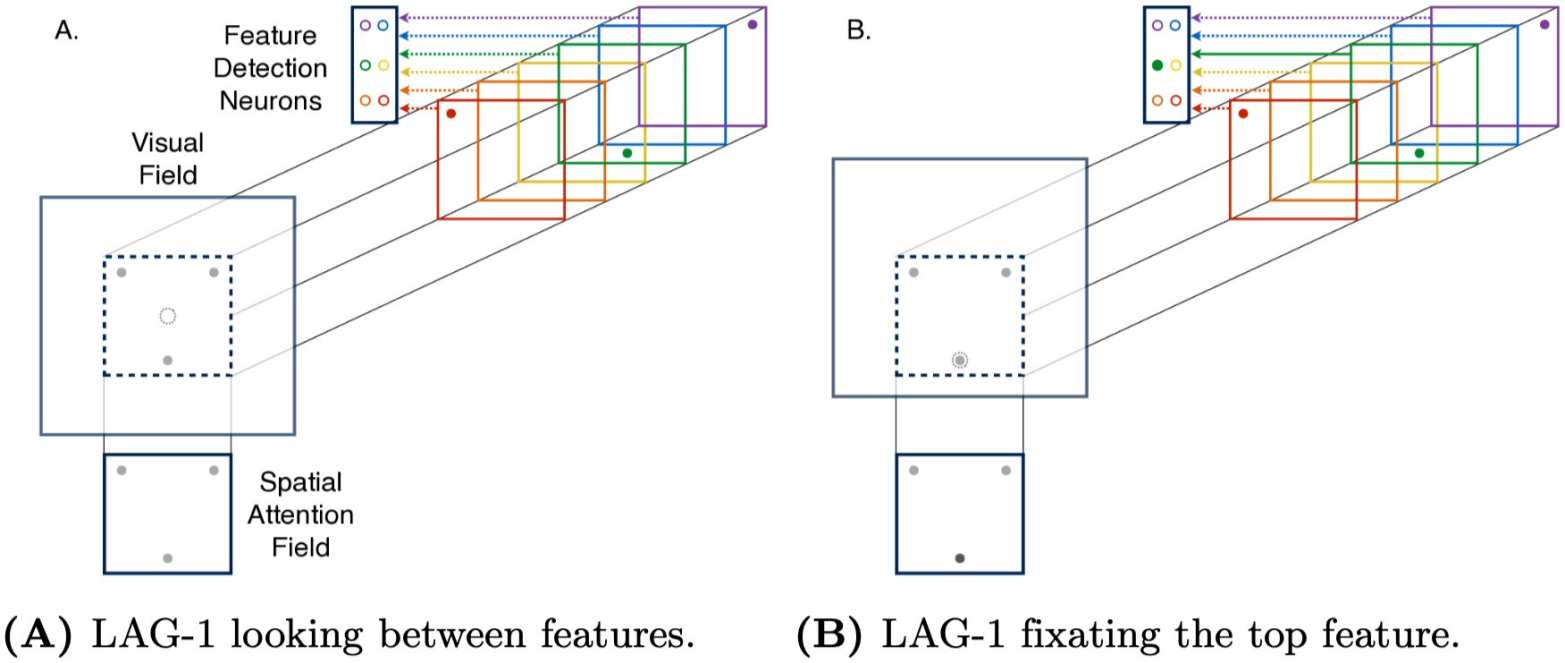
Information processing schema. Schematic of the relationships between experiment input, the Feature Detection Neurons, Visual Field, and the Spatial Attention Field. A) The fovea is indicated by the dashed grey circle in the center of the Visual Field. B) After an eye movement to the bottom middle feature, the green sensitive Feature Detector is activated and the Spatial Attention Field is boosted at its associated location. The Visual Field is coding spatial information in retinotopic coordinates while the Spatial Attention Field is coding information in spatiotopic coordinates.

The Spatial Attention Field in LAG-1 represents changes in attentional priority to locations in a spatiotopic reference frame. The different frames of reference of the Spatial Attention Field and the Visual Field are depicted in Fig 2. Notice that there is no change in the location of features on the Spatial Attention Field between Fig 2A and 2B, despite the shifting of the bottom feature onto the fovea of the Visual Field.

Changes in activation on the Spatial Attention Field are described by similar equations as those used for the Visual Field, in Equation 13.

#### Saccade Motor Field

Candidate saccade target locations compete on the Saccade Motor Field [48, 49]. Unlike the Spatial Attention Field which binds a retinue of competing attentional priorities with spatial locations, the Saccade Motor Field resolves competition for attention at locations *other than* the current locus of fixation. The dynamics of this field resolves the competition between locations to be the target of the next eye movement according to Equation 17.

#### Saccade Timing System overview

Under normal viewing conditions, humans make about three saccades per second [77]. The period of relative spatial stability between saccades is the fixation duration. The parameterizations used in the present simulations were chosen for their rough correspondence with the normal range of saccade and fixation durations. In the model, as in the brain, the timing of an eye movement is affected by previous experience. In people, this includes factors like expected processing difficulty, but category learning experiments are designed to minimize such effects, so changes in fixation duration exhibited by LAG-1 are primarily the result of learning the relevance of different features. We have also observed fixation duration differences in LAG-1 when using it for visual search where disorganized inputs yield spatial interactions that speed or slow its saccade onset latency [40, 46, 58, 67, 78].

As the name suggests, the Saccade Timing System is the primary arbiter of decisions to release fixation and foveate a new location. The trio of neurons controlling this system are: the Gaze Change Neuron, the Fixation Neuron, and the Saccade Initiation Neuron. These model neurons have a functional correspondence with brain stem neurons referred to as: “build-up neurons”, that increase their firing rate over the course of saccade preparation; “fixation neurons”, that remain tonically active during a fixation; and “burst neurons”, that spike just prior to a saccade [79–81]. Again, a saccade is initiated by LAG-1 when the Saccade Initiation Neuron crosses a threshold. When the eye is at rest, the Fixation Neuron inhibits this Saccade Initiation Neuron, and the Gaze Change Neuron increases the pressure to initiate a saccade as the duration of a fixation goes grows.

The Saccade Timing System is influenced by three exogenous inputs. First, the Fixation Impatience parameter influences how rapidly the activation of the Gaze Change Neuron grows, and reflects stable individual differences in fixation durations. That such a parameter could vary systematically between individuals is motivated by timing differences in gaze behaviours between individuals. Individuals prone to making longer fixations will do so across numerous kinds of tasks and this trait covaries with other gaze measures, such as the typical length of their saccades [82, 83]. Second, the Fixation Neuron receives inputs from the Visual Field when a feature is being fixated. Finally learning induced increases in activation for relevant features on the Spatial Attention Field and Saccade Motor Fields push the Saccade Initiation Neuron over threshold faster.

#### Gaze Change Neuron

The Gaze Change Neuron plays a critical role in compelling LAG-1 to explore the visual environment. The behaviour of this neuron, defined by Equation 19, has notable similarities with build-up neurons found in caudal regions of the intermediate layers of the superior colliculus [49]. The Fixation Impatience parameter, λ_*P*2_, increases the influence of these requests with every time step when λ_*P*2_ > 1. Putting the bulk of a saccade initiation decision on to a preprogrammed timer makes these decisions more resilient to noise and moment-to-moment attentional capture. In the real world people can dynamically adjust such timers over the course of a few trials based on factors like the expected processing difficulty or the inter-trial interval [58, 59].

#### Fixation Neuron

The Fixation Neuron, defined in Equation 20, suppresses the impetus to move the eye. It accomplishes this by exciting the foveal location of the Spatial Attention Field, thereby damping extrafoveal locations of the field by way of a stronger global inhibition projection to regions other than the boosted fovea. The only input to this neuron is the sum of featural information within the foveally masked location of the Visual Field. Anatomically, fixation neurons are observed to project tonic inhibition to saccadic burst neurons in the time between saccades, such that inhibiting them allows saccades to be programmed and executed faster [84, 85].

#### Saccade Initiation Neuron

A saccade is initiated by LAG-1 when the Saccade Initiation Neuron reaches threshold, according to Equation 21. This neuron has functional similarities with saccadic burst neurons, which show phasic activity in the moments just prior to a saccade [79].

### Learning system overview

LAG-1 learns during the feedback phase of every trial by Hebbian modification of the weights connecting active Feature Detection Neurons with Category Neurons activated by fixating a correct category presented on the screen. If an incorrect guess was made, anti-Hebbian learning simultaneously decouples the active Feature Detection Neurons and Category Neurons. For example, if a blue, yellow and green set of Feature Detection Neurons is co-active with category A during the feedback phase, applying the learning rule will increase the particular weight that connects the presynaptic Feature Detection Neuron and the correct Category Neuron, proportionate to the current strength of the weight. If the correct category was not A in this example, but B, then the blue, yellow, and green Feature Detection Neurons activated by fixating these features, would reduce their connection strength to A, and increase it to B during the feedback phase.

Attending to feedback signals is an essential part of category learning [30, 37]. The category learning experiments we simulate are self-paced: participants click a response button associated with a particular category which brings up the feedback display, and when they are ready they click again to advance to the next trial. Participants can, if they choose, rapidly click to advance to the next trial without spending time looking at the feedback or stimulus. Human participants who have mastered the categories often choose to do this in order to finish faster—LAG-1 too, has this option. The decision to click and advance the experiment to the next phase is represented by a Click Decision Neuron having two thresholds. Crossing the first threshold moves the trial from the response phase to the feedback phase, crossing the second threshold ends the trial.

At the start of the experiment, when LAG-1 knows nothing about the category and feature relationships, impatience and experiment feedback signals provide the bulk of the input needed for the Click Decision Neuron to cross its thresholds. As knowledge about the category structure develops, this knowledge will result in higher activation of particular Category Neurons, which, when combined with experiment feedback signals, make for faster reaction times in the feedback phase. When the category associations are very strong, feedback signals may be unnecessary for crossing the second threshold, and thus, LAG-1, like the human participants, can stop looking at feedback altogether when it has mastered the categories.

In addition to registering the physical attributes of the features being looked at, Feature Detection Neurons act like a visual working memory in that they can sustain their activation even after looking elsewhere. Each of these neurons is connected to the others, as well as to a set of Category Neurons that do no themselves have a sensory connection to the experiment stimulus except through the the Feature Detection Neurons.

Activity propagates from the Category Neurons into the Click Decision Neuron, as well as feeding back through a mirror of the weights that connect the Feature Detection Neurons with the Category Neurons, to activate the Feature Expectation Neurons. These neurons mirror the Feature Detection Neurons in number but project back toward the spatial locations of the Visual and Spatial Attention Fields where the feature values are known to be associated. This is how LAG-1 allows feature-based attention to influence the selection of eye movements [86, 87].

At the top of the visual attention hierarchy in the brain are category sensitive neurons in areas like inferotemporal (IT) and medial temporal lobe (MTL) which get activated by ongoing sensory combinations of visual input and which have reentrant projections to successive layers of the visual cortex, and categorical projections to LIP respectively. Neurons in these regions effectively sensitize visual neurons according to category expectations [70, 88–92]. Neurons in these regions are reported to have a number of attribute-invariant preferences for all kinds of complex categories like cars and celebrities [93, 94]. When activated, neurons with these categorical preferences alter sensory expectancies across modalities and bias the spatial priority of action competitions resolved in the parietal cortex [73, 95].

Because our primary theoretical interest was integrating learning, attention and gaze, and not replicating a fully developed category learning model we chose to use a relatively simple learning system (cf. [96]). The only weights that are adjusted within each experiment are those between the Feature Detection Neurons and the Category Neurons. This means that LAG-1 is, for now, only capable of linear association, limiting its general ability to test rules and learn categories that are not linearly separable [21, 96, 97]. Implementing a one–level–more–detailed category learning system for LAG-1 would require a richer version of visual feature-based categorization inspired by the functions of IT/MTL/hippocampus [93], reentrant relationships between temporal, visual, and parietal cortices [63, 72, 98], prefrontal cortex for choosing among differing rules and strategies [57, 99], and reward modulated Hebbian plasticity for more explicit temporal difference learning [100–102].

#### Feature Detection Neurons

The Feature Detection Neurons represent the non-spatial attributes of a stimulus. Feature values are depicted in Fig 2 as color patches in different locations of the screen. In the experiments we simulate, the stimulus features are binary valued. That is, each feature can have only one of two possible attributes for a given trial. LAG-1 represents this as two features connected to the same region of space in the Spatial Attention Field and Visual Field, and with inhibitory connections between the two Feature Detection Neurons tuned to each location.

The Feature Detection Neurons, defined by Equation 22, are agnostic to the property of the stimulus they are representing. The color patches we use are a convenience and could be thought of as representing any feature dimension of the stimulus. The color categories represented by the Feature Detection Neurons in our examples could be understood as explicit representations in a psychological color space like that found in inferior temporal (IT) cortex, as easily as any other physical property of the stimulus [66, 103].

#### Category Neurons

Categories in LAG-1 are represented by discrete neurons, as defined in Equation 24, with one neuron for each category in the task. Importantly, the Category Neurons do not have to be directly maintained by the excitatory projections of the Feature Detection Neurons: they can be completely self-sustaining once activated enough. The Category Neurons also compete with one another via the same simple global inhibition as the Feature Detection Neurons.

Activation propagating from Feature Detection Neurons to the Category Neurons is attenuated by gain, defined by Equation 25. The total difference between the synaptic weights connecting the Feature Detector Neurons associated with a particular location, for each Category Neuron can be used as an indicator of the information value of the feature dimension at that location for that category. For example, if Feature 1 and Feature 1’ both have a connection to Category Neuron A of strength 0.5 (thus a difference of zero), then Category A will be equally active, regardless of the value of Feature 1. In contrast, if Feature 1 has a connection of.02 and Feature 1’ has a connection of.98 with Category Neuron A (and thus the difference between them is large) then this location is treated as very diagnostic of this category.

The purpose of gain in LAG-1 is to temper the effects of associative learning based on stimulus base-rates, with a derived measure of information value. It is known that certain LIP neurons represent a kind of gain as in the form of expected posterior log likelihood ratios of reward and information that would be returned by saccades to each location of the field [38, 72, 101].

#### Feature to category association

The weight matrix connecting the Feature Detection Neurons and the Category Neurons, changes as the model proceeds through the experiment. The values *W*(*i*, *j*, *t*) store the strength of the association between Category Neuron *i*, and Feature Detection Neuron *j*, at time *t*. On each time step of trial feedback, these weights will undergo at least two of the three types of associative learning as defined in Equation 26: 1) increasing the weights between active Feature Detection Neurons and the above-threshold Category Neuron, 2) decreasing all the weights proportionate to the average increase stimulus-specific increase (homeostatic or normalizing “decay”), and 3) decreasing the weights connecting the chosen Category Neuron and the active Feature Detector Neurons.

#### Feature Expectation Neurons

At the start of an experiment, LAG-1 has no information about which features are relevant to the categorization task. As the experiment progresses, it learns how the presence of a particular feature value at a particular location predicts particular categories. The way these expectations are translated into changes in behaviour is through the selective activation of Feature Expectation Neurons by the Category Neurons, as well as inhibition projecting from the Feature Detection Neurons. Category Neurons are connected to the Feature Expectation Neurons, defined in Equation 30, using a copy of the synaptic weight values as those that connect the Feature Detection Neurons with the Category Neurons. The recurrent projections from the Feature Expectation Neurons into the Visual and Spatial Attention Fields change the salience/priority of competing saccade target locations.

#### Click Decision Neuron

The activity of the Click Decision Neuron gradually increases over the course of the trial as LAG-1 looks around and activates categories, and as its decision impatience grows. There are two critical threshold events signaled by this neuron. Crossing the first threshold corresponds to the subject pressing a button to select a category, and transitions the trial into the feedback phase, wherein information representing the correct answer appears on the Visual and Spatial Attention Fields. When the Click Decision Neuron, defined in Equation 31, crosses its second threshold, the trial ends. The amount of time it takes to cross the gap from the first threshold to the second threshold is variable, such that high enough impatience and/or category knowledge can push these neuron across threshold quickly enough to count as a “double-click” that skips feedback entirely.

## Simulations

The present work is based on two key ideas. The primary *theoretical* idea that LAG-1 instantiates is that learning, attention, and gaze, connect in a way that allows category-feature associations and expected information gain to influence spatial attention which in turn influences the selection of saccadic targets. This theoretical idea explains why the different subsystems of LAG-1 are connected the way they are. The primary *empirical* idea behind the model, is that the qualitative learning-related changes in behaviour, both gaze and choice, that have been documented in the experiments that we simulate are largely the result of these linkages between systems. Thus we have two key proposals; one is the functional connection between systems, and one is the unified causal source of the variety of choice and gaze behaviours shown in these tasks. If LAG-1, built on our key theoretical connections, produces the learning-related behaviour changes (e.g., that accuracy goes up throughout learning, that more informative features are prioritised earlier within a trial, that irrelevant features are increasingly ignored, that there is a reduction in fixation counts across learning, and so on) in both category learning choices and in gaze behaviours found in human subjects, then we would take that as evidence for both the theoretical and empirical ideas. If the model reproduces only some of the findings, we will take that as evidence that either the theoretical or empirical ideas, or both, require modification. If there are many findings that the model cannot qualitatively reproduce, then we will take that as evidence that the idea is incorrect, or incomplete.

### Breadth and precision of the modelling

It is important to recognize that our primary interest is in the breadth of LAG-1, rather than its precision. That is, we are less concerned about the precise quantitative magnitiudes than we are in seeing if LAG-1’s behaviours (both choice and oculomotor) change with learning the way people’s behaviours do. Our core argument is that relatively simple connections between learning and attention and gaze underlie a wide variety of findings. In an effort to assess this idea in the simulations, we make several modelling decisions which undoubtedly makes getting good quantitative fits more difficult, but which are truer to our project goals.

First we keep the model flexibility low so that we can unambiguously attribute successful simulations to our core claims. The mathematics that allows the model to fixate in two spatial dimensions and run continuously in time are obviously complex, requiring many equations and many fixed parameter values. However, parameters used in the fitting process—those that can actually change the model’s behaviour to produce better quantitative fits—are very few. Our three free parameters (Learning Rate; Trial Impatience; Fixation Impatience) are considerably fewer than comparable models, e.g., [32].

Second, consistent with our claim that the myriad of behavioural phenomena shown in these task are related, we fit a very broad range of findings. Yet, each additional measure we include constrains the model further, and will impact the quantitative goodness of our fits. We note the breadth of LAG-1’s predictions in this task are unparalleled to our knowledge. Quantitatively comparing models that do not fit the same phenomena is not common practice, and there are clear problems to doing so. For example, we note that for data that a model does not address, the likelihood of that data given the model is zero. Under this construal, the broad model *always* wins, even if its fits are terrible. Obviously there is something unsatisfactory here, in that a narrower model may still have value, even if it cannot address every finding that a broad model can. Yet, it seems equally unsatisfactory to quantitatively compare overlapping models only on the datasets to which they both apply: essentially placing zero value on fitting more data.

Finally, we reserve a few measures, leaving them out of the fitting process entirely, so that we can verify the accuracy of LAG-1’s natural predictions. In LAG-1, the connection between attention and learning processes is relatively constrained: both the magnitude of the connection strength between features and categories, and the information value of those connections, are quantities that derive purely from the knowledge contained in one set of associative weights. We reasoned that if this account is correct, then, as long as we captured important neurofunctional relationships, other complex phenomena might emerge from the basic constraints in the model without additional coaxing on our part. Demonstrating that LAG-1 can make appropriate predictions about unfitted measures is an important way of further supporting our core argument.

### Experimental data being fit

Testing LAG-1 requires an experiment in which both learning and gaze are simultaneously measured, and in which changes in learning lead to changes in gaze. As we know of no other process models that target the relationships between learning and attention and gaze found in category learning in this particular way, instead of direct model comparisons, as is often done when looking at very specific effects or representational difference between models, the approach here is to try and match the human data for all the findings and effects listed in Table 1.

In this section, we fit LAG-1 to data produced in two different studies: [10] and [25]. We describe them in brief, here, with a fuller description of the methods available below. We chose the [25] data as it uses a category structure that has been run many times in our lab with different experimenters, stimuli, instructions, base-rate manipulations, information access costs, and response types [11, 26, 27, 29, 30]. The structure elicits the theoretically important behaviour called stimulus-specific attention, wherein attention is allocated differently based on the properties of the stimulus itself. This finding is important because many existing models have one set of attention weights that change only during response feedback, and so cannot change the allocation based on the features of present stimulus. Eliciting stimulus-specific patterns of eye movements will be an important test of LAG-1.

The second data set is from the [10] study using the “5/4” categories of [17]. This eye tracking study was one of the earliest category learning experiments to use eye movements as a confirmational measure of attention weight predictions. The results suggested that prototype models underestimate attention to the least relevant stimulus dimension in the 5/4 categories (e.g. [32, 104]). Though the 5/4 categories have a long, if contentious (e.g. [105]), history in the study of categorization, using it here allows us to ensure that LAG-1 can model both the appropriate learning and generalization data as well as eye tracking measures. Simulating the [10] experiment also requires that LAG-1 process variable-length experiments with different numbers of features, different arrangements of the features, and different numbers of categories than the [25] study. The reaction time data for the response and feedback sections of the data were not available, and so these measures were not fit in Simulation 2, even though LAG-1 automatically produces reaction times when fit to the remaining data.

In Simulation 1, four behavioural measures were fit: accuracy, fixation count, the probability of fixating irrelevant information on a trial, and the average fixation durations, over the experiment. The data on each of these measures was summarized by an initial intercept and a slope of change in the measure over the experiment, yielding a target vector with eight elements for each human subject. In line with our breadth-focused modelling goals we leave several aspects of the human behaviour to emerge organically from the model. Reaction times, within-trial fixation probabilities, and time spent on features during feedback were left out of the fit function, allowing the fits to these measures to emerge from the model organically.

In Simulation 2, we used the individual subject transfer probabilities (for all 16 stimuli), their learning points (the trial number on which the participant began two consecutive error-free blocks), and a measure of the changes in the allocation of attention to stimulus features of high and low diagnosticity. We were unable to precisely reconstruct all of the individual subject data reported in [10] for the attentional allocation component of the fit measure, so we instead used the reported averages in three measures of attentional allocation. The first two numbers were the average starting and ending fixation counts to the individual features. The third component was the difference between the least informative (Feature 2) and the most informative features (Feature 1) on this measure (∼0.3). The target vector for Simulation 2 thus had 19 elements. Again we allow the model to predict several aspects of the behavioural data with Simulation 2: in particular the within-trial fixation probabilities and the overall fixation proportions.

The experiments record all of the participants’ various responses, when they occurred, and the location of their gaze at all points in time. The simulations of the model are similarly data-rich. Likewise, the attentional and gaze processes in LAG-1 are also spatial and the output of LAG-1 is thus just like the raw data from humans. In fact, we use the same computer code for both humans and simulations to convert the raw data to the aggregated and binned measures presented below. This applies to gaze also: just as with humans, raw gaze location described in x,y coordinates is recorded at a time scale set to approximate a 120hz eye tracker, and this is aggregated into fixations using the same modified gaze dispersion algorithm [106] that was used for the human participants in Simulation 1. Once the final best-fitting simulations are produced (see the supplementary information “S3 Appendix: Fitting procedure” for a full description of the fitting procedures, and supplementary information “S5 Appendix: Parameter tables and best fits” for the best-fitting parameter values) the result is a simulation yoked to each human subject: matching both the experimental situation, and, as well as possible, the impatience and learning rate characteristics of that subject.

### Free parameter relationships with model behaviour

**Fig 3:**
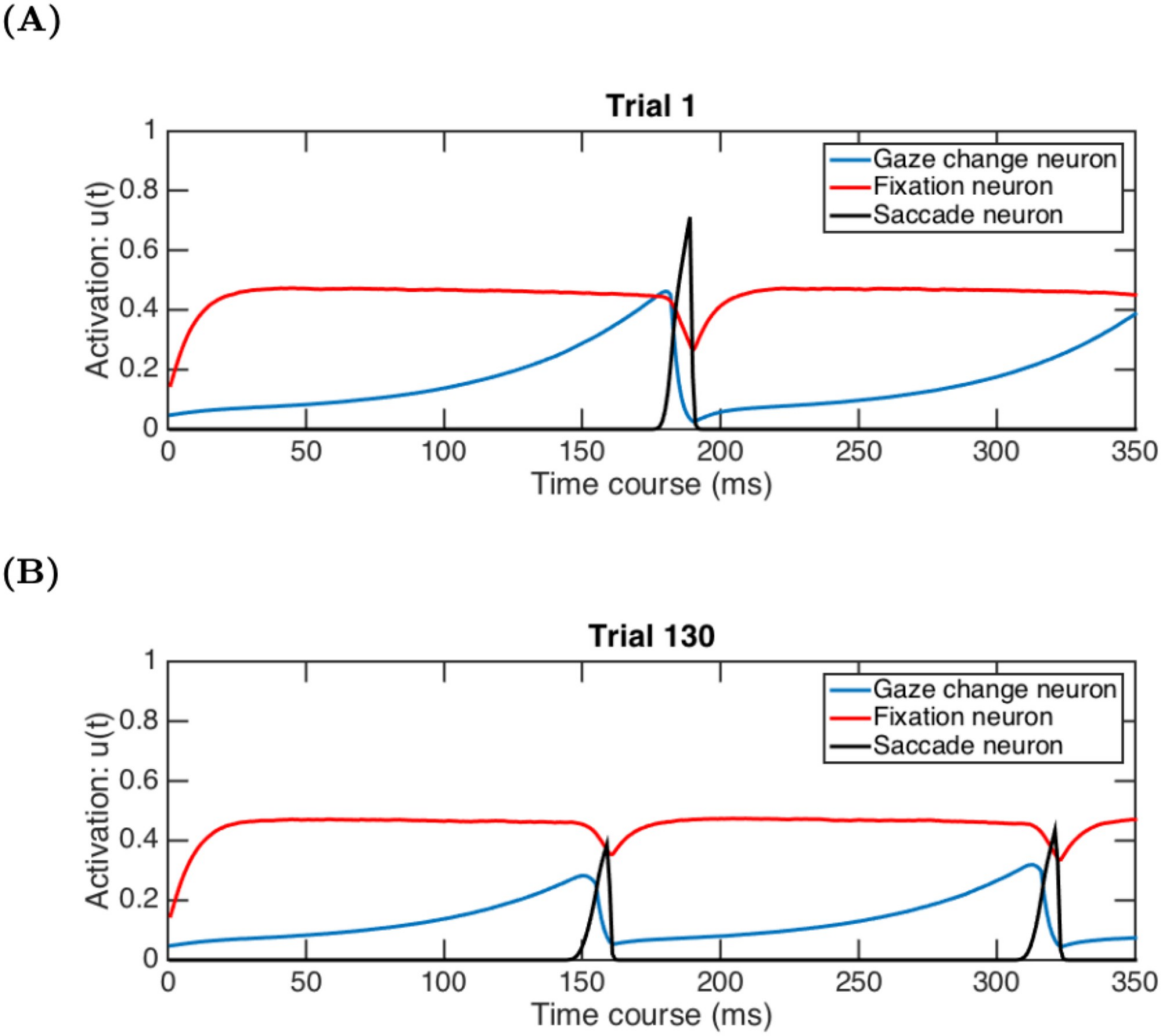
Isolated parameter influences on model. In each of the six figures a comparison of the model’s behaviour is presented at three different levels of the parameters. Each set of parameters is chosen from the closest models available when restricted to the range set by the lowest and highest observed in the best fits of individuals in Experiment 1. In each case, one free parameter is compared while the other two parameters are held to the overall medians observed within that same range. A) Model accuracy over three levels, low, medium and high, of Learning Rate ([2.5 × 10^−6^, 1.1 × 10^−5^, 1.65 × 10^−5^]), when Fixation Impatience is 1.7 and Trial Impatience is 1.8. B) Model fixation counts over three levels, low, medium and high, of Trial Impatience ([1.6, 1.8, 2.05]), when Learning Rate is 1.1 × 10^−5^ and Fixation Impatience is 1.5. C) Model fixation durations over three levels, low, medium and high, of Fixation Impatience ([1.5, 1.7, 1.9]), when Learning Rate is 1.1 × 10^−5^ and Trial Impatience is 1.8. D) Model accuracy over three levels, low, medium and high, of Trial Impatience ([1.6, 1.8, 2.05]), when Fixation Impatience is 1.7 and Learning Rate is 7 × 10^−6^. E) Model feature fixation probability over three levels, low, medium and high, of Trial Impatience ([1.6, 1.8, 2.05]), Fixation Impatience is 1.7 and Learning Rate is 1.1 × 10^−5^. F) Model feature fixation durations over three levels, low, medium and high, of Learning Rate ([2.5 × 10^−6^, 1.1 × 10^−5^, 1.65 × 10^−5^]), Fixation Impatience is 1.7 and Trial Impatience is 1.8. Shading represents standard deviations of the model for that level of parameters.

Before we cover the results of the simulations, it might be helpful to the reader to get a sense of how the model behaves under various values of the three free parameters. Model output at varying levels of the Learning Rate, Fixation Impatience and Trial Impatience is shown in Fig 3. Each parameter combination used to generate the plots is chosen from the storage table of LAG-1 for Simulation 1 output according to the closest models to low, medium and high levels of one of the parameters when the other two parameters are held to the overall medians observed in the individuals. Fig 3A shows how Learning Rate modulates accuracy by simultaneously plotting LAG-1’s learning curves given low, medium, and high Learning Rates, taken from the best fits of the 42 subjects of Simulation 1. The free parameters map onto behaviour in relatively straightforward ways. As the Learning Rate increases, the connections between the Feature Detection Neurons and Category Neurons change more rapidly, leading to improved accuracy. In Fig 3B, as the Trial Impatience increases, the activation of the Click Decision Neuron grows more rapidly, resulting in faster decisions, and therefore fewer fixations. Finally, as the Fixation Impatience parameter increases, as shown in Fig 3C, activation of the gaze change neuron grows more quickly, resulting in shorter fixations.

However, complex interactions amongst the parameters are also important, and meaures of accuracy, fixation duration, and fixation count, can be influenced by changes to the other parameters as well. In Fig 3D, the Trial Impatience parameter strongly influences the learning curve by scaling the amount of time spent on feedback. Fixation counts in Fig 3E are influenced by Fixation Impatience because they change the number fixations that can occur within a particular interval of time. Fixation durations may moderately shrink over the course of experiment as learning increases the recurrent input to the Spatial Attention Field, eliciting the onset of saccades more rapidly. But to underscore how complex the predictions owing to these interactions can be, in the final plot, it is only when Fixation Impatience is high, that this decline is noticeable in Fig 3F.

### Simulation 1

**Fig 4:**
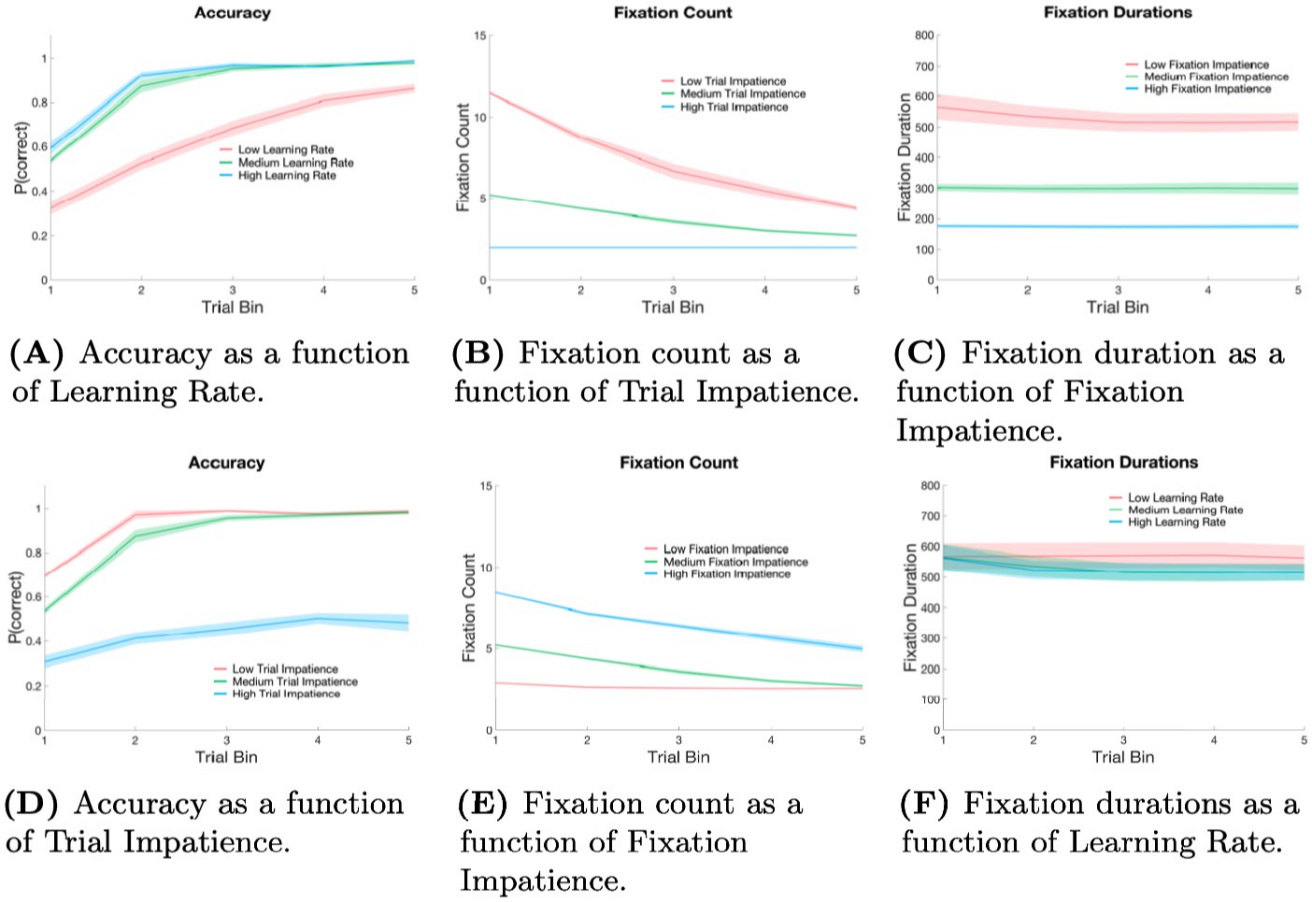
Simulation 1: Experiment and model stimuli examples. A) An example stimulus from the response phase (top) and feedback phase (bottom) from [25]. Each feature type subtends approximately 1.7° of visual angle, separated by 10.6°. B) The features as they look to LAG-1 during the response phase (top), and feedback phase (bottom) showing the location of the feedback button. Features are represented as simple color patches (top right). The feedback button provides the correct answer when foveated by the model. Also depicted by a solid black outline top the top right feature is 1 standard deviation of LAG-1’s fovea, given a fixation to the top right hand feature, where the dashed line reflects 2 standard deviations from highest acuity. Stimuli are counterbalanced between subjects, such that features appear at different locations and have differing relevance.

The eye tracking and category learning data for the first set of simulations comes from the publicly available data set originally reported in [25]. Participants in this experiment were instructed to classify fictional microorganisms, defined as having three critical organelle features (see Fig 4). Each feature could take on two possible values, yielding eight stimuli in total, which were associated with four different categories (A1, A2, B1, B2). Features are arrayed in one of three locations. As is typical for category learning studies, the assignment of image pairs to feature dimensions (feature one, two, and three), and features to locations was roughly counterbalanced across participants. For LAG-1, we simplify the stimuli from complex cell organelles down to three colored features with two possible values, each with its own layer contributing input to the Visual Field (see Fig 2). After making a decision, the participants were given feedback presenting the stimulus again, their own response, and the correct response. Only participants who had at least 70% of their gaze collected in total were included for fitting, and only trials having more than 75% of gaze collected were included in the analyses. Participants had to meet a learning criterion of 24 trials in a row correct in order to be included, leaving 42 individuals in the data set. Of the 480 trials of data available from each subject, we look at only the first 360 in order to reduce simulation time; human behaviour changed very little after that point. More comprehensive details of this experiment can be found in the original paper and on the data repository website.

Stimulus-specific attention—the useful finding that emerges from this category structure—occurs after the participants learn to classify the categories correctly. As can be seen in Table 2, Feature one is always useful, in that it can tell the difference between categories A and B. Features two and three are contingently useful: that is, if Feature one takes a 0 value, then Feature two is important for telling the difference between A1 and A2, while Feature three is no help at all. Likewise, if Feature one has a value of 1, then Feature three can say whether the stimulus is a B1 or B2 but Feature three is irrelevant. Participants can, and do, learn this: the data of within-trial fixation probabilities after learning this category structure show that Feature one is the most likely to be fixated early in the trial and the second most relevant feature for that stimulus, either Feature two or three, is most likely to be fixated later in the trial. Models using a single set of weights cannot shift attention depending on the currently viewed stimulus within a trial, and so cannot account for this finding [11, 16, 107, 108], but some models can account for this at an aggregate feature level [109–111].

**Table 2:** Category structure used in [25].

Feature 1	Feature 2	Feature 3	Category
0	0	0/1	A1
0	1	0/1	A2
1	0/1	0	B1
1	0/1	1	B2

The two values that each feature can take on are represented by 0s and 1s. If a feature is irrelevant for correct classification of a category, it is represented in the table by virtue of being either 0 or 1, denoted as 0/1.

### Simulation 1 results—direct fits

We first look at model performance on measures that we fit directly—that is, some aspect of the measure was a component of the fit function. These basic measures reflect the learning of the categories, and the corresponding changes in the allocation of attention.

#### Learning curves

**Fig 5:**
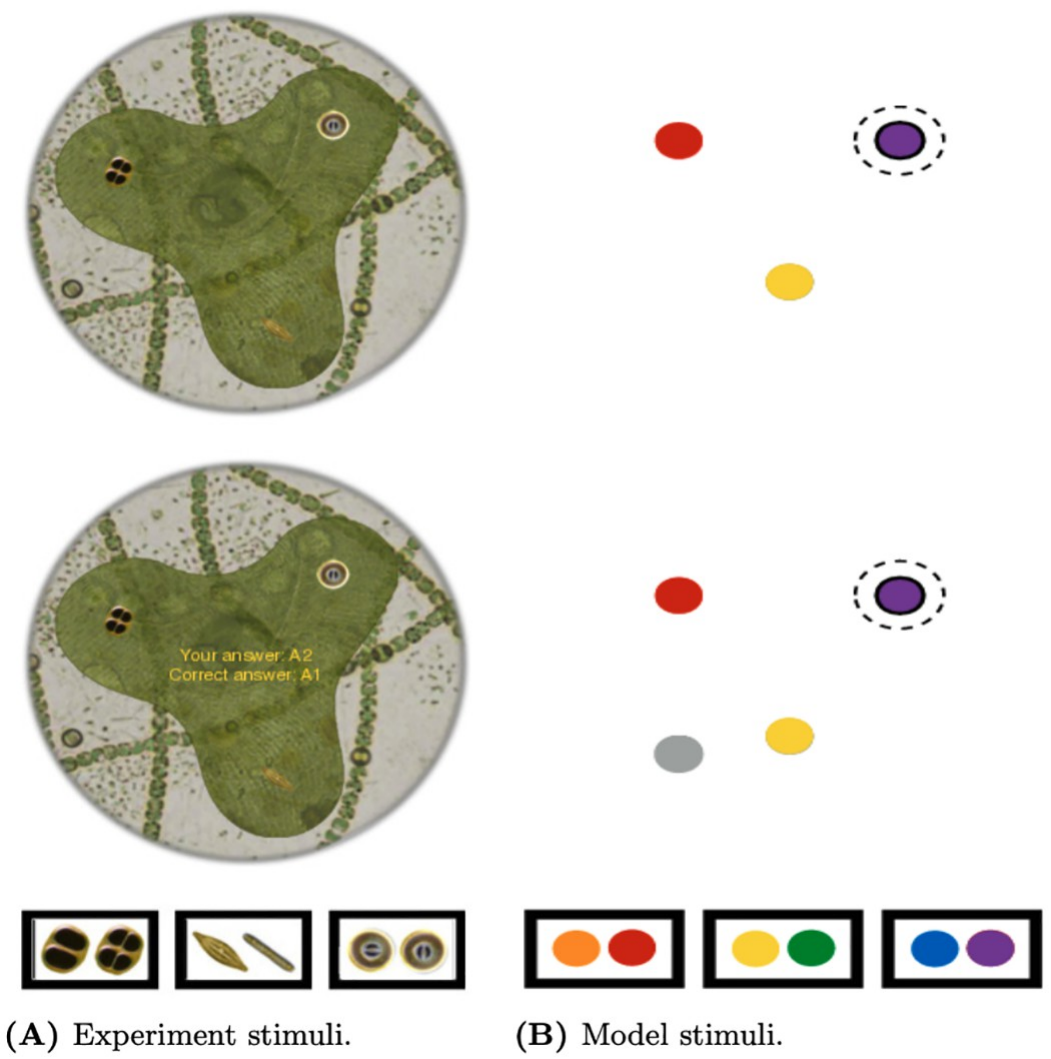
Simulation 1 model fits. Each measure is depicted over 360 trials averaged into 24 bins of 15 trials. Color shading represents standard error of the mean accumulated from individuals and population. A) Trial accuracy means and variability are fit well. B) Fixation duration variability is fit well but the final late reduction means are diverge. C) Fixation count means, variability and change are qualitatively fit well. D) Probability of fixating the irrelevant shows roughly the same change and variability over the experiment, however the scale is off by about 25% at all points. E) Reaction time means, variability and change are qualitatively fit well. F) Total time looking at features during feedback has roughly the right change and variability but the scale is off by 50% at all points.

The first measure we look at is classification accuracy. Chance performance in this task is 25%, setting a baseline from which a learner will increase over the course of the experiment. Fig 5A shows the averaged learning curve for both the model and the human participants’ learning performance.

#### Fixation durations

Fixation durations reflect an important aspect of attentional optimization: look too fast, and crucial information can go unnoticed (e.g. [112]), look too slow and you waste time, potentially slowing learning down at the trial level. The mean fixation durations for the human subjects, and the accompanying model fits seen in Fig 5B, are just above 300ms. These durations typically decrease over the course of the experiment. Similarly, LAG-1 produces fixation durations that decrease across the experiment.

#### Fixation counts

The total number of fixations on a trial is one of the more obvious indicators of task efficiency. As seen in Fig 5C, the human data show a general trend to start 6 fixations per trial and finish between 3 and 4 fixations per trial. LAG-1 starts with roughly 7 fixations per trial, and by the end is indistinguishable from the human data.

#### Fixating irrelevant information

Another method of describing the optimization or efficiency of attention is the probability of fixating an irrelevant feature over the course of an experiment [10, 26]. The data in Fig 5D compares model and humans on this measure, the measure of which is calculated by assigning a 1 or 0 to each trial based on whether or not the irrelevant feature was fixated. Under our current best fitting parameters, LAG-1 has a bias to look at the irrelevant feature more often at the start of the experiment than humans do. In fact, at the start of the experiment, LAG-1 almost invariably looks at all features, and so is more likely to fixate all features, not just the irrelevant one. The finding that people do not attend all features at the beginning of learning is not unique to this study: many previous studies have reported similar findings, though typically not as extreme, showing that some features are not being fixated even when the fixation count is high [10, 25, 26]. There could be a number of explanations for this. One possibility is that participants are engaging in rule testing strategies such that they fixate only one or two features. Previous research has observed a tendency for participants to start by using simple, unidimensional rules in similar tasks [113, 114]. Regardless of the real cause, it is clear that participants in the experiments here just do not look at all the features the way that LAG-1 does, and some adjustment to the model will eventually be needed to better fit this human data.

### Simulation 1 results—indirect/predictive fits

In this section we look at three additional measures which were not in the fit function at all. These measures vary from being moderately constrained by the basic attentional behaviours (reaction time is related to fixation durations and fixation counts, for example), to being mostly unconstrained in its fit to fixation durations and counts (e.g., within-trial fixation probabilities, including the important stimulus-specific attention finding).

#### Reaction times

Reaction time measures can give some insight about attention in category learning (e.g. [31, 115]), and have been modelled before using a variety of methods (see [32–34]). The reaction time data in Fig 5E looks like what one would expect: the responses of both the human participants and LAG-1 speed up with experience. In LAG-1, learning increases the input from the Category Neurons that drive the Click Decision Neuron. The model reproduces the general trend of decreasing reaction times across the experiment, and the model, like the humans, begins at about 4 seconds. The model is slightly too fast by the end, and generally has less variability than the human participants. In humans there are of course additional processes that will affect reaction time that are not captured in LAG-1, such as mind wandering and exogenous distractions, limiting the variability that can be fit on this measure.

#### Within-trial fixation probabilities

**Fig 6:**
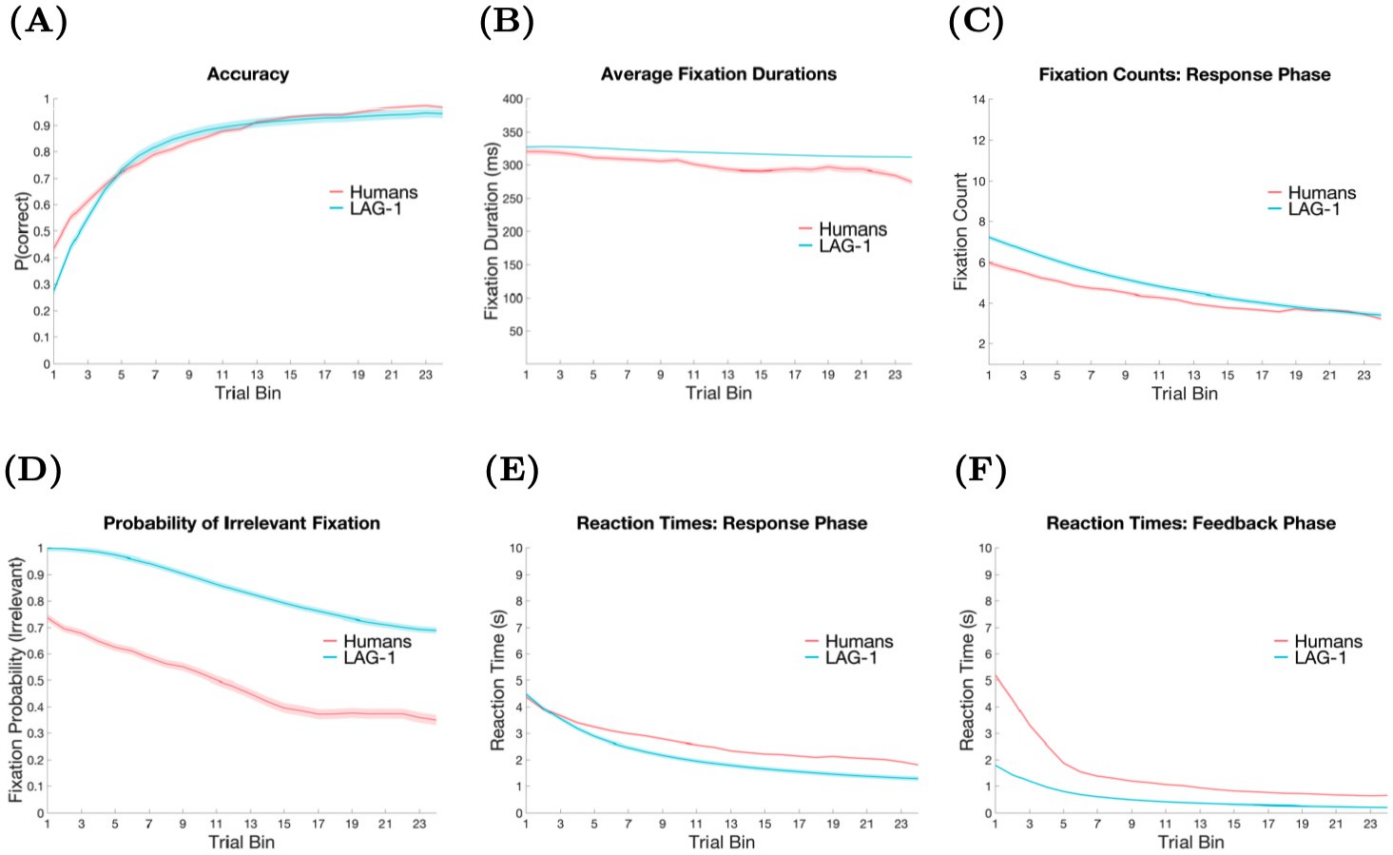
Within-trial feature fixation probabilities. Within-trial feature fixation probabilities averaged across all post criterion point trials and scaled to 100 data points for each trial and averaged into five bins. A) Human data for A categories and with LAG-1’s comparable performance in B. C) Human data for the B categories with LAG-1 again beside it in D. The three important features of the data are: the primacy of Feature one through most of the trial; the primacy of the alternative feature at the end of the trial; and the change in the identity of the second feature across categories (For Category A stimuli it is Feature two, and for Category B stimuli it is Feature three). LAG-1 qualitatively captures all three findings.

Within-trial feature fixation probabilities have previously been reported as another way of quantifying attentional priority [10, 11]. This measure, used in Fig 6, reveals the general order in which the features are fixated. Note that this measure describes within-trial behaviour, whereas all of the data used in the fitting procedure are at the level of the changes across the experiment; there is nothing in the fitting procedure that rewards approximating this within-trial data. The category structure is designed to elicit different optimal fixation orders for the A categories (see Fig 6A for the human data and Fig 6B for LAG-1), differently than the B categories (Fig 6C for the human data and Fig 6D for LAG-1). The trends observed in the participants when looking at just the features of interest (where at any given point in the trial the total probability sums to unity—fixations outside AOIs are excluded from these data for both humans and simulations), show that for the A categories, there is a Feature one precedence, followed by Feature two, and the irrelevant Feature three dropping in probability over the trial. For the B categories Features two and three are flipped as a result of the change in feature relevance. These same trends are present in LAG-1. As before, the model fixates irrelevant information a bit too often, and thus the differences are less dramatic.

#### Time spent on features during feedback

Fig 5F reports the average time spent looking at features of the stimulus during the feedback phase of each trial over the course of the experiment. LAG-1 captures the qualitative trend after this initial large decline. Studies of feedback during category learning are rare [30, 37] and we do not yet know enough about the processes involved, or how they change as knowledge about the task grows, to make strong empirically derived recommendations, but it would appear that LAG-1 offers a straightforward way to account for some of the variance of this measure.

#### Individual fits

**Fig 7:**
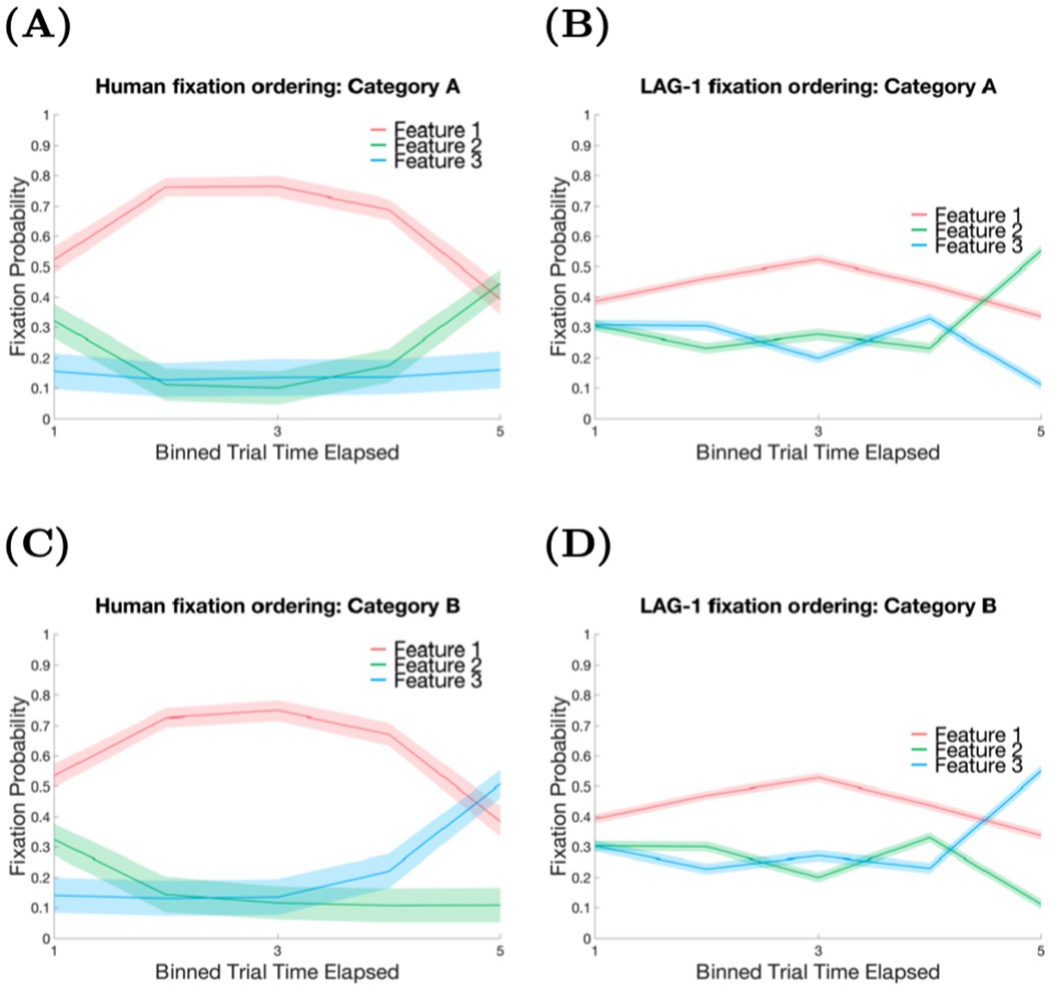
Fits to individuals. Data from three individual participants for fixation count, fixation duration, and accuracy. Below each participant are LAG-1’s fits to that participant’s data. Though the fits are not perfect in all cases, the model is showing both more and fewer fixations (counts), shorter and longer fixations (in milliseconds), and slower and faster learning (proportion correct) in accordance with individual differences.

The fits shown thus far have been averages across many individual participants and simulations. To give the reader a better sense of how LAG-1 addresses differences across individual participants, we show fits to three individual subjects chosen for their differences in behaviour. Amongst these three participants, there is fast and slow learning, short and long fixaton durations, and many and few fixations per trial. Fig 7 shows data for three human participants and for the several model simulations run for each participant. The individual fits for the remaining participants can be found in the supplementary information beginning at Fig 21, in “S6 Appendix: Individual fit visualizations.”. Overall LAG-1 seems responsive to individual differences, and produces large and small numbers for counts, durations, and accuracy. As far as individual fits, there are no obvious peculiarities, though, for some participants, LAG-1 fails to fit the magnitude of a measure, or its slope. As in the aggregated data, it is clear that LAG-1 is not capturing the variability of human data, though it seems better for accuracy than for the other measures. We kept noise relatively constrained for these processes, but additional noise, for Trial Impatience in particular, seems warranted. We note, though, that some of this variability is extreme and seems more likely to be strategic, or to be related to phenomena like mind-wandering [116] than to be a matter of simple noise.

### Simulation 2

**Fig 8:**
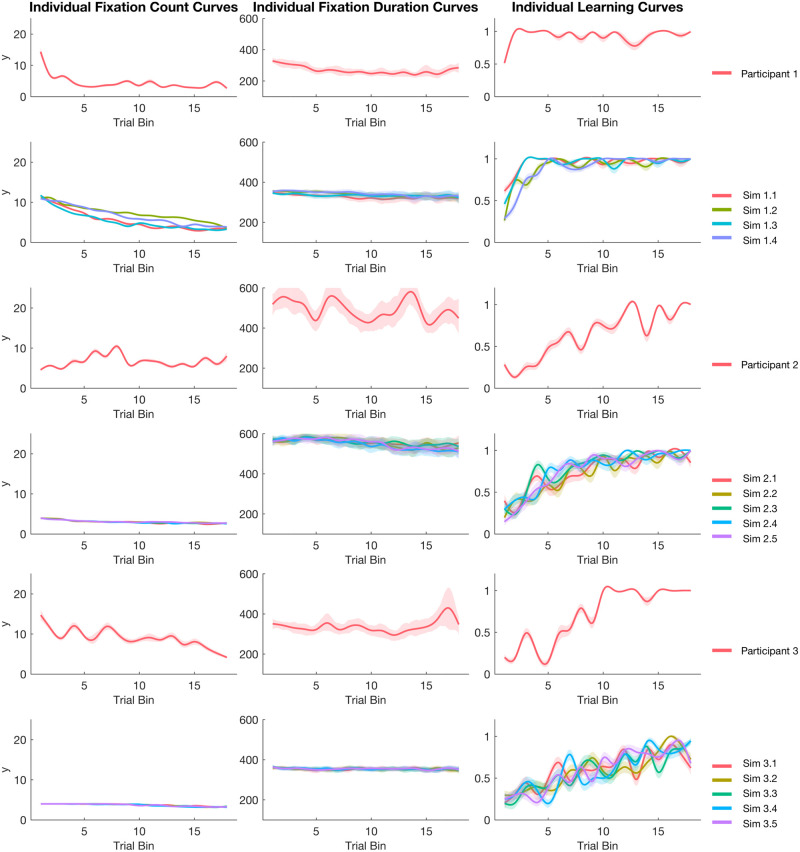
Simulation 2: Experiment and model stimuli examples. A) Example stimulus used by [10]. The four features of interest were reported to subtend approximately 4° of visual angle each, with the stimulus height and width being 12°. B The four color features, spaced 24 spatial units horizontally and vertically and 34 spatial units diagonally, presented to the model.

The second simulation is of response data and eye movement data from [10]. Participants in this experiment were instructed to classify fictional schematic drawings of insects, defined as having four diagnostic features (see Fig 8). Two of these features were highly diagnostic (and equivalently so) of the category, one was of medium diagnosticity, and one of low diagnosticity. Each feature could take one of two possible values, yielding sixteen stimuli in total. Nine stimuli were shown during training and were associated with two categories, leaving seven for classification during a feedback-free transfer phase (see Table 3). After choosing a category, the participants would get feedback, and see the stimulus again. After 189 trials or 18 correct trials in a row, whichever came sooner, a transfer phase would begin that twice tested all of the stimuli, including the seven previously unseen stimuli. There were 64 participants that met the learning criterion with minimal loss of gaze data. Further details of this experiment can be found in the original paper.

**Table 3:** Category structure used in [10].

Feature 1	Feature 2	Feature 3	Feature 4	Category
1	0	1	1	A1
0	0	1	1	A2
0	1	1	1	A3
1	1	1	0	A4
1	1	0	1	A5
1	0	1	0	B1
1	0	0	1	B2
0	1	0	0	B3
0	0	0	0	B4
0	1	1	0	T1
0	0	1	0	T2
1	1	1	1	T3
0	0	0	1	T4
1	1	0	1	T5
0	1	0	1	T6
1	0	0	0	T7

Feature values of 0 and 1 represent the two values of each feature can take on. Comparing the feature values to the categories will reveal that Feature one is of low diagnosticity, Feature two is of medium diagnosticity, and features three and four are of high diagnosticity, relatively speaking.

The best fitting models in Simulation 2 were found by comparing individuals with LAG-1 on 2 subject-level measures and 2 population-level measures. Where Simulation 1 had 8 components to the error function, Simulation 2 uses the 16 transfer responses of each individual, as well as their individual learning speeds, and the average fixation counts at the start and end of the experiment, for a total of 19 components as the objective function (weights listed in Table 8 S5 Appendix). Because we did not have individual data for all measures, we do not show fits to individual subjects as we did in Simulation 1.

### Simulation 2 results—direct fits

Again, we first look at model performance on measures that we fit directly—that is, some aspect of the measure was a component of the fit function.

#### Transfer probabilities

**Fig 9:**
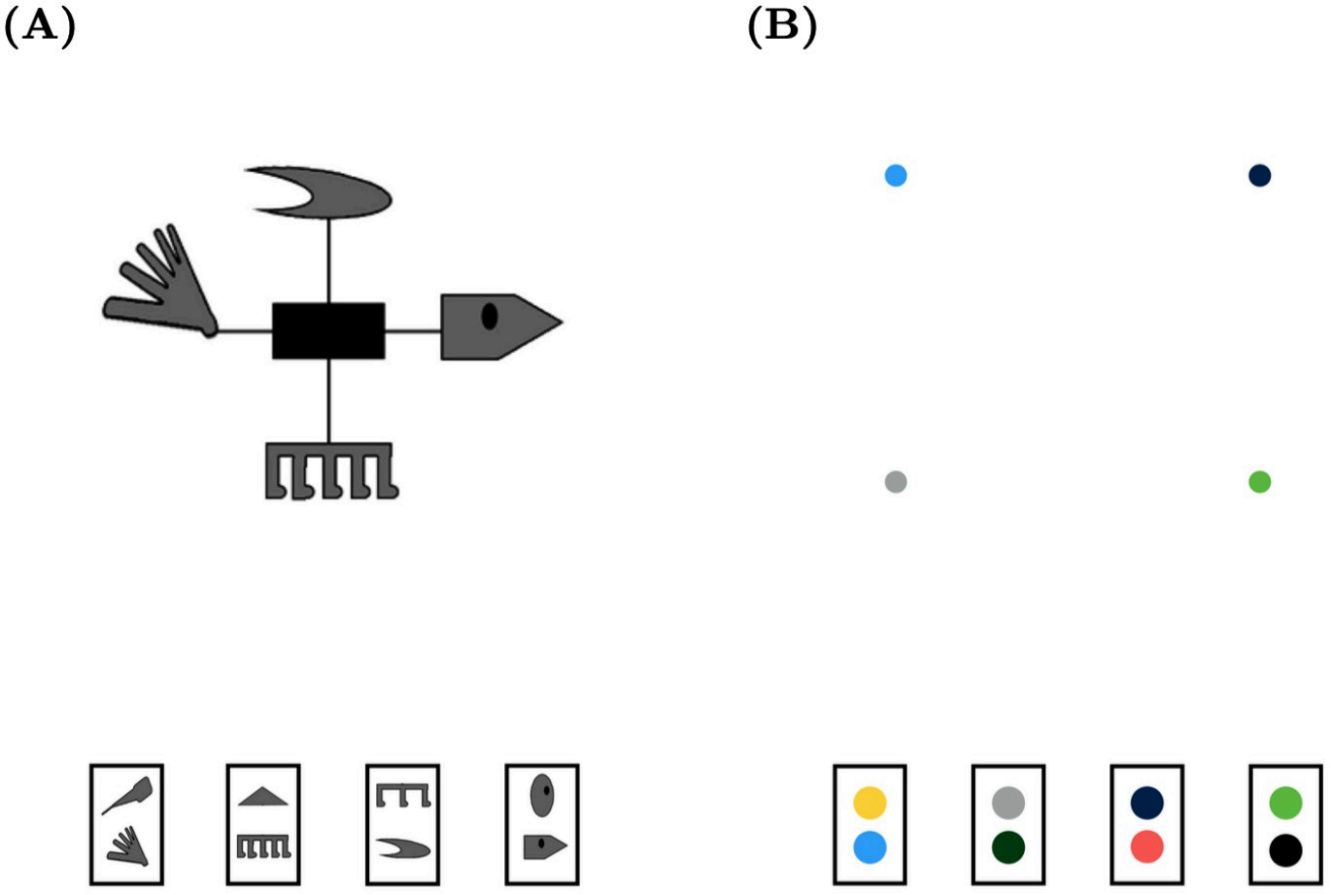
Simulation 2: Model fits to transfer responses. Average categorizations on the 16 transfer stimuli, over all models/subjects. Error bars represent standard error of the mean.

The first measure we look at is transfer behaviour. Fig 9 shows the transfer responses for both human participants and our LAG-1 simululations. These responses are to both trained A and B category stimuli, as well as the seven transfer stimuli not presented during training.

#### Fixation probabilities and counts

**Fig 10:**
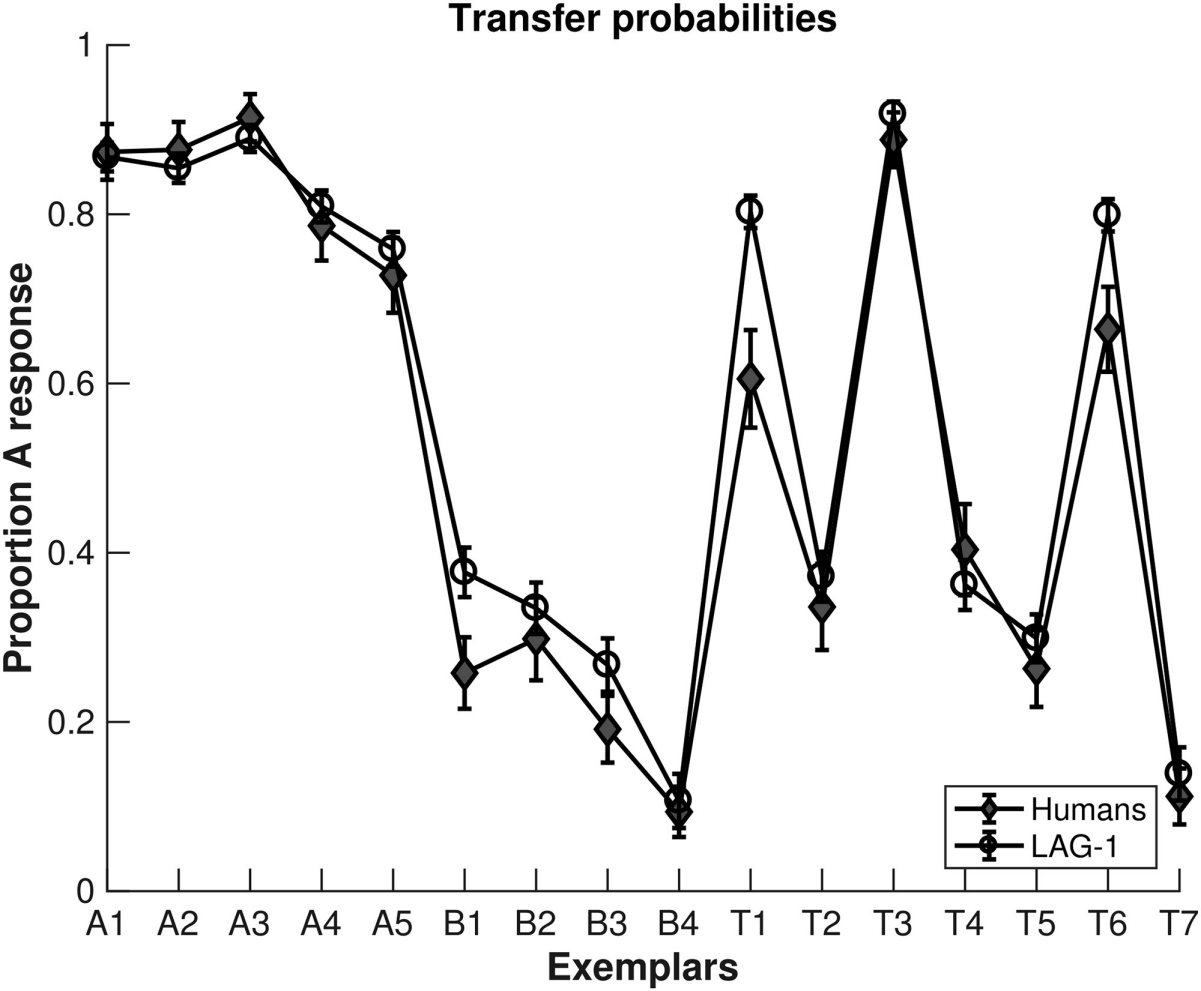
Simulation 2 model fits. A) The probability of fixating a particular feature reported for each of feature observed in human subjects. B) The probability of fixating a particular feature reported for each of feature observed in the best fits of LAG-1. C) The fixation counts to each feature averaged over all trials observed in human subjects. D) The fixation counts to each feature averaged over all trials observed in the best fits of LAG-1.

Trial-level fixation measures reported in [10], are presented in Fig 10 along with the comparable output from the best fitting LAG-1 models. It is clear that by the end of the experiment human participants are fixating the least diagnostic dimension, Feature 2, less often and for less overall time than the other features. The fixation probabilities to each feature are generally higher in LAG-1, just as they were in Simulation 1 (as indicated by the probability of fixating the irrelevant feature measure over the course of the experiment). However, LAG-1 does show analogous reductions in fixation probability and fixation counts, to the least-diagnostic feature.

### Simulation 2 results—indirect/predictive fits

#### Fixation proportions

**Fig 11:**
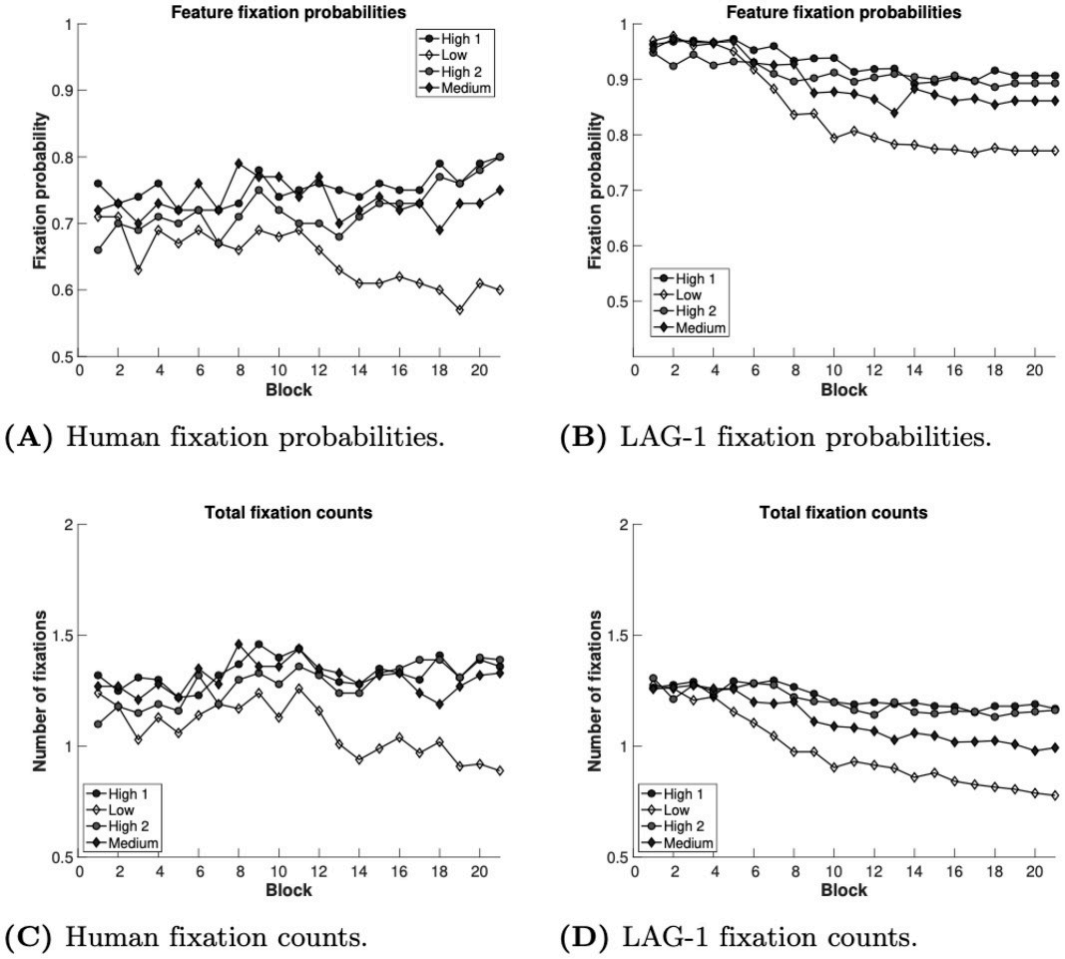
Simulation 2: Fixation proportion fits. The fixation proportions aggregated across just the transfer trials of the experiment. LAG-1 data is on the left, while the data from [10] are re-represented on the right.

As the purpose of [10] was to use an overt empirical measure of attentional optimization to test model predictions, one of the measures they reported was the proportion of time spent on each feature in the transfer phase of the experiment (see Fig 11). Most category learning models are fit with a capacity-constrained level of attention, meaning that on any trial the weights to each dimension are normalized in some way; this means that the total time on each feature has to be represented as a proportion. In our simulations, LAG-1, like the human participants, reproduces the rank ordering of the features by diagnosticity, with high diagnosticity features receiving the most observations, then medium, then low.

#### Within-trial fixation probabilities

**Fig 12:**
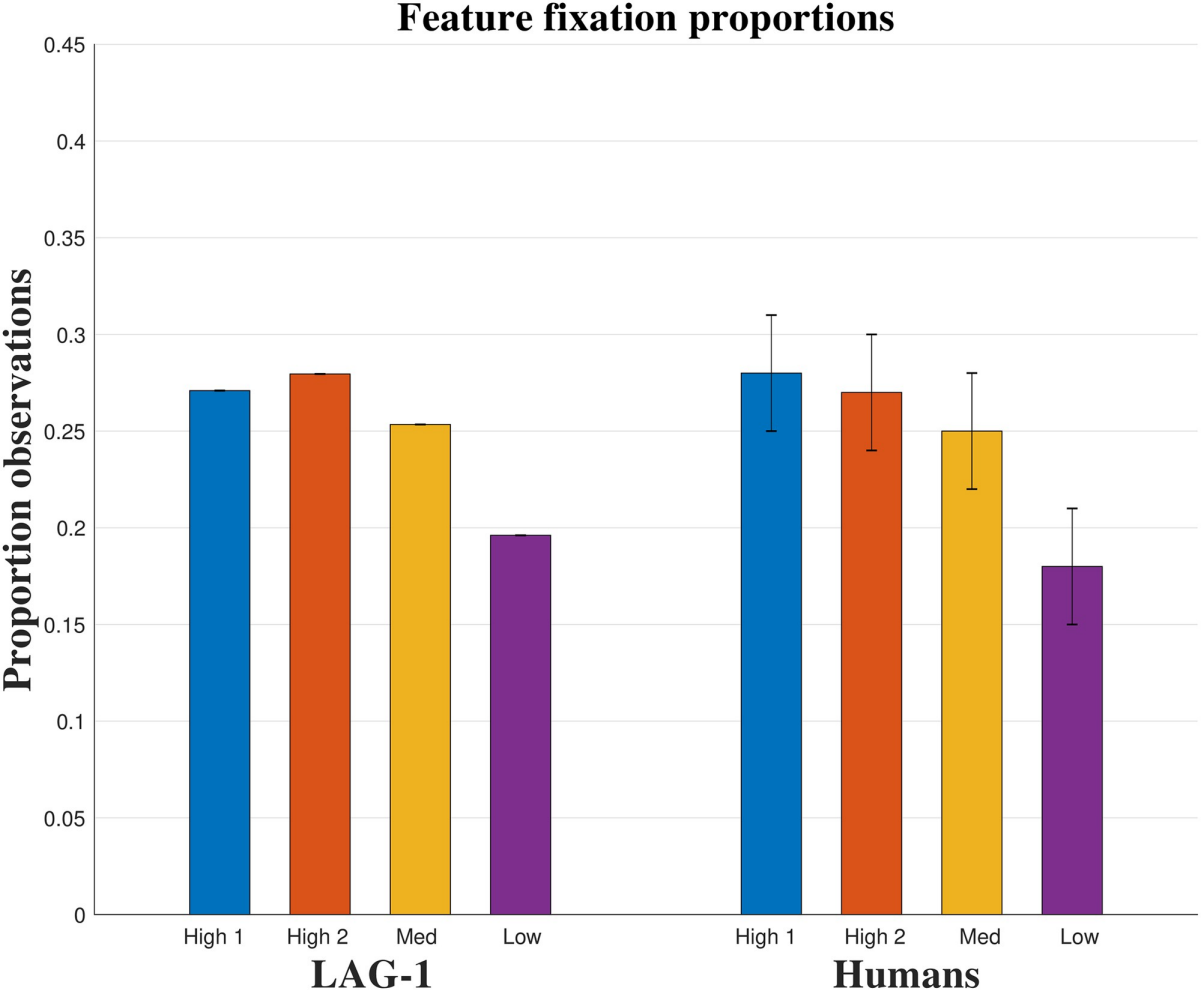
Simulation 2: Within-trial fixation probabilities fits. A) Human within-trial fixation probabilities averaged over the first four seconds all transfer trials reported in Rehder and Hoffman (2005). B) Comparable within trial fixation probabilities for LAG-1. High (relevance) 1 refers to Feature 1 in Table 3, Low is Feature 2, High 2 is Feature 3, and Medium is Feature 4.

The last measure we report here is similar to the within-trial fixation probability measure that we reported in Simulation 1. In Fig 12 the first four seconds of LAG-1’s average within-trial fixation probability to each feature is reported alongside the human data presented in [10]. LAG-1 reproduces the approximate ordering of peak fixations to features of differing diagnosticity: the higher diagnosticity features have peak fixation probabilities earlier than less diagnostic features. The lowest diagnosticity feature is fixated much less often in the first few seconds of the trial for both humans and the model. As in Simulation 1, we do not use within-trial information as part of the fitting function; that and, the largely arbitrary relationship between fixation orders and the overall category structure, make these data difficult to fit precisely.

## Discussion

Our goal in developing LAG-1 is to provide an account of the integration of learning, attention and gaze. In this model, as in the brain, knowledge about where to look emerges from a hierarchy of reentrant visual and categorical processes [88, 90, 117]. LAG-1, as a theory, proposes that the expected information gain can be derived from simple learned associations and used to guide attention. This information is integrated with bottom-up visual signals in a priority map, from which the entire space of saccade target locations is considered simultaneously. The competitive dynamics of this field change both the saccade location choices and saccade onset latencies, thus illustrating an actual mechanism for changes in gaze patterns and changes in fixation durations over learning. Using the model’s three free parameters (Fixation Impatience, Trial Impatience and Learning Rate) we fit LAG-1 to human data [10, 25] from two well established and replicable category learning tasks [11, 17]. We fit classic learning measures (i.e., learning curves, transfer probabilities, reaction time), as well as indicators of attention (i.e., feature fixation proportions, fixation count, fixation duration, fixation orders, feedback processing time), many of which have never before been modelled. The aim of our simulations was to test the idea that the diverse learning-related phenomena found in participant behaviour emerged from interactions such as those that LAG-1 embodies. If the model’s behaviour changes across learning in the same qualitative ways that human participants’ behaviour does, across all the measures, it would provide support for our hypothesis.

Simulation 1 used data from [25] based on the category structure first used in [11] to demonstrate stimulus-responsive attention. This structure is important because the relevance of the features depends on the category to which the stimulus belongs, meaning that using a single attentional strategy for all stimuli is inefficient. Participants have been shown, via eye tracking, to increasingly ignore the stimulus features that are, for that stimulus, irrelevant. LAG-1 ably captures learning curves and reaction times across the experiment. Further, it simulates all the qualitative eye tracking findings previously reviewed in Table 1. Fixation durations, fixation counts, and probability of fixating irrelevant features all decrease as the model learns. The model naturally predicts the correct relative within-trial fixation probabilities, and, importantly, shows different fixation probabilities for different categories, thus displaying stimulus-responsive attention [11]. Finally, the model correctly predicts a reduction in the time spent viewing stimulus features during the feedback phase [30].

In Simulation 2, we worked with data published by [10] using the 5/4 categories of [17]. This structure is important because of its broad use in the field, and one of the key contributions of Rehder and Hoffman’s paper is confirmation, via eye tracking, that, as predicted by the generalized context model even the least discriminative dimension still receives a moderate attention weight [10, 18]. In this simulation, LAG-1 qualitatively matched the transfer responses that have been replicated many times. Further, the model qualitatively captured the eye movement data: more fixations for features that were more diagnostic, and predicts similar within-trial feature fixation probabilities. Importantly, the model also predicts [118]’ key finding, that the least diagnostic information nonetheless receives a moderate amount of attention.

Support for our claim that human learning and gaze behaviours in category learning tasks arise from a system similar to LAG-1 derives from our ability to fit the participants data with the model. Our claim is strongest if we: a) use fewer rather than greater numbers of free parameters, because then our fits flow from the structure of the model and not mathematical flexibility provided by additional parameters; b) fit more findings rather than fewer findings, because it strengthens our argument that all gaze related behaviours at multiple timescales flow from these core principles, and c) fit some aspects of the data indirectly, without using them as part of the fit function, because it speaks to the inevitability of these effects given a system of the kind we proposed, and guarantees that the findings are not due to the fitting process, but are instead endemic to the model. We interpret our results as strong support for our claims because learning influences gaze in the way the model predicts in all cases. But, while these three modelling decisions are appropriate to make the strongest possible claim, they also undoubtedly hinder our ability to get the magnitudes exactly right. We believe our choice to focus on modelling breadth rather that precision was the appropriate way to evaluate our claims. Overall, we view the present findings as a remarkably successful first step toward an integrative model.

### Opportunities for improvement

While LAG-1 does a fine job fitting the important qualitative findings, which are summarized in Table 4, there is significant room for improvement in getting the magnitudes correct. While many of the findings might be improved by tweaking one or more of the numerous fixed parameters that influence model timings, there are two major areas in particular where LAG-1 diverges from the human data in ways that seem more fundamental. First, the model does not show enough feature neglect (i.e., inattention to critical features), and second, the variability on many measures, like fixation duration and probability of fixating an irrelevant feature, is too small.

**Table 4:** Summary of simulation results.

Measure	Simulation 1	Simulation 2
Learning curves	Appropriate scale and reductions (overall).	N/A
Learning transfer	N/A	Appropriate generalization patterns.
Fixation counts	Appropriate scale and reductions (overall).	Appropriate scale and reductions (by feature).
Fixation durations	Appropriate average intercept/change, within-individual variability needs improvement.	N/A
Fixation orders	Appropriate average intercept/change, within-individual variability needs improvement.	N/A
Feature fixation proportions	N/A	Appropriate average intercept/change, within-individual variability needs improvement.
Reaction times	Appropriate average intercept/change, within-individual variability needs improvement.	Appropriate average intercept/change, within-individual variability needs improvement.
Feedback processing time	Appropriate average intercept/change, within-individual variability needs improvement.	N/A

N/A specifies findings not modelled for that simulation.

Feature neglect is most obvious in Fig 10: LAG-1 fixates too many features. At the start of the experiment, humans ignore a stimulus feature about 25% of the time. Intuitively, this seems strange. After all, how can one learn which features are predictive of the category if they are not viewed? In addition to causing difficulty for LAG-1, this finding also contradicts the assumptions made by models such as ALCOVE, which spreads attention evenly across all features at the beginning of an experiment [16]. It is not only in the [10] data that we find this pattern however, we also see this in the [25] data which had a different number of stimulus features. Indeed, [26] reports the probability of ignoring irrelevant information from 10 experiments, and the range of feature neglect seen in the first block of the experiment was between 10% and 40%. One possibility is that this is a function of missing fixations due to eye tracker error; but two facts mitigate against this idea. First, we ran simulations using LAG-1’s gaze data wherein we dropped gaze points (because we used LAG-1’s data, there were no naturally missing values) in the same proportion, and with the same sequence length, as in humans, to see if the missing data would cause reduced fixations, and thus feature neglect. It did not. In fact, the overall number of fixations in the experiment increased. Second, we reanalysed the human data from the [25] study, using only trials in which we had 100% of the gaze data and found there was still significant feature neglect. [10] did note that this may be indicative of participants using rule-based strategies initially, but they ultimately conclude that there’s likely a mixture of strategies at work. Without further study, it is difficult to say for sure, but we note that there is at least some feature neglect even in the case of mouse driven interfaces such as used in the second experiment of [25]. Regardless, LAG-1 does not show significant feature neglect, and this fact influences the model’s fit to several other measures. For instance, the within-trial fixation probabilities do not quite match the human data (Fig 6), primarily because it looks too often at irrelevant features. Overall, LAG-1 does well with the learning-related changes in fixation probabilities, but does not capture all of their overall levels.

**Fig 13:**
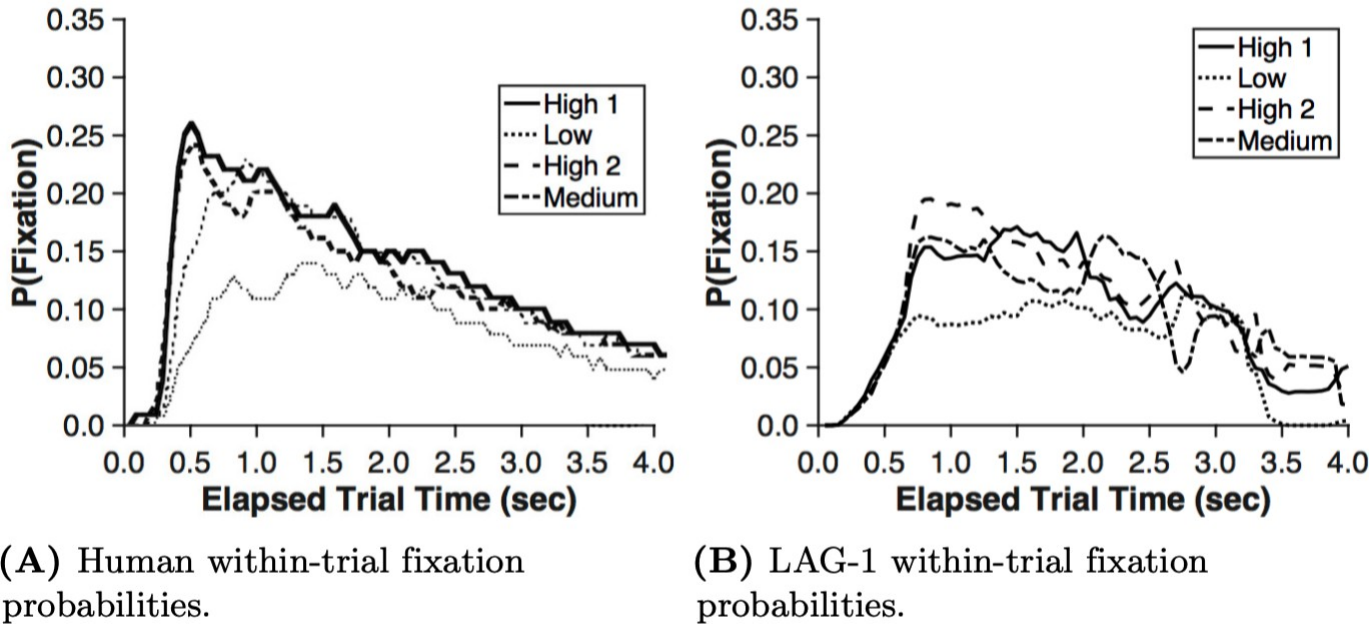
Saccadic timing neurons. Two examples of the contemporaneous activity of the three neurons of the Saccade Timing System, at two different points in learning. A) Before the model has made any strong feature-category associations, different stimulus features are equally interesting to it. Because there is little preference for particular features at this point, competition to determine the next saccade target takes longer to resolve than later in the experiment, extending the fixation duration in time. In these cases, the Gaze Change Neuron (blue) eventually surpasses the Fixation Neuron (red), just prior to the phasic spike in activity of the Saccade Initiation Neuron (black) which initiates a saccade. B) After the model has established stronger associations between features and categories, the knowledge that particular feature locations contain useful information, speeds targeting by boosting priority at these locations in the Spatial Attention Field. This is reflected in the modified timing relationships of the three neurons, where the Gaze Change Neuron does not reach the same level as the Fixation Neuron before the Saccade Initiation Neuron spikes. As a result, fixation durations are 30–40 ms faster than at the start of the experiment.

The other area where LAG-1 could provide better fits is in predicting the variability across, and within, individuals. This is most noticeable in our attempt to account for fixation durations (Fig 5B), but can also be seen to a lesser extent in measures like the probability of fixating an irrelevant feature (Fig 5D). Given the fact that almost every equation in the model has a noise parameter, it is sensible to ask if we might achieve the appropriate variability by simply increasing the noise on some of these. Possibly. Changing the variability, by increasing noise in one of the components of the model, can have a large impact—one that reverberates throughout all the dynamics of the system. In Fig 13 one can see that the neurons of the Saccade Timing System have very little noise. As an example, imagine that noise was added to the neurons of this system. This could increase the variability in fixation durations, but it would also influence ongoing Hebbian learning between features and categories; a fixation that lasts 800ms would end up having 4 times greater category association than would a 200ms fixation, and that, in turn can increase the variability in the speed of learning. Time sensitive activity is known to modify fundamental motor control over the eye [59]: a fact that causes additional challenges for modelling efforts. We felt it was more important, as a first step, to reproduce the primary attentional learning findings shown in Table 1, than to capture the variablity more generally, though we strongly agree with the idea that variabilty is important to fit [119].

### Application of LAG-1 to other category learning situations

There are many variations of basic category learning experiments, and we have fit only a small subset of them. The studies we fit had binary valued stimuli, spatial separation of the stimulus features, self-paced responses, self-paced feedback viewing, and the re-presentation of the stimulus during feedback. But, what if they did not? Can LAG-1 handle task variants?

LAG-1 can easily handle any temporal manipulations—for example, adding a specific stimulus presentation duration rather than the self-paced presentation—with no modifications. Manipulations like changing the feedback presentation duration, presenting each stimulus feature for a different amount of time, presenting stimulus features in different orders can also be easily be implemented. Note that many of these experimental manipulations seem likely to influence the performance of the model, and are thus predictions; in LAG-1, as a theory, time matters for many things. For example, if one were to run LAG-1 through two conditions, one with a short feedback phase, and one with a long feedback phase, LAG-1 would predict that the long feedback phase would lead to stronger associations between active features and categories because of the time dependence of Hebbian learning. We note that this prediction about a temporal manipulation falls outside the scope of what extant category learning models can address because they are generally not modelling cognitive processes in time, and we also note that this prediction has some empirical support [30, 37].

LAG-1 can also deal with spatial manipulations with little to no modifications to the core model. LAG-1 can accommodate different spatial configurations, and make predictions about how this will influence measures like fixation order, reaction time, and even learning. Again, the configuration of the stimulus seems very likely to influence patterns of attention and gaze, and in turn, performance and learning. Imagine an experiment in which, in one condition the features are spread out in a triangle shape, and another condition in which two features are near each other on the left, and the other feature is on the right. This spatial configuration would likely lead to faster reaction times, because they require less time to inspect the features. The activation of two features next to each other (if they are sufficiently close) could join to create a larger peak on the visual field, and thus be more likely to attract attention. What if we compared a situation where the two features on the left were relevant for the correct classification of the stimulus and the feature on the right was irrelevant to one in which the relevant features were separated? It seems plausible that having the relevant features next to each other (and fixated first) might lead to a bit faster learning in that condition, which is to say that the interaction of learning and attention is influenced by the spatial factors that LAG-1 incorporates.

Other kinds of changes to the experiment can be implemented with varying difficulty. Removing the visual feedback indicator (to simulate a sound indicating accuracy, for instance) would be very simple to implement. We would omit the feedback button as input to the Visual Field. The correct category node can still be activated by feedback and the associations between features and categories can change as usual. This requires a change to the experiment (changing what is displayed to the model) but no change to the core model. More substantial changes would be required for something like comparing information sampling using eye movements to information sampling using hand movements. Manual information sampling (e.g., using a computer mouse to click on features to reveal them) is less variable, and more efficient, though slower, and shows similar changes throughout learning [25]. To the extent that we think of information sampling and the allocation of attention using the eyes to be broader phenomena (e.g. “active sampling”, [120]), additional mechanisms that implement manual selection of information—such as clicking of menus in a computer program—would be needed. Accepting stimuli with continuous dimensions rather than binary valued features is something LAG-1 is already coded to do, but were not used in the eye tracking experiments we simulate and added unnecessary complexity for the current purposes, so we omitted it here. Having more complex visual processing sufficient to categorize natural images would require more extensive modification. Work by [51] is a good example of how a DNFT-based model might approach this challenge.

### Application of LAG-1 to a broader range of experimental situations

Our goal in creating LAG-1 was to build and test a model that can capture the interactions between learning, attention, and gaze. Category learning is a useful task in which to test this integration: participants learn categories and attend to features of varying relevance; further, both response behaviours and gaze behaviours change in concert throughout the experiment. Only a model that specifies how learning is related to the allocation of gaze, and vice versa, can illuminate this co-evolution. A wide range of experimental paradigms within the broader realm of visual cognition depend on these systems, however, and in this section we will provide a rough sketch of how LAG-1 might be expanded in a variety of ways to address to such situations.

#### Bottom-up salience

In category learning tasks there is a clear goal: participants are choosing to fixate particular locations to get information that helps them choose the correct category. The emphasis of the task is on learning to direct top-down attention toward task relevant features, the locations of which are well known. The free-viewing of scenes, in contrast, is a function of bottom-up salience. Though LAG-1 does not include many aspects of bottom-up attention–in category learning experiments stimulus differences in bottom-up salience are controlled for by the choice of features and counterbalancing—nevertheless, adding the influence of bottom-up salience into the model is straightforward.

**Fig 14:**
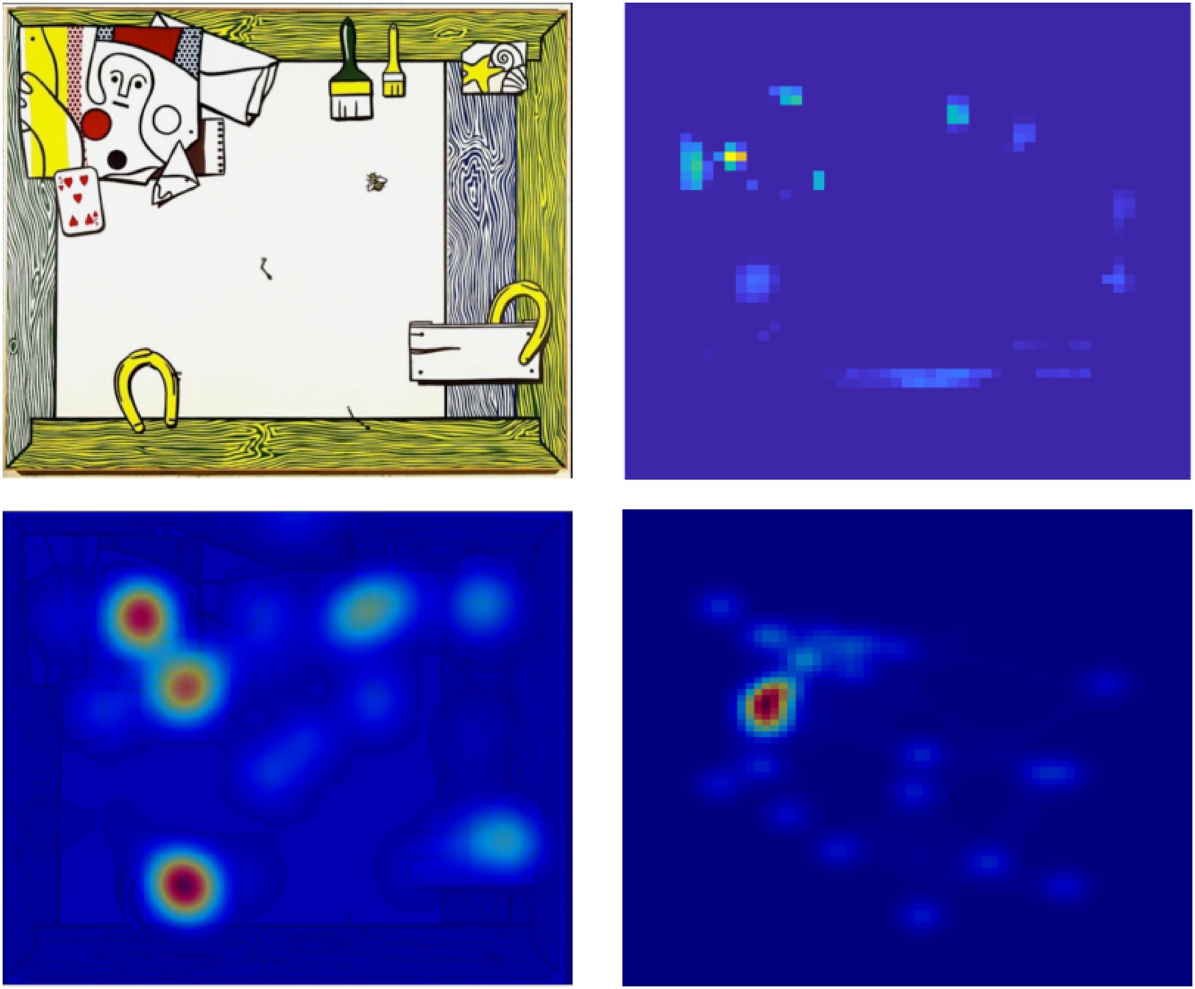
Salience map representation of model during free-viewing. Top left) “Things on the Wall” by Roy Lichtenstein. Top right) a salience map generated by three boundary detection iterations at different spatial scales, integrated across the dimensions of: color, orientation, and intensity. Bottom left) Fixation heat map from a typical human subject. Bottom right) Fixation heatmap from one trial of LAG-1.

To demonstrate this we added an additional input to the Visual Field (implemented as an additional layer) that acts as a preprocessed salience map. To handle the wider range of field inputs, we also changed the function that chooses saccadic targets from a greedy, maximum activation based selection, to a probabilistic choice, like was used for the category decision. This allowed the model to fixate a broader range of locations. These small changes to the model allowed us to use an image as a stimulus. To do this, the image must be converted to a salience map that, when input to the Visual Field, can drive fixations to the most salient (in the bottom-up sense) areas of the image. Though we left the category learning system in place, there is no input to the Feature Detection Neurons, and so no top-down attention is generated. Fig 14 shows the original image (Roy Lichtenstein’s 1973 painting: “Things on the Wall”), the salience map it produced, a heat map of a typical human free-viewing the painting, and a heat map of LAG-1 fixations viewing the image. Note that this implementation of the model predicts bottom-up salience influences the Visual Field earlier than top-down forces because the recurrent loop through the whole system takes extra time. Indeed, short latency saccades are associated with bottom-up salience and longer latency saccades are more influenced by behavioural goals [121].

For expediency, in this demonstration we created the salience map with a common Matlab package [122]. For example, we could implement the components of the salience map (i.e., color, orientation, intensity) as layers of the Visual Field, and calculate the salience from that activation. Abrupt onset and motion are also known to act as a source of salience (e.g., [123, 124]). Both of these factors might be implemented using large changes in activation at a particular location of the Visual Field to boost attention (see [45] for a DFNT-based approach to change detection). It is intriguing to consider that a system with robust, top-down attention may actually *require* a strong abrupt onset salience mechanism to function effectively because significant top-down attentional boosting of known, or expected features and locations, might otherwise make fixating new, unanticipated stimuli too improbable and slow.

#### Uncertainty in feature location

In category learning the main focus is on the learning the connection between features and categories. In other situations the location of the information sought is unknown. One of the most iconic visual cognition tasks is visual search [125]. In visual search the categories used in the task (target and distractor) are simple and explained from the beginning; no learning is necessary, or in most experiments helpful or desired. Another difference is that while feature locations are static and known to learners in category learning tasks, feature locations are unknown in visual search. While the specifics of what is known and what needs to be learned differ between visual search and category learning, there is a general level at which the tasks are similar: one is presented an image, then one looks around for information in the image, and then makes a response. LAG-1 performs search in a guided fashion (e.g., [126]). It has a selective processing stream—the Learning system which only processes information about features that are fixated—but, it also has a non-selective one: the Visuospatial System, which processes information about basic visual properties (e.g. color) and their locations. Scene statistics and other bottom-up influences on salience are not currently part of the model, but could be incorporated in the same way as a saliency map is used in the free-viewing section above.

The most straightforward way of doing visual search in LAG-1 is to take “target” and “distractor” to be categorical designations (in place of category A and B). Features that indicate target and distractor categories are set in advance (i.e., hard coding the difference into the information gain matrix in this demonstration). In the model there are two recurrent pathways from the category level back into the Visuospatial System. One goes to the locations of particular features on the Spatial Attention Field. In category learning, this linkage between features and their location in space makes sense because participants know the feature locations—which do not move—from the instructions. In a visual search task, however, using this to guide search seems unrealistic because the participant has no knowledge of how particular features connect to particular locations on the Spatial Attention Field. To address this LAG-1 has a second connection: this one links back to the various layers of the Visual Field and can thus produce low-level feature-based attention [86]. Search in LAG-1 is thus guided using this second recurrent pathway. Knowledge about the target one is looking for recurrently boosts activation in the corresponding layers in the Visual Field via gain on the appropriate Feature Expectation Neurons. This second pathway raises the activation of the locations of those features and thus the chance that they will be selected as a visual target from the resultant boost on the Saccade Motor Field, in other words, guided search.

**Fig 15:**
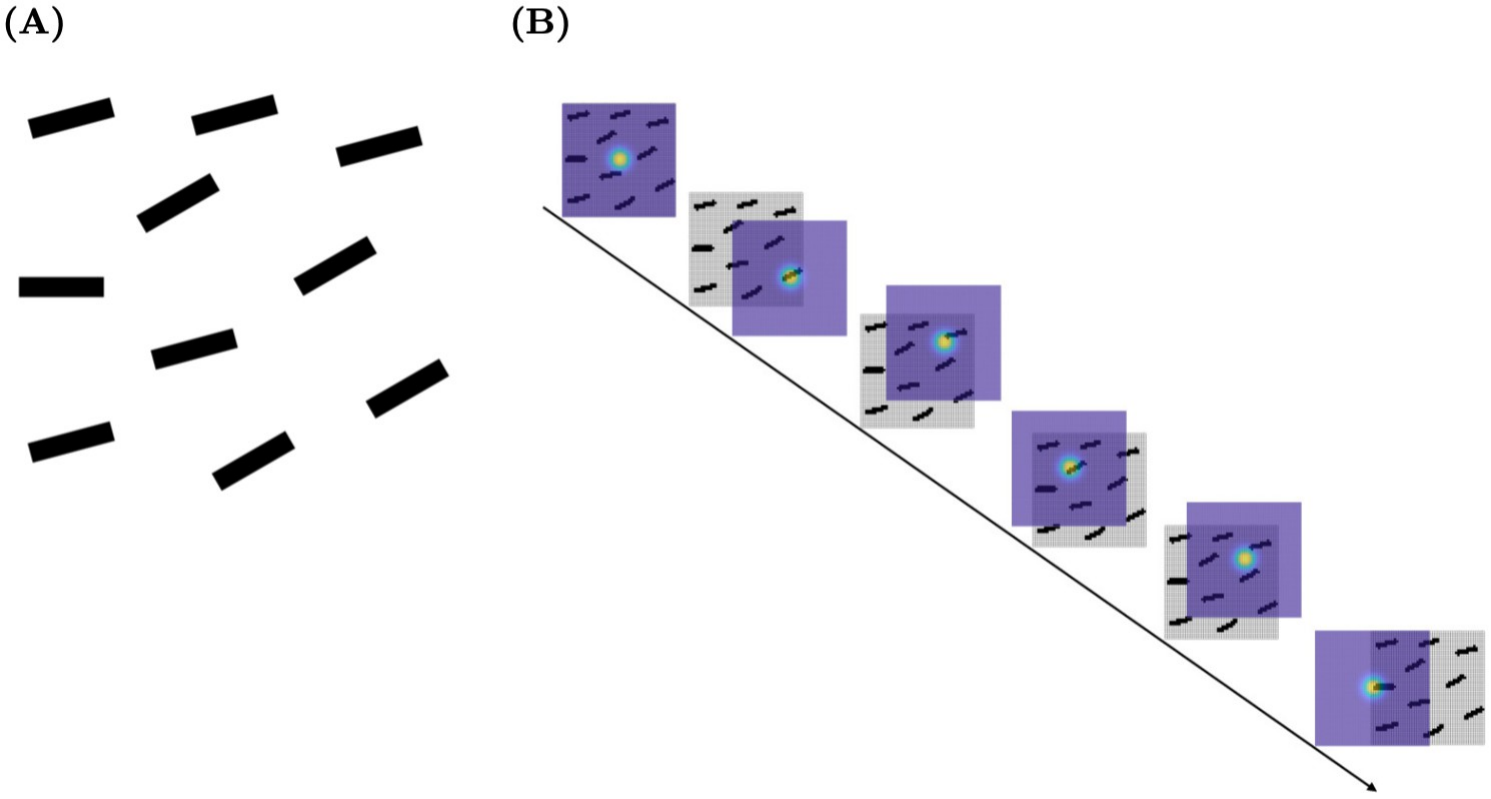
LAG-1 applied to an example visual search problem. A) Visual Field input array recreated from [53]. B) An example trial of LAG-1’s visual search scan path. Each frame is centered at the successive fixation location until the target is foveated.

Consider a task in which participants are searching for a vertical line amongst lines of 15°. In simulating such a task, as we have done with LAG-1 and shown in Fig 15, the feature-specific layers of LAG-1’s Visual Field would code orientations of differing degrees (instead of the six different colors we used in the category learning simulations, the six layers used in Simulation 1 can code six different orientations. Top-down signals boost the Feature Expectation Neurons (or field, in this case) associated with the target orientation, leading to increased activation on the corresponding slice of the 3D Visual Field. This boost causes an increased likelihood of an eye movement to that location, relative to unboosted locations, and thus more efficient search. In this implementation of visual search, LAG-1 will look around from item to item, and when an item is fixated the Feature Detection Neurons are activated, which in turn activates the associated category nodes (target or distractor). The model will only stop when the model fixates the target pushing the Category Neuron for target to its threshold, or until the Trial Impatience has driven a click decision (e.g. on target absent trials). LAG-1 does not search in parallel (though again, its choice of target locations is biased toward the boosted location): it must confirm that a particular object is the target by fixating the target. This simple version of visual search, again, implemented with only minor changes from the category learning implementation, undoubtedly will produce the common findings of serial search slopes and RT differences between target present and target absent displays. Search will also be less efficient in the case of search for a conjunction of features: distractor locations that share properties with the target would also receive a boost, decreasing the relative advantage of target over distractor locations, and thus decreasing the target’s advantage. Overall, what LAG-1 would predict is that search is more or less efficient depending on the degree of overlap between target and distractor properties [127].

Foundational findings in visual search include accuracy and response time differences as the items of the search array differ by category, such as letters and colours, how they flank one another, and their variability. Several of these seem amenable to simulation by a visual search version of LAG-1. Knowledge effects, such as the influence of target and distractor similarity, fall out of the category learning system [128] as described above, as presumably would category effects [129]. Flanking, wherein search is slowed if distractor orientations are on both sides of the target orientation, also seems to have a relatively straightforward explanation that derives from the spatial dynamics of neural fields. In a more generalized version of our visual search extension, input from a small line length of 15° could more strongly activate neurons tuned to that orientation and weakly activate ones of nearby frequencies leading to a center surround bump on the field leading to a flanking effect. In LAG-1, this finding would make sense in that activation of a target could slightly activate orientations that are associated with distractors due to overlap (again, we are imagining a 3d visual field—with *x* and *y* as spatial dimensions and *z* is orientation—and a Feature Detection Field that codes orientation continuously). If distractors flank both sides, there is more overlap, and therefore more competition between target and distractor nodes. This competition would straightforwardly weaken the relative advantage of the target within the recurrent channel. This account makes the assumption that the orientations are close enough that there is some overlap in the activation bumps on the field. The model thus makes the prediction that the flanker effect would be strong in cases where the differences between targets and distractors are small, but may be weak, or non-existent if distractors are very different than targets, and thus have no overlap on the field (see Fig 19 in S1 Appendix).

Some findings do not seem to have a natural explanation given the current structure of LAG-1. The finding that search is slowed if there is more variation in distractors, even if that variation is farther from the target, (a 30° target among 15° distractors is found faster than a 30° target among both 15° and 0° distractors) has no obvious explanation for example [53, 130]. We can also see no easy method of accounting for search asymmetries such as the result that a 0° target among 15° distractors is easier to find than the reverse. More substantial modifications to the model seem necessary to account for such phenomena. By design, in most versions of visual search, learning mechanisms do not aid performance. However, in contextual cueing a display in a visual search task is repeated multiple times, and participants in these experiments show improved search times for these repeated displays [131]. LAG-1 has no method of producing this effect in its present form; learning in the model associates features with categories, but the location of the features is presumed to be known (consistent with the category learning tasks to which it is applied in the current work). [132] simulated contextual cueing using a simple two layer network in which distractor locations are associated with the target location upon each presentation of the display. This model restricts learning to those distractors that are nearby the target. Adding a similar mechanism to LAG-1 would be relatively straightforward. An additional field—a Visual Memory Field—could be linked via connections to the Visual Field, such that when the target is found (or more broadly, when reinforcement is presented), connections are strengthened between the Visual Field, and the Visual Memory Field around the area of fixation. Such a modification would more or less embed the [132] model into the larger LAG-1 framework.

#### Adding broader feature extraction to address covert attending

In the current work, one focus of ours has been on modeling overt attention using eye tracking data. To account for these human behavioural phenomena we have proposed, in simple terms, the processes that lead the eyes to orient toward features for category learning, and in doing so, we have also specified how features and feature locations receive processing advantages which give them more influence in the complex, interactive processes enabled by the system as a whole. These processing advantages, because they are not directly noticeable, would count as one form of covert attention.

Though the distinction between, and indeed the relationship between, overt and covert attention is captured in the model, there is room for extensions that might account for a larger set of data, but which would not (likely) hamper the performance of the model in category learning tasks. In the current work, we have assumed that eye movements result from a shift of attention, and the uptake of the new information, centered on the Visual Field, is passed on with full clarity to the Learning System. This assumption does not hold for the broader world of visual cognition, and likely not even for some aspects of simple category learning tasks. For example, visual search tasks can be created that allow people to perform them while maintaining fixation to a central point, that is, without eye movements [133]. Further, even in some category learning studies conducted in our lab, we have noticed that certain participants, after hundreds of training trials, continue to perform perfectly, but without fixating their gaze all the features necessary for correct classification. This suggests two things: that information in peripheral vision may be used in object recognition, and that learning seems to influence, or is at least related to, people’s ability to do this. The current implementation of LAG-1 cannot account for these findings.

One way of augmenting LAG-1 that might better accord with these findings is to include a separate eye movement field coding a distinct force component [81]. Normally, eye movement signals center the fovea over the target, providing high acuity information to the category learning system. But what happens if an eye movement is suppressed? Top-down signals could prevent the gaze shift by inhibition of the field coding force, yet still allow the Saccade Motor Field to indicate a target, and for the Spatial Attention Field to shift to another location on the Visual Field. This would produce covert attentional shifts, as the eye would be centered on one location, while attention—in the form of the Spatial Attention and Saccade Motor Fields—would be focused somewhere else. In cases where the Spatial Attention Field and the fovea were not over the same location, the information released to the category learning system would depend on the visual acuity at the focus of attention. This extension would describe overt and covert attentional systems in distinct terms, and allow the model to be applied to tasks for which covert and overt systems are strongly dissociated. In the case of covert attention during category learning, experience increases the ability of features to activate categories, eventually allowing even weak peripheral feature values to be strong enough to lead to a classification, and thus, no need for an additional fixation.

One limitation of the current version of LAG-1 is that it requires some calibration to handle different experimental situations. To some extent this is true for most models—in that changing the number of categories, or the number of features requires a new structure—but because of the dynamics in LAG-1, additional calibration is required. In Simulation 2, for example, the input comprised four stimulus dimensions rather than the three used in Simulation 1. This difference changes the competitive dynamics on the Spatial Attention Field, in that the height of a typical bump on the field is lower when there are more features, due to their being more global inhibition on the field. Smaller peaks change the time to reach saccadic thresholds, and influence the highly interactive model components in many other ways. Also, there were only two categories in Simulation 2 whereas there were four categories in Simulation 1. This change modifies the total energy of the Feature Detection, Category, and Feature Expectation layers, and, in turn, leads to different behaviours. It would be better if LAG-1 did not require this kind of tuning as clearly human learners do not. The issue is complex, however, and while it may be easy to take a negative view of LAG-1 on this matter, it is useful to remember that there are many examples of sensitivity adjustments in humans that take noticeable amounts of time to resolve. The visual system, for instance, cannot process extremely bright light, such as the noonday sun, and adaptation to low light conditions takes time: full dark-adaptation can take 30 minutes, and depends on complex adjustments, not just in the visual cortex, but even in the chemistry of the retina [134]. Also, the adult human visual system has already undergone a long process of self-organization in tuning appropriate expectations based on the demands of the physical environment [135]. We have no such developmental process in LAG-1. We have however, tried to take some tentative steps in thinking about suitable homeostatic processes (e.g., [136]). Developing more general mechanisms for self-calibration will be an important target for future research.

In summary, LAG-1 has broad applicability to the diverse tasks and phenomena within visual cognition. We have taken some first steps here, having implemented a few changes that allow for application of LAG-1 to free-viewing and simple visual search tasks, but more work needs to be done to broaden the model’s account to include contextual cueing and covert attention. That such effects can be considered modelling targets gives credence to the idea that integrating attentional, fixational, and associative processes in a single model is an important goal.

### Contributions and conclusion

While we have discussed at length the details of the model, the methods used in the simulations and the fits to the data, it is important to emphasize that, at the broadest level, the theme of the present work is *integration*. It proposes connections between different neurofunctional systems, and between different empirical phenomena operating at different timescales. Alan Newell identified four timescales of cognition, emphasizing that biological, cognitive, rational, and social phenomena, are not just different classes of phenomena, but that they occur at different speeds [19]. One important aim, then, is to understand how biological phenomena that are rapid, give rise to slower cognitive phenomena (such as a classification decision), and in turn give rise to still slower phenomena (such as category learning). LAG-1 gives just such an account; one that spans most of Newell’s temporal bands of cognition. At the finest scale the model resolves changes in neural activation with short timesteps (∼10^−2^ seconds). These neural changes build over time to drive saccadic eye movements (∼10^−1^ seconds), which are in turn ordered according to the expected information gain derived from Hebbian learning at the trial level (∼10^0^ seconds), shaping the perception of abstract categories learned on the order of ∼10^1^ seconds. Finally, as associations strengthen and decline over trials, the model describes a continuous relationship between average learning curves and average attentional learning (∼10^3^ seconds).

LAG-1 is not an attempt to be a better model of category learning, or of oculomotor control, or of attention. Instead, it is a step toward building bridges *between* established work in these areas by providing a theory of how they might interact. As a result of the integrative approach to the construction of the model, LAG-1 can be applied to more than just category learning performance (e.g. accuracy, transfer probabilities). LAG-1 can simultaneously account for measures that have only occasionally been modelled (e.g. reaction time, probability of fixating features), and further, to measures that have never been directly modelled (e.g. time spent viewing feedback). We fit learning phenomena, and we fit attention phenomena, but most importantly, we account for their interactions (e.g. learning-related changes in fixation durations). We know of no related model that has shown good qualitative fits to as many different empirical findings as LAG-1.

Even just the existence of a dynamic theory of attention, learning and gaze provides theoretical motivation for novel experiments with temporal and spatial manipulations that could reveal a whole host of psychologically interesting phenomena related to learning and attention. Not only can it motivate new kinds of thinking and research about attention and learning in action, but it can also be constrained by, and improved based on, findings from a wide variety of investigations in the cognitive sciences.

## Supporting information

S1 Appendix Primer to dynamic neural field theory [16, 40, 51, 66, 69, 117, 137–149]. (PDF)

S2 Appendix Companion equations for the formal description of LAG-1 and neurophysiologial context [16, 99, 108, 150, 151]. (PDF)

S3 Appendix Fitting procedure [10]. (PDF)

S4 Appendix Supplementary equation parameters. (PDF)

S5 Appendix Parameter tables and best fits. (PDF)

S6 Appendix Individual fit visualizations. (PDF)
