# Supplementary material for: LAG-1: A dynamic, integrative model of learning, attention, and gaze
Source: PLoS One. 2022 Mar 17;17(3):e0259511. doi: 10.1371/journal.pone.0259511 (PMC8929614; doi:10.1371/journal.pone.0259511)
Supplement: S1 Appendix — (PDF) [file pone.0259511.s001.pdf]

S1 Appendix. Primer to dynamic neural field theory.

This section explains the differential equations that describe the time evolution of the dynamical variables of the model. It is our intention here to make the modelling more accessible to readers unfamiliar with dynamical systems.

Neurally inspired computational cognitive models have been used to support research at a variety of different ontological levels including: transmitter systems [137], single neuron computation [117], error-driven learning (e.g. [16, 138]) as well as whole-brain models [139]. By writing differential equations for the activations of the units in these models, time-dependent behaviours can be modelled. This is done by stating how the activations of the units in the system change through time in the presence of some input (which may also change though time).

In LAG-1 we use two main types of processing units: single neurons and fields. While the concept of a neuron in a cognitive model may be familiar, the use of fields is less common. One way to think of a field is as an organized collection of neurons that work together to represent some variables or relationships of interest. LAG-1 uses neural fields primarily to represent spatial dimensions, but fields can also be used to represent other metric dimensions such as color, size or aspect ratio [51]. Individual neurons have preferential responses to particular values along those dimensions. The emergent activity in a population can be thought of as a localized bump of activation in a space that monotonically orders the individual neurons by their preferences. In other words, the location of the bump on the field is what is representing the information. Because the representations are distributed across many neurons, this method of interpretation is referred to as population coding [140, 141, 142]. Neural population codes have been reconstructed throughout the brain. Such population codes contribute to our fundamental understanding of parietal [69], temporal [66], striate [143] and mid-brain [144] regions to name a few—all of which are relevant to the functioning of LAG-1.

Single neuron equations

We now look at the mathematics that specifies single neuron activation in LAG-1. An individual neuron's change in activity across an interval of time is described by the equation:

$$\dot{u}(t) = -u(t) + h + S(t) \tag{1}$$

In Equation 1, $\dot{u}(t)$ (also written as du/dt) defines the rate of change in activation of a neuron u, at a point in time, t. Over a short interval of time, δt, the amount of change in u is approximately $\dot{u}(t)\delta t$. The parameter h is a constant, indicating a resting level, or baseline firing rate of the neuron u. Exogenous input to u at time t is represented by the specific input term $S(t)$. When there is no input to the model, $S(t) = 0$, the rate of change of u will also be zero so long as $u(t) = h$, i.e. a neuron at its resting level will stay at its resting level if there is no external input.

If the activation of u is not at its resting level, and no other input is provided, then the value of $u(t)$ will converge exponentially toward h as a function of time. If $S(t)$ changes from 0, it will displace the activation from u from its resting level h (either up or down). In Figure S1, the behaviour of such just such a neuron-like variable is depicted, given some non-zero input for a period of time, i.e. $S(t) = 10$ for $50 \leq t \leq 100$, and $S(t) = 0$ otherwise. As can be seen, the neuron's activation stays at its resting level, -5, then jumps up to the sum of the input and the resting level $((10) + (-5) = 5)$. It maintains this higher activation until the input is turned off, at which point the neuron's activation immediately falls back down to its resting level.

Implicit in Equation 2 is a time scale for its changes in activation. The time scale in Figure S1A is on the order of one unit of time, meaning that the transition in activation

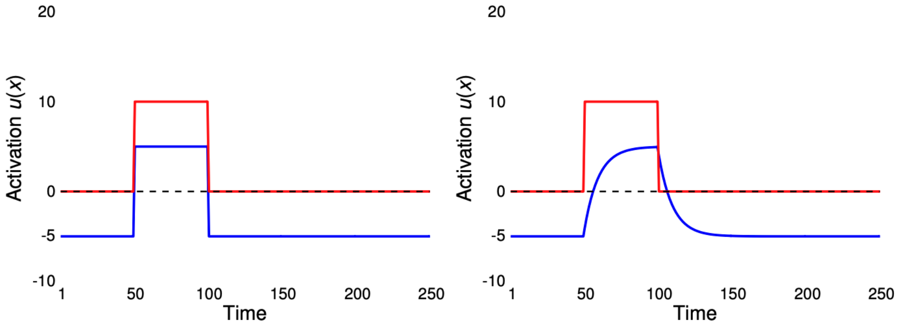

(A) Timescale obscured. **(B)** Timescale expanded.

Figure 16. Characteristic timescales of activation. Activity of the neuron u plotted in blue and input to this neuron plotted in red. A) Activation of the neuron versus time when $\tau = 1$. B) Activation of the neuron versus time when $\tau = 10$.

is not clearly visible in this plot. We need to introduce a parameter to control this time scale in order to be able to match it to human behaviour. The time scaling parameter to do this is τ, included on the left-hand side of Equation 1, as shown in Equation 2.

$$\tau \dot{u}(t) = -u(t) + h + S(t) \tag{2}$$

The τ parameter dictates how long it takes for a change in input to impact on the variable. In this definition, larger values of τ mean that the system evolves more slowly. This can be seen by dividing the equation by τ and observing that the larger τ is, the less the rate of change of u is. Figure S1B shows the difference in activation over time, as scaled by τ.

Numerical simulation of the equations

The dynamics produced by Equation 2 are simple enough that we can write down an exact solution for $u(t)$. In more sophisticated models however, this is usually not possible. Instead, we must obtain a sequence of approximate values of the variables over a sample of time points. To do this, a step length, δt, must be chosen that specifies the constant distance between the computed points in time. Starting with an initial activation value for $u(0)$, the approximate values at each point in time are obtained as $u(\delta t)$, $u(2\delta t)$, $u(3\delta t)$, and so forth. Choosing a smaller δt provides a more accurate solution, but is more computationally expensive, as the number of steps that must be taken to simulate over a time interval of length T is $T/\delta t$. A larger value for δt means that the solutions can be obtained faster, but risks being inaccurate. Given an initial value for $u(t)$, an approximation for its value at $u(t + \delta t)$ is given by:

$$u(t) + \frac{\delta t}{\tau}(-u(t) + h + S(t)) \tag{3}$$

Notice that the new value of u given by Expression 3 is equal to the value of u at time t plus δt times the derivative of u. A good rule of thumb in selecting δt is that the timesteps need to be smaller than the shortest time scale in the problem at hand. For LAG-1 we discretized time to just below what was needed for the model to make accurate saccadic eye movements: one unit of model time is 10 ms of real time when $\delta t = 1$. If a human participant tends to fixate stimulus features for 300ms then the model should be similarly fixating for 30 time steps.

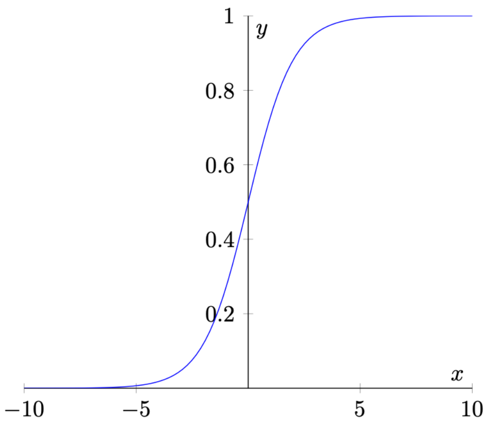

Figure 17. The sigmoid activation function. The sigmoid function defined in Equation 5. This function asymptotes to 0 and 1 as $x \to -\infty$ or $x \to +\infty$ respectively: $(f_{\beta=1,u_0=0})$.

Endogenous input and self sustaining activation

Recurrent connections can be added to systems like that defined in Equation 2. This can allow for a kind of memory.

$$\tau\dot{u}(t) = -u(t) + h + S(t) + cf(u(t)) \tag{4}$$

In Equation 4, a variable $f(u(t))$, representing input to the neuron based on its own level of activation, is added to Equation 2, scaled by a constant parameter c. This input consists of the activation of the unit passed through a non-linear sigmoid function f, which takes any real valued number as input and returns a value between 0 and 1 as output. This is a normal operation, that keeps activation bounded while also having some useful mathematical properties for complex learning. Figure 17 plots the behaviour of an example sigmoid function defined in Equation 5 below. The sigmoid function is used in many places in LAG-1 with different values of the parameters β, u_0.

$$f_{\beta,u_0}(u) = \frac{1}{1 + e^{-\beta(u-u_0)}} \tag{5}$$

The introduction of the recurrent term for u allows the possibility of two distinct equilibrium states. When $u(t)$ is low, the input from the $f(u(t))$ term will also be small and may not significantly influence the overall level of u. When $u(t)$ is high enough however, the input from $f(u(t))$ will abruptly increase because of the non-linear sigmoid transformation, resulting in yet larger values of $u(t)$, and furthering the tendency to maintain $u(t)$ at a higher level of activation. The low activation state and the high activation states are referred to as stable fixed points and can be considered as distinct states: "off" and "on". The system may be pushed from off to on (and vice versa) from external input or sometimes just with noise. Given a recurrent input like this, the system can effectively store a single bit of information. Adjusting the resting level h changes the relative ease with which the system may transition between these fixed points.

In Figure 18, the system defined in Equation 4 is simulated, such that the activation of the neuron starts from its resting level, and is subsequently excited by some exogenous input. While this is happening, the influence of the recurrent excitatory input is also increasing. When the exogenous input is removed, the output of $f(u(x,t))$ has reached a point where it alone can sustain the system's activation preventing it

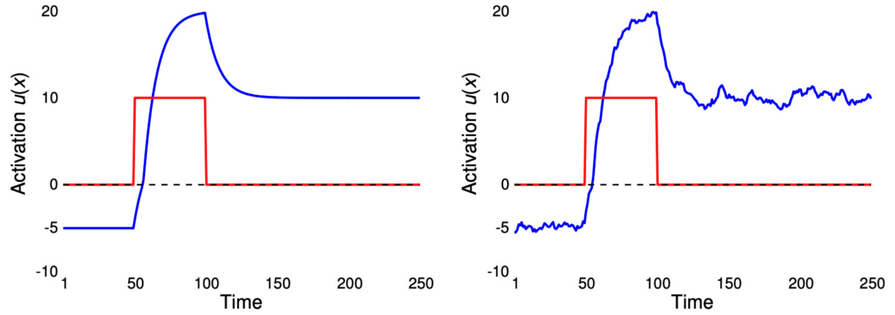

(A) Neuron with self-excitation. **(B)** Neuron with self-excitation and noise.

Figure 18. Dynamic variables with noise. A) Activation $u(t)$ (blue) and exogenous input $S(t)$ (red) as a function of time, for the system described in Eq. 2. B) The same system but with a small noise term added.

from falling back down to its resting level. In LAG-1, the Feature Detection Neurons, among others, have this property, allowing them to retain information about stimulus features as they are viewed over the course of a trial, even when the model has stopped looking at it [40, 145, 146]. Descriptions of neural information processing with these kinds of dynamics can be traced at least back to Hebb's fundamental hypotheses about the cell assembly and its relation to eye movements [147].

Noise Part 1

Variability in neural signaling is an inherent aspect of real-world neural networks [148]. To model noise, we can build on the systems defined earlier by incorporating a noise term, $\zeta(t)$.

$$\tau \dot{u}(t) = -u(t) + h + S(t) + cf(u(t)) + \zeta(t) \tag{6}$$

In Equation 6, $\zeta(t)$ represents Gaussian white noise: that is, noise having equal amplitude over all frequencies (scaled by $\tau^{1/2}$ for reasons that will be explained in the next section). Figure 18 shows the effect of noise added to the same system simulated in Figure 16. Noise introduces a certain amount of behavioural stochasticity by altering the time it takes for action thresholds to be met, for example, in choosing the next feature to look at or the moment to make a category decision.

One-dimensional field equations

The dynamics so far described for a single idealized neuron can be extended to describe neural fields. The activation changes at locations along the field are calculated just like those of single neurons except that there are excitatory and inhibitory interactions with the other locations in the field to include. The differing spatiotemporal contributions of inhibitory and excitatory components of a neuron's receptive field, combine to create a Difference-of-Gaussians (DoG) shaped kernel, depicted in Figure 19.

The expression in Equation 7 for $\dot{u}(x,t)$ defines the rate of change of the activation u at a specific position x. The field equation incorporates the same kinds of inputs as the single neuron defined earlier, including: a linear decay term $-u(x,t)$, a resting level h, an exogenous input $S(x,t)$, and a noise term, $\zeta(x,t)$. The important conceptual difference is that the variable x does not refer to a particular neuron, but rather an abstract location in a feature space relevant to a task. The recurrent term $cf(u(x))$ earlier included in the single neuron equation is substituted in Equation 7 with the

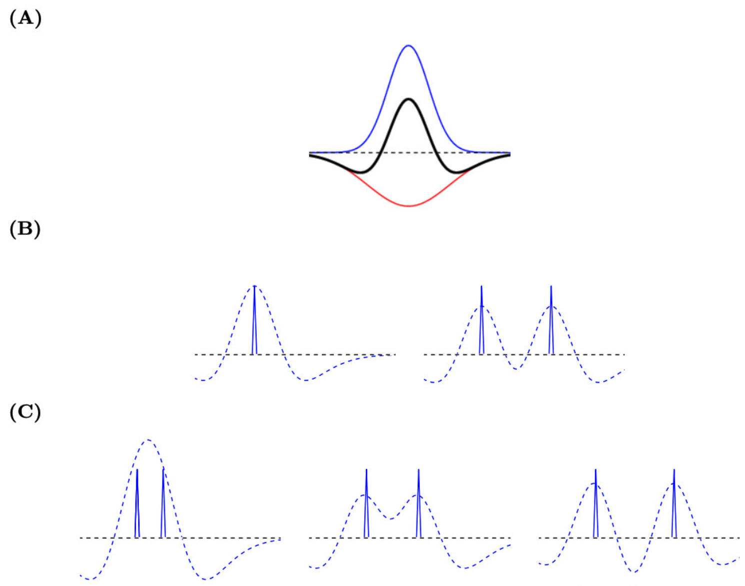

Figure 19. The making of a Difference of Gaussians (DoG) distribution in 1-dimension. A) Blue indicates excitatory input, red indicates inhibitory input and black represents their summation. B) The figure on the left shows a single specific input to a field as the solid blue line, and the result of convolution with the DoG kernel as the dashed blue line. The figure on the right shows the inhibitory effect that multiple inputs can exert on each other once convolved with this kernel. C) The degree of lateral excitation or inhibition between activations on the field depends on the distance between them. Locations close to one another, as on the left, can merge and increase in magnitude through local excitation. The activity at locations where the inhibitory component of the kernel is most pronounced, as in the middle figure, results in particularly reduced activation after convolution. Finally, more distant locations on the field show a more modest reduction of activity as the tails of the kernel become less and less negative, as shown in the rightmost figure.

convolution of a kernel, w, with the sigmoid of u, $f[u]$. This term incorporates the effects of excitation and inhibition on $u(x,t)$ from the other locations on the field, $u(x',t)$.

$$\begin{aligned}\tau\dot{u}(x,t) = &- u(x,t) + h + f(S(x,t)) + \zeta(x,t) \\ &+ \int w_u(x-x')f[u(x',t)]dx'\end{aligned} \tag{7}$$

The kernel is given by the difference of Gaussians (DoG) described in Equation 8.

$$w_u(x-x') = k_e \exp\left[\frac{-\left(x-x'\right)^2}{2{\sigma_e}^2}\right] - k_i \exp\left[\frac{-\left(x-x'\right)^2}{2{\sigma_i}^2}\right] \tag{8}$$

The kernel parameters, k_e and k_i, scale the magnitude of the excitatory and inhibitory contributions depicted in blue and red of Figure 19. The breadth of these excitatory and inhibitory contributions is parameterized by σ_e and σ_i to produce the final integrated weighting.

The net effect of the kernel convolution is similar to that of recurrent input in the single neuron case in that the excitatory distances of the kernel allow for two stable levels of activation on the field even in the absence of exogenous input if the recurrent

term is large enough. A single localized input will generate a bump of activity centered about that input after convolution with the kernel. When multiple points are already active on the field, kernel convolution has varying effects, dependent on the distance of the points relative to the shape of the kernel. Active locations near to one another combine to form a single larger peak. Active locations just a little further from one another may fall in the inhibitory-well of the kernel, yielding distinct but strongly dampened peaks. When the inputs are sufficiently distant, they avoid the well of strong inhibition they suppress each other to a smaller degree. The DoG kernel used within the model has some empirical support from studies of attention [149].

Two-dimensional field equations

Generalizing these equations to higher dimensions is straightforward with the difference being that a higher dimensional integral convolution is required to account for the lateral interactions within the population. This is computationally more demanding but adds little to the complexity of the mathematics. In Figure 20, the continuous version of a two dimensional kernel is depicted, alongside a less computationally intensive variant similar to what is used here, where distant locations of the field are considered to have no effect on each other.

$$\begin{aligned} \tau\dot{u}(x,y,t) = & -u + h_u + S^*(x,y,t) + \zeta(x,y,t) \\ & + \int\int w_u(x-x',y-y')u^*(x',y',t)dx'dy' \end{aligned} \tag{9}$$

The single dimensional field equation (Equation 7) is generalized to two dimensions in Equation 9.

We now can unpack one of the actual equations of the model, using the concepts we have introduced so far. Equation 11 defines the dynamics of the activity of the Visual Field. Remember that the experiment input has 3 dimensions: two spatial, (x, y), and one featural, (z). The expression for the Visual Field however is defined for only the two spatial dimensions. The value of the feature on the third dimension is used only when a location having a feature is actually fixated. The generic input previously referred to as S^* is now replaced with the actual terms for the external inputs to the field: in this case, the stimulus input $I_V(x, y, t)$, the acquired feature expectations $u^*_{\text{ft}_{\text{exp}}}(x, y, t)$ and the appearance of the feedback button $u^*_{\text{fb}_{\text{exp}}}(x, y, t)$.
The linear decay $-u_V(x, y, t)$ term, resting level h_{u_V}. and noise $v_\zeta(x, y, t)$ remain consistent with earlier definitions. The c parameters are simple scaling parameters and are discussed further in the formal model description in the next section.

Noise Part 2

There are two considerations when including noise in these equations that are easily overlooked: noise correlations and noise scaling. Noise across multiple spatial dimensions is correlated by the smoothing effect of the spatial kernel at every step through time whereas we set noise in time as being uncorrelated. This is implemented by starting with complete space-time white noise, having the property that the integral of it over an x-y-t cube of dimensions, δx, δy, δt, is normalized as a Gaussian random variable with mean zero and variance $\delta x \delta y \delta t$ (i.e. the volume of the box in $x - y - t$ space).

$$\zeta(x,y,t) = \tau^{1/2} \int\int w_\xi(x-x',y-y')\xi(x,y,t)dx'dy' \tag{10}$$

In Equation 10, the genuine white noise process, $\xi(x, y, t)$, covering the dimensions of simulation, is smoothed by convolution with the noise kernel w_ξ. The time scale term, $\tau^{1/2}$, ensures the correct scaling in time for different values of τ.

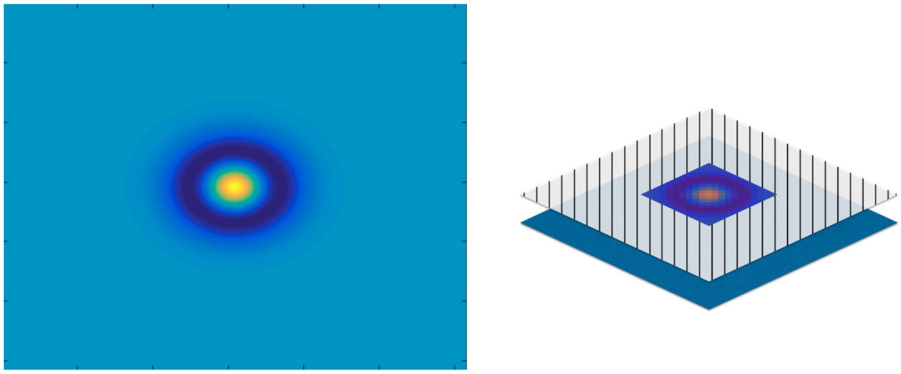

(A) Full, continuous kernel. **(B)** Reduced, discretized kernel.

Figure 20. Two dimensional difference of Gaussian kernels. A) Heat map of an idealized DoG in two dimensions. B) In practice this function is approximated by the sum of a localized function within a small square, and 0 outside of it.
