# Supplementary material for: LAG-1: A dynamic, integrative model of learning, attention, and gaze
Source: PLoS One. 2022 Mar 17;17(3):e0259511. doi: 10.1371/journal.pone.0259511 (PMC8929614; doi:10.1371/journal.pone.0259511)
Supplement: S2 Appendix — (PDF) [file pone.0259511.s002.pdf]

S2 Appendix. Companion equations for the formal description of LAG-1 and neurophysiologial context.

Visual Field

$$\begin{aligned}\tau\dot{u}_V(x,y,t) = & -u_V(x,y,t) + h_{V,1} \\ & + \sum_z I_V(x,y,z,t) \\ & + \int\int w_V(x-x',y-y',t)u^*_V(x',y',t)dx'dy' \\ & + c_{13}\sum_z u^*_{\text{ft}_{\text{exp}}}(x,y,z,t) \\ & - c_{14}\sum_z\int\int u^*_{\text{ft}_{\text{exp}}}(x',y',z,t)dx'dy' \\ & + u^*_{\text{fb}_{\text{exp}}}(t) + \zeta_v\end{aligned} \tag{11}$$

The data structure representing the raw stimulus, $I(x,y,z,t)$, specifies x,y,t locations in retintopic space at each moment in trial time, for feature values $j \in z$. The strength of the feature value input is mediated by the saccadic masking: $1 - c_3u^*_r(t)$, where $u_r(t)$ is the value of the Saccade Initiation Neuron (high values of u_r suppress visual input). During the feedback phase of a trial, the correct category is also presented to the field, represented here as an additional feature, $u_{\text{fb}_{\text{exp}}}(t)$ and modulated by a signaling function $\mathbb{1}_{\text{phase=feedback}}$ that indicates whether or not the feedback feature has been fixated.

$$\begin{aligned}I_V(x,y,z,t) = (1 - c_3u^*_r(t))\big(c_2I(x,y,z,t) \\ c_4\mathbb{1}_{\text{phase=feedback}}u^*_{\text{fb}_{\text{exp}}}(t)\big)\end{aligned} \tag{12}$$

Spatial Attention Field

$$\begin{aligned}\tau\dot{u}_A(x,y,t) = & -u_A(x,y,t) + h_{A,18} \\ & + \sum\nolimits_z c_{19}u^*_{\text{ft}_{\text{exp}}}(z,t)u^*_V(x,y,z,t) \\ & + c_{25}u^*_{\text{fb}_{\text{exp}}}(t) \\ & + c_{20}u^*_{\text{fix}}(t)\mathcal{G}_{\mathcal{A}}(F,\sigma_{22})\mathbb{1}_{\text{sacc}} \\ & - c_{21}u_{\text{ior}}(x,y,t) \\ & - c_{28}u^*_g(t)\mathcal{G}_A(F,\sigma_{22}) \\ & - c_{29}u^*_r(t)u^*_A(x,y,t) \\ & + c_{45}\mathbb{1}_{\text{sacc}}u_{A\leftarrow V}(x,y,t) \\ & + u^*_{A\leftarrow M}(x,y,t) \\ & + \zeta_A(x,y,t) \\ & + \int\int\big(w_{A,A}(x-x',y-y',t) \\ & \quad u^*_A(x',y',t)dx'dy'\big) \\ & - c_{38}\int\int u^*_A(x',y',t)dx'dy'\end{aligned} \tag{13}$$

On the Spatial Attention Field, defined in Equation 13, input from the Fixation Neuron, u_{x} (described below) maintains pressure to continue fixating the current feature. This

input is transformed from a scalar to a Gaussian with the same width as the fovea, $\mathcal{G}_A(F, \sigma_{22})$, centered at the extant locus of fixation, F. These fixation forces compete with a polynomial increase in pressure to look away, mediated by a Gaze Change Neuron, $u_g(t)$. A saccade not only reduces the level of input from the Visual Field, denoted by an indicator function $\mathbb{1}_{\text{sacc}}$, but also reduces the total energy of the Spatial Attention Field as a proportion of the activity of the Saccade Initiation Neuron, $u_r^*(t)$. Excitatory input from the Saccade Motor Field $u_{A \leftarrow M}(x, y, t)$ heightens attention at the location of a planned saccade just as observed experimentally [150]. Category-related biases for particular locations emerge from the continuous reentrant activity of Feature Detectors, Category Neurons, and Feature Expectation Neurons, as they project into the Spatial Attention and Visual Fields. Simple associative learning between Feature Detector Neurons (that implement visual working memory) and Category Neurons, is enough to effectively bind information obtained over a series of fixations, enabling a pattern completion process. Correlations between particular feature values and particular categories acquired over the course of many learning trials scale the strength of visual inputs via the top-down projections of the Feature Expectation Neurons, $u_{\text{ft}_{\text{exp}}}(x, y, t)$ (as well as the feedback button, $u^*_{\text{fb}_{\text{exp}}}(t)$, but this is set to a constant as opposed to learned in these simulations). Inhibition of return (IOR) is applied at the previous position of the fovea, making it less likely to again be fixated. This inhibition is defined as:

$$u_{\text{ior}}(x, y, t) = \mathcal{G}_A(F^{-'}, \sigma_{22}) \tag{14}$$

The distribution of IOR defined in Equation 14 is a Gaussian, $\mathcal{G}_A$, centred at the location of the the previous fixation, $F^{-'}$. Attention shifting signals from the Saccade Motor Field, $u_{A \leftarrow M}(x, y, t)$, alter the spatial distribution of attention on the Spatial Attention Field, just as the SC can influence activity of LIP. This projection to the Spatial Attention Field is first convolved with its own kernel, $w_{A,M}$, in Equation 15, prior to being input to the field.

$$\begin{aligned} u_{A \leftarrow M}(x, y, t) = \int\int \big(& w_{A,M}(x - x', y - y', t) \\ & u^*_{\text{M}}(x', y', t) dx' dy' \big) \end{aligned} \tag{15}$$

A similar convolution, using the the kernel $w_{A,V}$, transforms the input to the Spatial Attention Field from the Visual Field in Equation 16.

$$\begin{aligned} u_{V \leftarrow A}(x, y, t) = \int\int \big(& w_{A,V}(x - x', y - y', t) \\ & u^*_{V}(x', y', t) dx' dy' \big) \end{aligned} \tag{16}$$

Saccade Motor Field

$$\begin{aligned} \tau \dot{u}_{\text{M}}(x, y, t) = & - u_{\text{M}}(x, y, t) + h_{M,47} \\ & - c_{48} u^*_{\text{x}}(t) \mathcal{G}_M(F, \sigma_{49}) \\ & + c_{16} u^*_g(t) u^*_{\text{M}}(x, y, t) \\ & + u_{M \leftarrow A}(x, y, t) + \zeta_M(x, y, t) \\ & + \int\int \big(w_{M,M}(x - x', y - y', t) \\ & \qquad u^*_{\text{M}}(x', y') dx' dy' \big) \\ & - c_{62} \sum u^*_{\text{M}}(x, y, t) \end{aligned} \tag{17}$$

Input from the Spatial Attention Field, $u_A(x, y, t)$, is the primary source of excitation to the Saccade Motor Field, the dynamics of which are formalized in Equation 17.

$$\begin{aligned} u_{A\leftarrow M}(x, y, t) =& c_{56}(1 - \mathcal{G}_M(F, \sigma_{55})) \\ & \int\int \big(w_{A\leftarrow M}(x - x', y - y', t) \\ & u^*_{\mathrm{A}}(x', y', t)dx'dy'\big) \end{aligned} \tag{18}$$

The projection from the Spatial Attention Field (Equation 18) is inhibited at the extant position of fixation F, as another way to mitigate continual refixation at the same location.

Inhibitory input from the Fixation Neuron, $u_{\mathrm{x}}(x, y, t)$, and the Gaze Change Neuron, $u_g(x, y, t)$, also project to the translated location of the fovea in order to allow the eye to move and to minimize refixations. Prior to convolving the Spatial Attention Field with its Saccade Motor Field specific kernel, additional Gaussian inhibition of the current foveal region is applied.

The location of a target on the Saccade Motor Field corresponds to the location with the largest activation at the moment a threshold level of activity in the Saccade Initiation Neuron is surpassed: $F' = \arg\max_{x,y}\{u_{\mathrm{M}}(x, y, t)|u^*_r \geq \theta_r\}$. As the Saccade Motor Field is reoriented during the saccade, it will start to relax to its resting level h_M.

In this version of LAG-1, the various forces that can affect the horizontal and vertical calculations of saccade amplitude are simplified to the Euclidean distance between the current point of fixation and the saccade target: $D = \|(F, F^{+'})\|$. An estimation of the time τ_D, needed to complete the saccade based on a predefined constant for velocity, υ_D is then calculated by $\tau_D = D/\upsilon_D$. In the brain, a multitude of factors, such as distance and direction of the movement, are known to influence the distinct pulse generators for horizontal and vertical movement of the eye. Once a saccade is initiated in Lag-1, the foveal position is updated at each time step along the path of the saccade.

Gaze Change Neuron

$$\begin{aligned} \tau\dot{u}_g(t) =& -u_g(t) + h_{g,63} - c_{64}u^*_r(t) \\ & + c_{65}u^*_{\mathrm{x}}(t) + c_{66}u^*_g(t) + c_{67}t_F + \zeta_g \end{aligned} \tag{19}$$

The Gaze Change Neuron, $u_g(t)$, defined in Equation 19, has three exogenous sources of input: it is inhibited by the Saccade Initiation Neuron $u_r(t)$, excited by the Fixation Neuron, $u_{\mathrm{x}}(t)$, and also excited by the Fixation Impatience, $t_F = (t - F^{-'}_t)^{\lambda_{P2}}$, where $F^{-'}_t$ is the time since the last saccade was initiated. Larger values of λ_{P2} translate into steeper growth in input to the Gaze Change neuron, leading to shorter fixations.

Fixation Neuron

$$\begin{aligned} \tau\dot{u}_x(t) =& -u_x(t) + h_{x,68} - c_{69}u^*_g(t) - c_{70}u^*_r(t) \\ & + c_{71}u^*_{\mathrm{x}}(t) + \zeta_x \\ & + c_{72}u^*_{\mathrm{ft_{det}}}(F_j, t) \end{aligned} \tag{20}$$

Changes in the activity of the Fixation Neuron, $u_{\mathrm{x}}(t)$, are described in Equation 20. Inhibitory input from the Saccade Initiation Neuron, $u_r(t)$, strongly suppresses the Fixation Neuron during a saccade. The Gaze Change Neuron, $u_g(t)$, also inhibits the Fixation Neuron in order to allow alternative locations to better attract attention prior to the initiation of a saccade. Finally, the stimulus input to the active Feature Detection Neuron, $u^*_{\mathrm{ft}_{det}}(j = F, t)$ also feeds into the Fixation Neuron allowing ongoing information processing to more directly affect the activity of the Fixation Neuron.

Saccade Initiation Neuron

$$\begin{aligned}\tau\dot{u}_r(t) = & -u_r(t) + h_r, 75 - c_{76}u_x^*(t) \\ & + c_{77}u_g^*(t) + c_{79}u_r^*(t) \\ & + c_{78}\max_{x,y} u_{\mathrm{M}}(x,y,t) + \zeta_r\end{aligned} \tag{21}$$

In Equation 21, the Saccade Initiation (**r**eset) Neuron, $u_r(t)$, is inhibited by the Fixation Neuron, $u_{\mathrm{x}}(t)$, excited by the Saccade Motor Field, $\max_{x,y} u_{\mathrm{M}}(x,y,t)$, and the Gaze Change Neuron, $u_g(t)$.

Feature Detection Neurons

$$\begin{aligned}\tau \dot{u_{\mathrm{ft_{det}}}}(j,t) = & -u_{\mathrm{ft_{det}}}(j,t) + h_{\mathrm{ft_{det}},80} \\ & + \zeta_{\mathrm{ft_{det}}}(t) \\ & + c_{82}u^*_{\mathrm{ft_{det}}}(j,t) \\ & + c_{83}u^*_{\mathrm{ft_{det}}}(j,t) - c_{81}u^*_{\mathrm{ft_{det}}}(j',t) \\ & + c_{84}\sum_j u^*_{\mathrm{ft_{det}}}(j,t)\end{aligned} \tag{22}$$

The activity of the Feature Detection Neurons, $u_{\mathrm{ft_{det}}}(j,t)$, is defined in Equation 22. The neuron representing the complementary feature value for the same spatial location is indexed by j'. Transduction of the stimulus attributes is defined in Equation 23 as a fovea sized integration over the jth layer of the the input to the Visual Field, where F_{Mask} is a Gaussian with parameters specified in the appendix and the active Feature Detection index j corresponds with the same index z_j along the feature dimension of the Visual Field.

$$u_{\mathrm{ft_{det}}}(j,t) = \int\int u_V^*(x,y,z_j)F_{Mask}(x,y,t)dx'dy' \tag{23}$$

Self-excitatory input, $u_{\mathrm{ft_{det}}}(j,t)$, is what provides the capacity for the neurons to act as a working memory that is constrained by the total capacity constrained activity of the other Feature Detection Neurons. As features are fixated, the neurons representing the previously viewed features decay down to their self-sustaining level, or are even forced out of their excited state. The activity of all the other Feature Detection Neurons is summed and subtracted from each detector as global inhibition.

Category Neurons

$$\begin{aligned}\tau\dot{u}_c(i,t) = & -u_c(i,t) + h_{c,97} + c_{99}u_c^*(i,t) \\ & + \sum_j c_{98}u_g(i,j,t)u^*_{\mathrm{ft_{det}}}(j,t) \\ & - c_{100}\sum_i u_c^*(i,t) \\ & + u^*_{\mathrm{fb_{det}}}(t)\mathbb{1}_{\mathrm{phase=feedback}}\mathbb{1}_{\mathrm{boost}\to u_c(i,t)} + \zeta_c(t)\end{aligned} \tag{24}$$

Activity from the Feature Detection Neurons, $u_{\mathrm{ft_{det}}}(j,t)$, passes through gain modulated connection weights, $u_g(i,j,t)$, defined in Equation 25, to selectively activate categories. Gain is calculated by taking the absolute difference in the connecting weights between the feature values connected to a particular category at a particular location in space. During the feedback phase, the correct category is boosted by multiplying together the activity of the Feedback Button Detector, $u^*_{\mathrm{fb_{det}}}$, and a constant $\mathbb{1}_{\mathrm{boost}\to u_c(i,t)}$.

$$u_g(i,j,t) = W(i,j,t) + c_{105}\,|W(i,j,t) - W(i,j',t)| \tag{25}$$

Feature to category association

On each time step of trial feedback, the weights connecting features and categories will undergo at least 2 of 3 possible types of associative learning (where the third type only occurs on error trials) as defined in Equation 26.

$$\tau \dot{W}(i,j,t) = dW_1(i,j,t) + dW_2(i,j,t) + dW_3(i,j,t) \tag{26}$$

On correct trials, only the first two terms of Equation 27 result in weight changes. The first weight change term, dW_1, moderately strengthens only those weights linking the correct category to active features in working memory according to the learning rate λ_{P1}, where the supervisory feedback signal for the correct category is given as input to the category neurons, according to Equation 24 at the start of the feedback phase.

$$\begin{aligned} dW_1(i,j,t) \quad &= \lambda_{P1} W(i > \theta_{131}, j > \theta_{w,c,130}, t) \\ &(1 - c(i > \theta_{w,c,130}, t) \\ &+ c(i > \theta_{130}, t) u_{\mathrm{ft_{det}}}(j > \theta_{131}, t)) \end{aligned} \tag{27}$$

Second, the entire weight matrix is incremented by a proportion of the learning rate in dW_2, reflecting a token amount of association between the task elements in general in Equation 27, and any of the responses. Although we set c_{126} and c_{127} to nominal values in these simulations, association like this would be needed to explain the intra category reversal learning advantage when compared to exra dimensional reversal learning [16].

$$dW_2(i,j,t) \quad = c_{126} + c_{127} \lambda_{P1} W(i,j,t) \tag{28}$$

The third term, dW_3 only applies to error trials, $\mathbb{1}_{\mathrm{error}}$, where a sharp anti-Hebbian decoupling between the response, $\mathbb{1}_{\mathrm{response}_i}$ and the active features is applied.

$$dW_3(i,j,t) = \begin{cases} -(c_{128} + c_{129}\lambda_{P1}) & \\ \quad W(\mathbb{1}_{\mathrm{response}_i}, j > \theta_{131}, t), & \text{if } \mathbb{1}_{\mathrm{error}} \\ 0, & \text{otherwise} \end{cases} \tag{29}$$

By subtracting off a proportion of the active weights from the weight change in Equation 27, Hebbian association slows down slightly as the weights get larger, similar again to earlier category learning models that annealed learning rates (e.g., [108, 151]).

Feature Expectation Neurons

$$\begin{aligned} \tau \dot{u_{\mathrm{ft_{exp}}}}(j,t) = & - u_{\mathrm{ft_{exp}}}(j,t) + h_{\mathrm{ft_{exp}},106} \\ & - u^*_{\mathrm{ft_{exp}}}(j',t) - c_{108} u^*_{\mathrm{ft_{det}}}(j,t) \\ & + c_{109} \sum_i u_c(i,t) c_{107} u_g(i,j,t) \\ & + c_{110} u^*_{\mathrm{ft_{exp}}}(j,t) - \sum_{m \neq j} u^*_{\mathrm{ft_{exp}}}(m,t) \\ & + \zeta_{\mathrm{ft^*_{exp}}}(t) \end{aligned} \tag{30}$$

Activation of the Feature Expectation Neurons, $u_{\mathrm{ft_{exp}}}(j,t)$, as defined in Equation 30, is primarily driven by input from the Category Neurons, $u_c(i,t)$, after scaling the weights using the gain function, $u_g(i,j,t)$. The Feature Detection Neurons $u_{\mathrm{ft_{det}}}(j,t)$ inhibit the Feature Expectation Neurons representing the same values in order to distinguish what is known so far in the trial from what has yet to be looked at. As with the Feature Detection Neurons, complementary feature values, denoted by $u_{\mathrm{ft_{exp}}}(j',t)$, inhibit one another. Finally, global inhibition suppresses each Feature Expectation Neuron according the total activation energy of all the others.

Click Decision Neuron

$$\begin{aligned}\tau \dot{u}_d(t) = & -u_d(t) + h_{d,107} - (c_{114} u_c^*(t))^2 \\ & + c_{115} u_{\text{impatience}_{\text{trial}}}{}^*(t) - c_{116} u_{\text{x}}^*(t) + \zeta_d(t)\end{aligned} \tag{31}$$

The Click Decision Neuron, $u_d(t)$, is increasingly excited by impatience, $u_{\text{impatience}_{\text{trial}}}(t)$, over the course of the trial. Larger maximum Category Neuron activation is a much stronger influence on the decision to respond than smaller levels. This is modelled in Equation 31, by squaring the maximum value of the Category Neurons. An inhibitory input from the Fixation Neuron $u_{\text{x}}(t)$, was also needed in order to have LAG-1 complete fixations prior to responding. Trial Impatience is modelled by its own differential equation in Equation 32.

$$\begin{aligned}\tau \dot{u}_{\text{impatience}_{\text{trial}}}(t) = & -u_{\text{impatience}_{\text{trial}}}(t) + h_{\text{impatience}_{\text{trial}},120} \\ & + c_{123}(t - (\mathbb{1}_{\text{phase=feedback}_t}))^{\lambda_{P3}} \\ & + \zeta_{\text{impatience}_{\text{trial}}}(t)\end{aligned} \tag{32}$$

The indicator function $\mathbb{1}_{\text{phase=feedback}}$ is set to 1 if the feedback phase has been initiated, effectively resetting the model's impatience relative to the start time of the feedback phase, $\mathbb{1}_{\text{phase=feedback}_t}$.

The selection of a particular category i, as a final response when the Click Decision Neuron crosses its decision threshold, is the result of a stochastic decision, given by Equation 33:

$$P(i) = \frac{e^{u_{c_i/T}}}{\sum_i e^{u_{c_i/T}}} \tag{33}$$

The use of the Softmax rule specifies one way to make choices under conditions of uncertainty. Neurologically this calculation relies on prefrontal circuits that can differentially weight sensorimotor competitions for different body parts mapped parietally [99]. A choice of strategy, such as the decision to exploit current knowledge or explore the space, is itself a kind of category decision. The Softmax temperature parameter, T, scales the effect that differences in activation will have on the probability of choice. When the temperature is high, the decision choices move toward being equally likely. When the temperature is lower, the choice with the highest activation becomes increasingly favoured.
