# Supplementary material for: LAG-1: A dynamic, integrative model of learning, attention, and gaze
Source: PLoS One. 2022 Mar 17;17(3):e0259511. doi: 10.1371/journal.pone.0259511 (PMC8929614; doi:10.1371/journal.pone.0259511)
Supplement: S3 Appendix — (PDF) [file pone.0259511.s003.pdf]

S3 Appendix. Fitting procedure.

In Simulation 1, the target fit vector contained four measures (accuracy, fixation count, the probability of fixating irrelevant information on a trial, and the average fixation durations). The data on each of these measures was summarized by an initial intercept and a slope of change in the measure over the experiment, yielding a target vector with eight elements for each human subject. In Simulation 2, the vector included the individual subject transfer probabilities (for all 16 stimuli), their learning points (the trial number on which the participant began two consecutive error-free blocks) and a measure of the changes in the allocation of attention to stimulus features of high and low diagnosticity. We were unable to precisely reconstruct all of the individual subject data reported in [10] for the attentional allocation component of the fit measure so we instead used the reported averages in three measures of attentional allocation. The first two numbers were the average starting and ending fixation counts to the individual features. The third component was the difference between the least informative (Feature two) and the most informative features (Feature one) on this measure (∼0.3). The target vector for Simulation 2 thus had 19 elements. For both vectors we used a residual sum of squares measure of error to calculate the best model for each subject (for precise details see Table 8).

There are, as indicated in the model's formal description, many parameters that need to be specified in the model (for example, sigmoids and kernels need to be given a precise shape in order to perform the calculations). We fixed these in advance of the simulations by picking sensible values and then, where necessary, tweaked things till the model had stable performance that roughly resembled human looking and learning. No effort was made at this stage to fit the myriad of findings we address in the simulation; stability was our only concern. Once the model was performing sensibly, everything was fixed and we proceeded with the fitting procedure.

We began the process of finding the best fitting parameter values by running the model with a plausible initial set of low, medium and high levels of the three free parameters: generating a parameter cube with 27 data points. At each of these levels, we ran the model multiple times and took its average behaviour to account for the stochasticity in the model. A measure of fit was generated by comparing every individual human subject with the data of the model at those 27 locations in parameter space. If the best fitting parameters for a particular individual subject was adjacent to unexplored regions of the parameter space, then the cube was extended on that side, and new simulations were run. Simulations were ended only when every individual was surrounded by 26 worse fitting points.

In order to plot the simulations for comparison with the human subjects we needed to collapse the group of simulations at the best fitting set of parameter values for each subject. To do this we simply averaged over the the experiment length data for the measures of interest. We also show the model's variability in the error bars we report. The result is that we have model performance data comparable to that of each human participant, and can plot the model data in exactly the same way as the human data.
