# Supplementary material for: LAG-1: A dynamic, integrative model of learning, attention, and gaze
Source: PLoS One. 2022 Mar 17;17(3):e0259511. doi: 10.1371/journal.pone.0259511 (PMC8929614; doi:10.1371/journal.pone.0259511)
Supplement: S4 Appendix — (PDF) [file pone.0259511.s004.pdf]

S4 Appendix. Supplementary equation parameters.

Table 5. Supplementary equation parameters

Parameter	Arguments
**Noise Kernel Extras**	$w_\zeta = [c_5, 1]$
**Visual Field extras**	$u_V^* = f_{\beta_{10},\mu_{0,11}}(u_V(x,y,t))$<br>$w_V = [c_{6,\sigma 7,c8,\sigma_9}]$<br>$u_{\text{ft}_{\text{exp}}}^* = f_{\beta_{15},\mu_{0,16}}(u_{\text{ft}_{\text{exp}}}(z,t))$<br>$\zeta_V = [c_5, 1]$<br>$u_{\text{fb}_{\text{exp}}}^* = f_{\beta_{23},\mu_{0,24}}(u_{\text{fb}_{\text{exp}}}(t))$
**Spatial Attention Field extras**	$u_A^* = f_{\beta_{32},\mu_{0,33}}(u_A(x,y,t))$<br>$\zeta_A = [c_{39}, c_{40}]$<br>$w_{A,A} = [c_{34}, \sigma_{35}, c_{36}, \sigma_{37}]$<br>$w_{A,M} = [c_{41}, \sigma_{42}]$
**Saccade Motor Field extras**	$w_{M,M} = [c_{53}, \sigma_{54}]$<br>$u_M^* = f_{\beta_{43},\mu_{0,44}}(u_{\text{M}}(x,y,t))$<br>$w_{M,M} = [c_{59}, \sigma_{60}, c_{61}, \sigma_{62}]$<br>$\zeta_{M \leftarrow A} = [c_{39}, c_{40}]$
**Gaze Change Neuron extras**	$\zeta_g = [c_{89}, c_{90}]$
**Saccade Initiation Neuron extras**	$\zeta_r = [c_{93}, c_{94}]$<br>$u_r^* = f_{\beta_{26},\mu_{0,27}}(u_r(t))$
**Fixation Neuron extras**	$u_{\text{x}_{\text{ft}_{\text{det}}}}^* = f_{\beta_{73},\mu_{0,74}}(\text{x}_{u_{\text{ft}_{\text{det}}}}(t))$<br>$\zeta_x = [c_{91}, c_{92}]$<br>$u_{\text{x}}^*(t) = f_{\beta_{51},\mu_{0,51}}(\text{x}^*(t))$
**Feature Detection Extras**	$u_{\text{ft}_{det}}^*(j,t) = f_{\beta_{85},\mu_{0,86}}(u_{\text{ft}_{det}}(t))$<br>$F_{Mask} = \mathcal{G}[\mu = (0,0), \sigma = (3,3)]$<br>$u_{\text{ft}_{det,f,V}}^*(j,t) = f_{\beta_{87},\mu_{0,88}}(u_{\text{ft}_{det,f,V}}(j,t))$<br>$\zeta_{u_{\text{ft}_{\text{det}}}} = [c_{95}, c_{96}]$
**Category Neuron extras**	$u_c^*(i,t) = f_{\beta_{101},\mu_{0,102}}(u_c(i,t))$<br>$\zeta_c = [c_{103}, c_{104}]$
**Feature Expectation Neuron extras**	$\zeta_{\text{ft}_{\text{exp}}} = [c_{111}, c_{112}]$
**Decision Neuron extras**	$u_d^*(t) = f_{\beta_{116},\mu_0,117}(u_c(i,t))$<br>$\zeta_d = c_{118}, c_{119}$
**Trial Impatience extras**	$u_{\text{impatience}_{\text{trial}}}(t)^* = f_{\beta_{120},\mu_0,121}(u_{\text{impatience}_{\text{trial}}}(t))$<br>$\zeta_{\text{impatience}_{\text{trial}}} = [c_{124}, c_{125}]$
