# Supplementary material for: LAG-1: A dynamic, integrative model of learning, attention, and gaze
Source: PLoS One. 2022 Mar 17;17(3):e0259511. doi: 10.1371/journal.pone.0259511 (PMC8929614; doi:10.1371/journal.pone.0259511)
Supplement: S5 Appendix — (PDF) [file pone.0259511.s005.pdf]

S5 Appendix. Parameter tables and best fits.

Table 6. Parameter changes between Simulation 1 and Simulation 2.

Parameter	Simulation 1	Simulation 2
$c_{c,\mathrm{ft}_{\mathrm{det}},98}$	1	4.6
$c_{M,+A,53}$	7	12
$c_{d,k,113}$	4	2
$c_{c,g,105}$	1	0.2
$\mathbb{1}_{\mathrm{boost}}$	0.3	0.7

Table 7. Simulation 1: Covariance parameters.

Measure	Covariance
Average accuracy	0.00001
Accuracy slope	0.0001
Average fixation count	1
Fixation count slope	1
Average probability of irrelevant fixation	1
Probability of irrelevant fixation slope	1
Average fixation duration	1
Fixation duration slope	1

Table 8. Simulation 2: Covariance parameters.

Measure	Covariance
Exemplar A1 at Transfer	0.0001
Exemplar A2 at Transfer	0.0001
Exemplar A3 at Transfer	1
Exemplar A4 at Transfer	1
Exemplar A5 at Transfer	1
Exemplar B1 at Transfer	0.0001
Exemplar B2 at Transfer	0.001
Exemplar B3 at Transfer	0.1
Exemplar B4 at Transfer	1
Exemplar T1 at Transfer	1
Exemplar T2 at Transfer	1
Exemplar T3 at Transfer	1
Exemplar T4 at Transfer	1
Exemplar T5 at Transfer	1
Exemplar T6 at Transfer	1
Exemplar T7 at Transfer	1
Criterion point	1
Feature 1 first half fixation count	0.001
Feature 1 and Feature 2 second half difference	0.001

Table 9. Specifies the experiment parameters for Simulation 1 and Simulation 2.

**Parameter**	**Simulation 1**	**Simulation 2**
Feature placement	F1:(51,68), F2:(66,43), F3:(36,43), Feedback:(33,71)	F1:(39,63), F2:(63,63), F3:(63,39), F4:(39,39), Feedback:(33,71)
Fovea size, (visual angle °)	$\sigma^2 = 3 = 1.7°$	$\sigma^2 = 3 = 1.6°$
Feature diameter	6.25	6.25
Visual Field (u_V) size (px)	101x101	101x101
Spatial Attention Field (u_A) size (px)	51x51	51x51
Saccade Motor Field (u_M) size (px)	51x51	51x51
Timescale, τ	10	10
Characteristic time, δt (5ms)	2	2
Temperature	0.07	0.07
Response threshold	0.8	0.8
Feedback threshold	0.98	0.98
Saccade threshold, θ_r	0.2	0.2

Table 10. All fixed parameter values.

Name	Value	Name	Value	Name	Value	Name	Value
$h_{V,1}$	-4	$c_{A,+A,34}$	5	$c_{g,\mathrm{imp}_{\mathrm{fixation}},67}$	0.0001	$c_{c,-c,100}$	3.5
$c_{V,I,2}$	4	$\sigma_{A,+A,35}$	5	$h_{\mathrm{x},68}$	-3.5	$\beta_{c,101}$	1.37
$c_{V,r,3}$	3	$c_{A,-A,36}$	8	$c_{\mathrm{x},g,69}$	-0.4	$\mu_{0,c,102}$	0.15
$c_{V,\mathrm{fb}_{\mathrm{nov}},4}$	10,1	$\sigma_{A,-A,37}$	7.5	$c_{\mathrm{x},r,70}$	-3	$\zeta_{c,T,103}$	0.02
$\zeta_{v,T,5}$	0.005	$c_{-A_{gi},38}$	0.2	$c_{\mathrm{x},\mathrm{x},71}$	3	$\zeta_{c,\delta,104}$	0.2
$c_{V,+V,6}$	1	$\zeta_{A,T,39}$	0.1	$c_{\mathrm{x},\mathrm{ft}_{\mathrm{det}},72}$	5	$c_{c,g,105}$	1
$\sigma_{V,+V,7}$	1.75	$\zeta_{A,\delta,40}$	15	$\beta_{\mathrm{x},\mathrm{ft}_{\mathrm{det}},73}$	1	$h_{\mathrm{ft}_{\mathrm{exp}},106}$	-3
$c_{V,-V,8}$	1	$c_{A,+M,41}$	8	$\mu_{0,u_{\mathrm{x}},\mathrm{ft}_{\mathrm{det}},74}$	1	$h_{d,107}$	-5
$\sigma_{V,-V,9}$	3.75	$\sigma_{A,M,42}$	2.5	$h_{r,75}$	-5	$c_{\mathrm{ft}_{\mathrm{exp}},f,108}$	0.01
$\beta_{V,10}$	2	$\beta_{M,43}$	4	$c_{r,\mathrm{x},76}$	-10	$c_{\mathrm{ft}_{\mathrm{exp}},c,109}$	2
$\mu_{0,V,11}$	2	$\mu_{0,M,44}$	0	$c_{r,g,77}$	4	$c_{\mathrm{ft}_{\mathrm{exp}},+\mathrm{ft}_{\mathrm{exp}},110}$	1.5
$c_{V,-A,12}$	0.001	$c_{A,+V,45}$	70	$c_{r,M,78}$	8	$\zeta_{\mathrm{ft}_{\mathrm{exp}},T,111}$	0.1
$c_{v,+\mathrm{ft}_{\mathrm{exp}},13}$	3	$\sigma_{A,+V,46}$	1.25	$c_{r,r,79}$	1.4	$\zeta_{\mathrm{ft}_{\mathrm{exp}},\delta,112}$	1
$c_{V,-\mathrm{ft}_{\mathrm{exp}},14}$	0.04	$h_{M,47}$	-2	$h_{\mathrm{ft}_{\mathrm{det}},80}$	-2	$c_{d,k,113}$	4
$\beta_{\mathrm{ft}_{\mathrm{exp}},15}$	0.5	$c_{M,\mathrm{x},48}$	-50	$c_{\mathrm{ft}_{\mathrm{det}},-\mathrm{ft}_{\mathrm{det}},81}$	4	$c_{d,\mathrm{imp}_{\mathrm{trial}},114}$	1.6
$\sigma_{0,\mathrm{ft}_{\mathrm{exp}},16}$	0	$\sigma_{M,-M,49}$	5	$c_{\mathrm{ft}_{\mathrm{det}},v,82}$	500	$c_{d,x,115}$	-1.2
$\sigma_{\zeta,17}$	5	$\beta_{x,50}$	0.5	$c_{\mathrm{ft}_{\mathrm{det}},\mathrm{ft}_{\mathrm{det}},83}$	4.6	$\beta_{d,116}$	2.5
$h_{A,18}$	-0.5	$\mu_{0,\mathrm{x},51}$	0	$c_{\mathrm{ft}_{\mathrm{det}},gi,84}$	0.1	$\mu_{0,d,117}$	1
$c_{A,+\mathrm{ft}_{\mathrm{exp}},19}$	0.2	$c_{M,g,52}$	30	$\beta_{\mathrm{ft}_{\mathrm{det}},85}$	1	$\zeta_{d,T,118}$	0.1
$c_{A,+\mathrm{x},20}$	5	$c_{M,+A,53}$	7	$\mu_{0,\mathrm{ft}_{\mathrm{det}},86}$	0	$\zeta_{d,\delta,119}$	0.1
$c_{A,-i,21}$	0.3	$\sigma_{M,+A,54}$	3.75	$\beta_{\mathrm{ft}_{\mathrm{det}}I,87}$	1	$\beta_{\mathrm{imp}_{\mathrm{trial}},120}$	0.01
$\sigma_{A,I,22}$	2.5	$\sigma_{M,-F,55}$	4	$\mu_{\mathrm{ft}_{\mathrm{det}}I,88}$	0	$\mu_{0,\mathrm{imp}_{\mathrm{trial}},121}$	300
$\beta_{\mathrm{fb}_{\mathrm{exp}},23}$	2	$c_{M,-F,56}$	3	$\zeta_{g,T,89}$	0.001	$h_{\mathrm{imp}_{\mathrm{trial}},122}$	1
$\mu_{0,\mathrm{fb}_{\mathrm{exp}},24}$	2	$\zeta_{M,T,57}$	0.2	$\zeta_{g,\delta,90}$	0.1	$c_{\mathrm{imp}_{\mathrm{trial}},T,123}$	0.001
$c_{A,\mathrm{fb}_{\mathrm{Button}},25}$	5	$\zeta_{M,\delta,58}$	1	$\zeta_{u_{\mathrm{x}},T,91}$	0.01	$\zeta_{\mathrm{imp}_{\mathrm{trial}},T,124}$	0.01
$\beta_{r,26}$	2.5	$c_{M,+M,59}$	5	$\zeta_{\mathrm{x},\delta,92}$	0.1	$\zeta_{\mathrm{imp}_{\mathrm{trial}},\delta,125}$	1
$\mu_{0,r,27}$	2.5	$c_{M,-M,60}$	8	$\zeta_{r,T,93}$	0.005	$c_{w,126}$	0
$c_{A,+g,28}$	-5	$\sigma_{M,-M,61}$	7.5	$\zeta_{r,\delta,94}$	0.1	$c_{w,127}$	0.2
$c_{A,+r,29}$	-10	$c_{-M,62}$	5	$\zeta_{\mathrm{ft}_{\mathrm{det}},T,95}$	0.02	$c_{w,128}$	10
$\beta_{g,30}$	3	$h_{g,63}$	-1	$\zeta_{\mathrm{ft}_{\mathrm{det}},\delta,96}$	0.2	$c_{w,129}$	0.33
$\mu_{0,g,31}$	0	$c_{g,r,64}$	-1	$h_{c,97}$	-0.5	$\theta_{w,c,130}$	0
$\beta_{A,32}$	1	$c_{g,\mathrm{x},65}$	0.2	$c_{c,\mathrm{ft}_{\mathrm{det}},98}$	4.6	$\theta_{w,\mathrm{ft}_{\mathrm{exp}},131}$	-1
$\mu_{0,A,33}$	0	$c_{g,g,66}$	0.5	$c_{c,+c,99}$	1.2		

Each parameter appears in the text with only the numerical subscript.

Table 11. Best fitting parameters: Simulation 1.

Subject	Learning Rate	Fixation Impatience	Trial Impatience	RSS
Subject-1	1.40e-05	1.75	1.65	-13411
Subject-2	1.65e-05	1.70	1.65	-11553
Subject-3	2.50e-06	1.65	1.85	-16749
Subject-4	1.65e-05	1.65	1.60	-12057
Subject-5	4.50e-06	1.50	1.75	-45454
Subject-6	1.25e-05	1.65	1.85	-11144
Subject-7	1.10e-05	1.90	1.65	-21914
Subject-8	1.40e-05	1.70	1.90	-11146
Subject-9	1.15e-05	1.55	1.85	-23273
Subject-10	1.65e-05	1.70	1.65	-11553
Subject-11	1.45e-05	1.80	1.90	-16196
Subject-12	6.00e-06	1.85	2.00	-22008
Subject-13	1.20e-05	1.50	1.80	-42564
Subject-14	7.50e-06	1.75	1.75	-12927
Subject-15	2.50e-06	1.65	1.85	-16749
Subject-16	7.50e-06	1.75	1.65	-12753
Subject-17	1.50e-05	1.80	1.65	-16005
Subject-18	1.30e-05	1.75	1.95	-13570
Subject-19	5.50e-06	1.70	1.65	-11025
Subject-20	6.00e-06	1.80	1.65	-15396
Subject-21	1.25e-05	1.50	1.75	-42805
Subject-22	1.40e-05	1.70	1.90	-11146
Subject-23	6.00e-06	1.70	1.85	-13640
Subject-24	1.55e-05	1.65	1.65	-11506
Subject-25	1.60e-05	1.70	1.65	-11700
Subject-26	1.25e-05	1.60	1.80	-14213
Subject-27	1.45e-05	1.85	1.85	-18727
Subject-28	1.25e-05	1.65	1.85	-11144
Subject-29	1.25e-05	1.60	1.85	-15218
Subject-30	1.45e-05	1.75	1.75	-12969
Subject-31	1.40e-05	1.75	1.65	-13411
Subject-32	1.30e-05	1.75	1.95	-13570
Subject-33	1.65e-05	1.65	1.60	-12057
Subject-34	1.25e-05	1.50	1.75	-42805
Subject-35	1.40e-05	1.75	1.75	-12938
Subject-36	8.00e-06	1.80	2.05	-20832
Subject-37	7.50e-06	1.65	1.75	-12477
Subject-38	1.30e-05	1.70	1.75	-11241
Subject-39	1.25e-05	1.60	1.85	-15218
Subject-40	6.50e-06	1.70	1.65	-11058
Subject-41	1.25e-05	1.70	1.65	-11119
Subject-42	1.40e-05	1.75	1.65	-13411

Table 12. Best fitting parameters: Simulation 2.

Subject	Learning Rate	Fixation Impatience	Trial Impatience	RSS
Subject-1	1.75E-05	1.45	1.65	-3491
Subject-2	4.50E-06	2.30	2.20	-3845
Subject-3	2.00E-06	1.85	1.90	-4224
Subject-4	6.00E-06	1.65	1.85	-2536
Subject-5	1.05E-05	1.65	1.85	-2352
Subject-6	9.50E-06	1.55	1.75	-4799
Subject-7	9.50E-06	1.60	1.80	-2614
Subject-8	1.75E-05	1.45	1.65	-3491
Subject-9	5.50E-06	1.60	1.80	-2921
Subject-10	1.75E-05	1.45	1.65	-3491
Subject-11	9.50E-06	1.60	1.80	-2614
Subject-12	5.50E-06	1.60	1.80	-2921
Subject-13	8.00E-06	2.25	2.15	-3456
Subject-14	5.50E-06	1.60	1.80	-2921
Subject-15	5.00E-06	1.65	1.90	-5188
Subject-16	1.05E-05	1.65	1.85	-2352
Subject-17	5.50E-06	1.60	1.80	-2921
Subject-18	9.50E-06	1.55	1.75	-4799
Subject-19	1.10E-05	1.70	1.85	-2201
Subject-20	3.00E-06	2.30	2.20	-5019
Subject-21	1.75E-05	1.45	1.65	-3491
Subject-22	5.50E-06	1.60	1.80	-2921
Subject-23	1.75E-05	1.45	1.65	-3491
Subject-24	6.50E-06	1.95	2	-3996
Subject-25	1.75E-05	1.45	1.65	-3491
Subject-26	6.50E-06	1.55	1.75	-2647
Subject-27	2.50E-06	2.15	2.20	-4219
Subject-28	6.50E-06	1.55	1.75	-2647
Subject-29	1.05E-05	1.65	1.85	-2352
Subject-30	1.75E-05	1.45	1.65	-3491
Subject-31	1.10E-05	1.70	1.85	-2201
Subject-32	1.75E-05	1.45	1.65	-3491
Subject-33	6.50E-06	1.55	1.75	-2647
Subject-34	1.75E-05	1.45	1.65	-3491
Subject-35	1.75E-05	1.45	1.65	-3491
Subject-36	6.50E-06	1.95	2	-3996
Subject-37	1.75E-05	1.45	1.65	-3491
Subject-38	1.75E-05	1.45	1.65	-3491
Subject-39	1.75E-05	1.45	1.65	-3491
Subject-40	4.50E-06	1.55	1.75	-2750
Subject-41	1.75E-05	1.45	1.65	-3491
Subject-42	6.50E-06	1.55	1.75	-2647
Subject-43	1.05E-05	1.65	1.85	-2352
Subject-44	1.05E-05	1.65	1.85	-2352
Subject-45	8.50E-06	1.60	1.80	-3887
Subject-46	1.75E-05	1.45	1.65	-3491
Subject-47	8.00E-06	1.80	1.95	-2855

Continued on next page

Table 12 – continued from previous page

Subject	Learning Rate	Fixation Impatience	Trial Impatience	RSS
Subject-48	8.00E-06	1.80	1.95	-2855
Subject-49	5.50E-06	1.50	1.75	-3752
Subject-50	1.75E-05	1.45	1.65	-3491
Subject-51	3.50E-06	1.55	1.75	-2229
Subject-52	4.50E-06	2.25	2.15	-4997
Subject-53	2.00E-06	1.70	1.90	-5253
Subject-54	3.00E-06	2	2.10	-4547
Subject-55	6.00E-06	2.15	2.10	-4743
Subject-56	6.50E-06	1.55	1.75	-2647
Subject-57	1.10E-05	1.70	1.85	-2201
Subject-58	4.50E-06	1.90	2	-3674
Subject-59	6.50E-06	1.55	1.75	-2647
Subject-60	9.50E-06	1.55	1.75	-4799
Subject-61	6.50E-06	1.55	1.75	-2647
Subject-62	9.50E-06	1.55	1.75	-4799
Subject-63	3.50E-06	2.45	2.25	-6432
Subject-64	1.05E-05	1.65	1.85	-2352
