# Supplementary material for: LAG-1: A dynamic, integrative model of learning, attention, and gaze
Source: PLoS One. 2022 Mar 17;17(3):e0259511. doi: 10.1371/journal.pone.0259511 (PMC8929614; doi:10.1371/journal.pone.0259511)
Supplement: S6 Appendix — (PDF) [file pone.0259511.s006.pdf]

S6 Appendix. Individual fit visualizations.

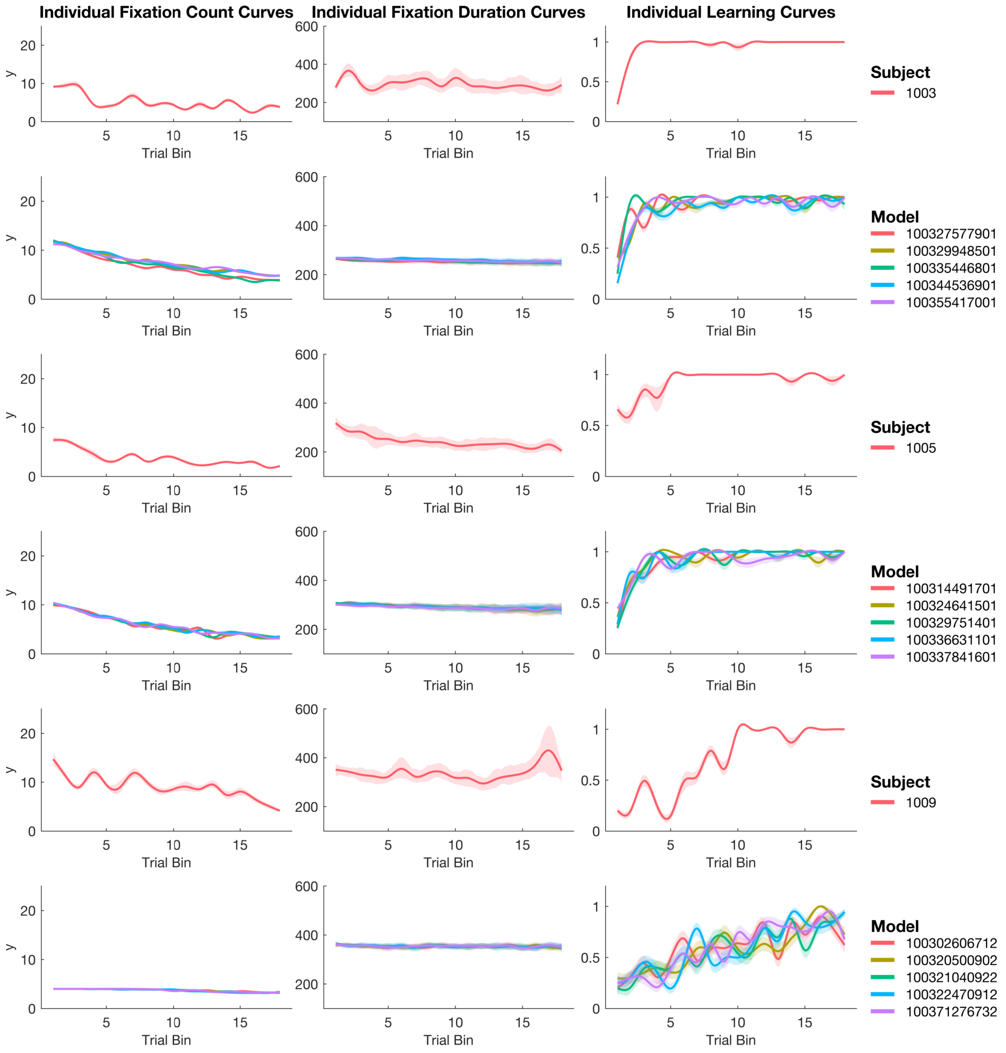

Figure 21. Subjects: 1003, 1005, 1008. Subject fixation counts, fixation durations, and learning curves are contrasted with their best fitting sample of models in the next row.

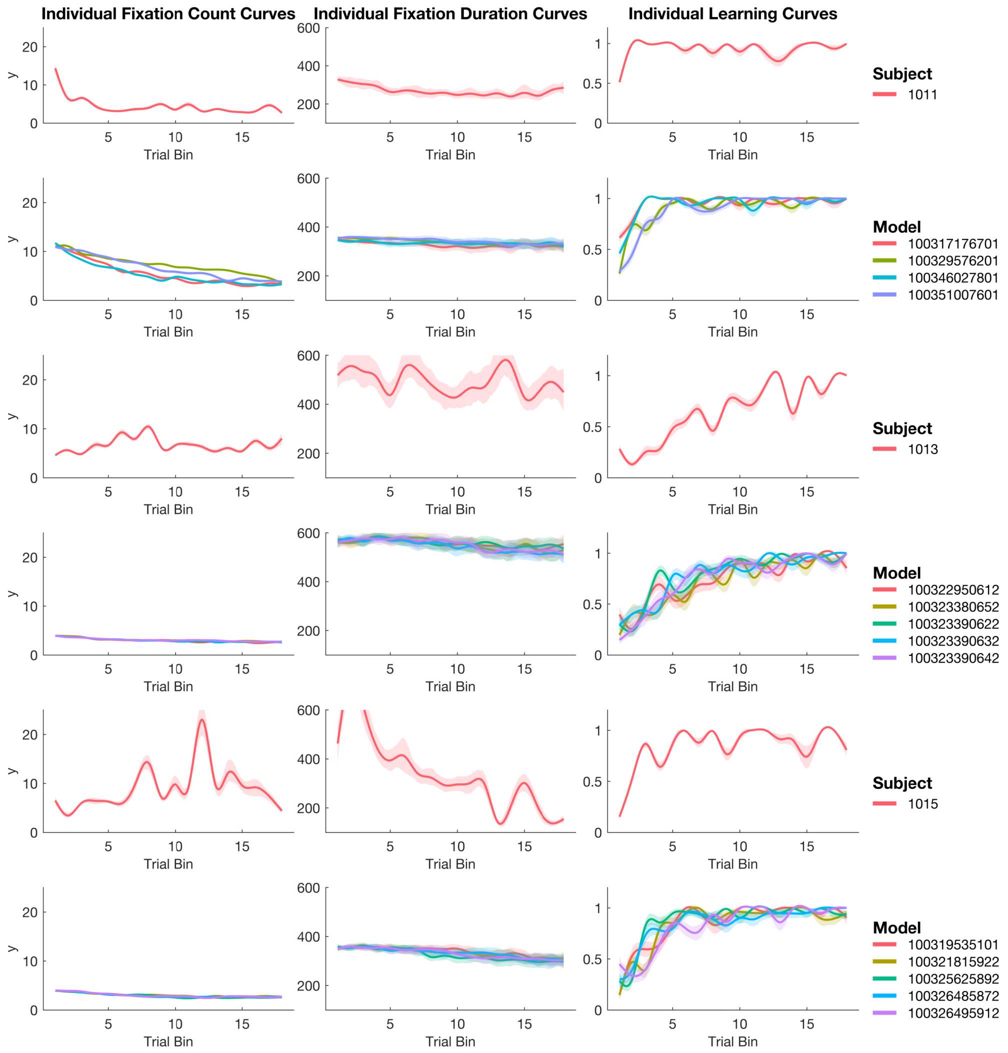

Figure 22. Subjects: 1011, 1013, 1015. Subject fixation counts, fixation durations, and learning curves are contrasted with their best fitting sample of models in the next row.

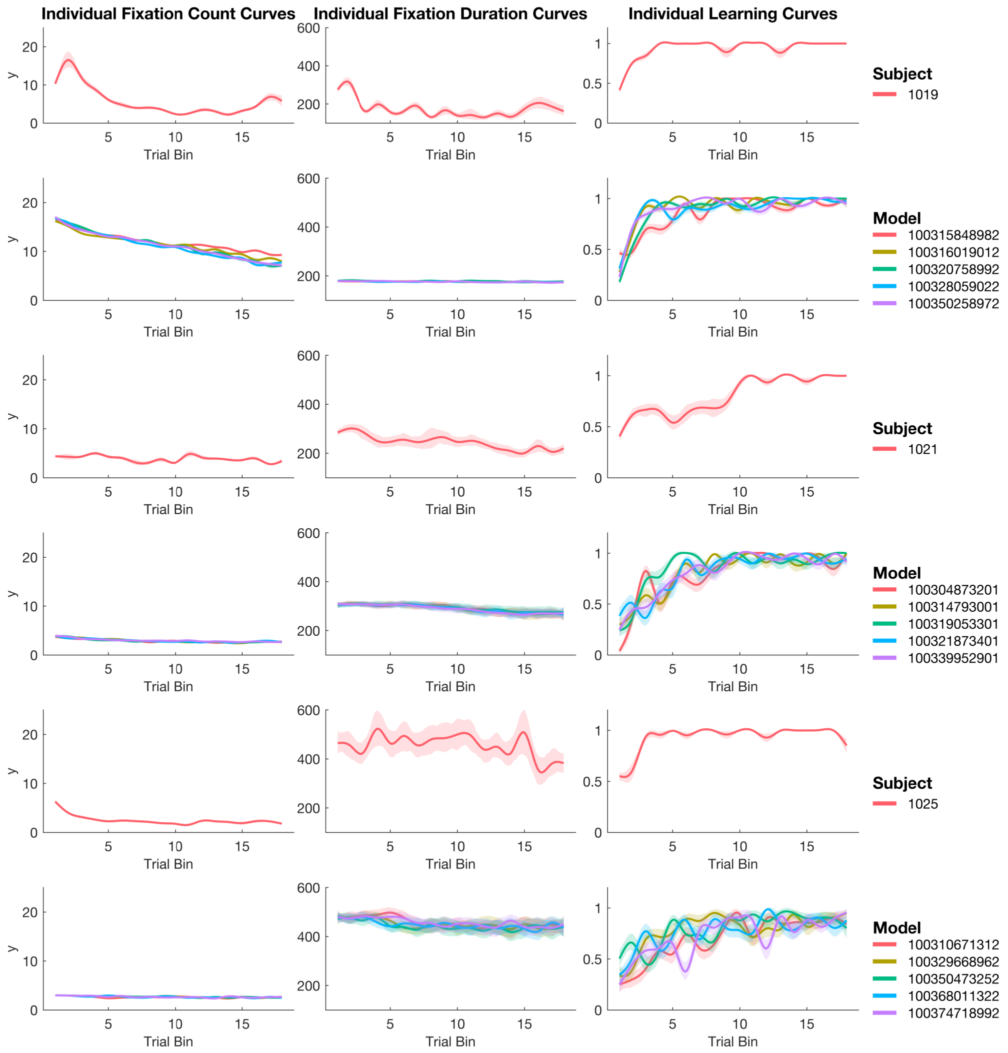

Figure 23. Subjects: 1019, 1021, 1025. Subject fixation counts, fixation durations, and learning curves are contrasted with their best fitting sample of models in the next row.

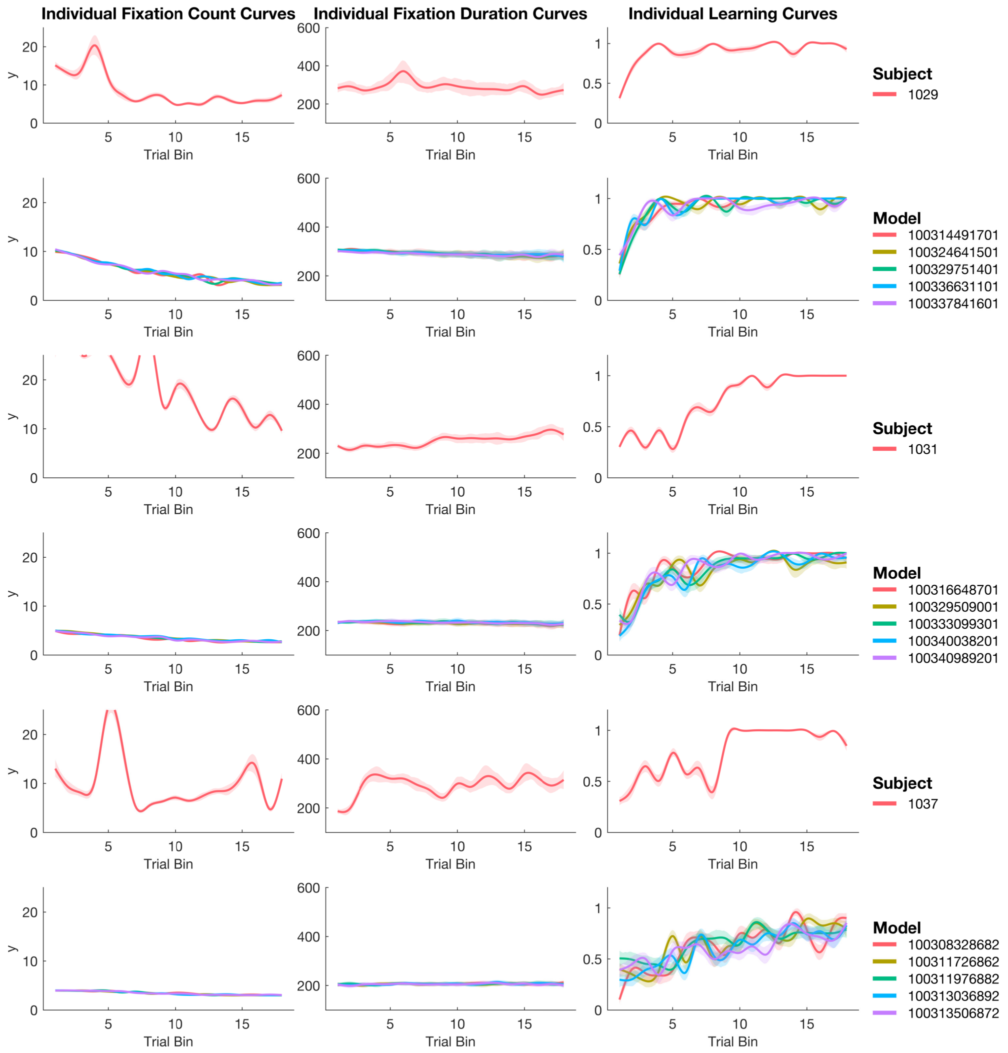

Figure 24. Subjects: 1029, 1031, 1037. Subject fixation counts, fixation durations, and learning curves are contrasted with their best fitting sample of models in the next row.

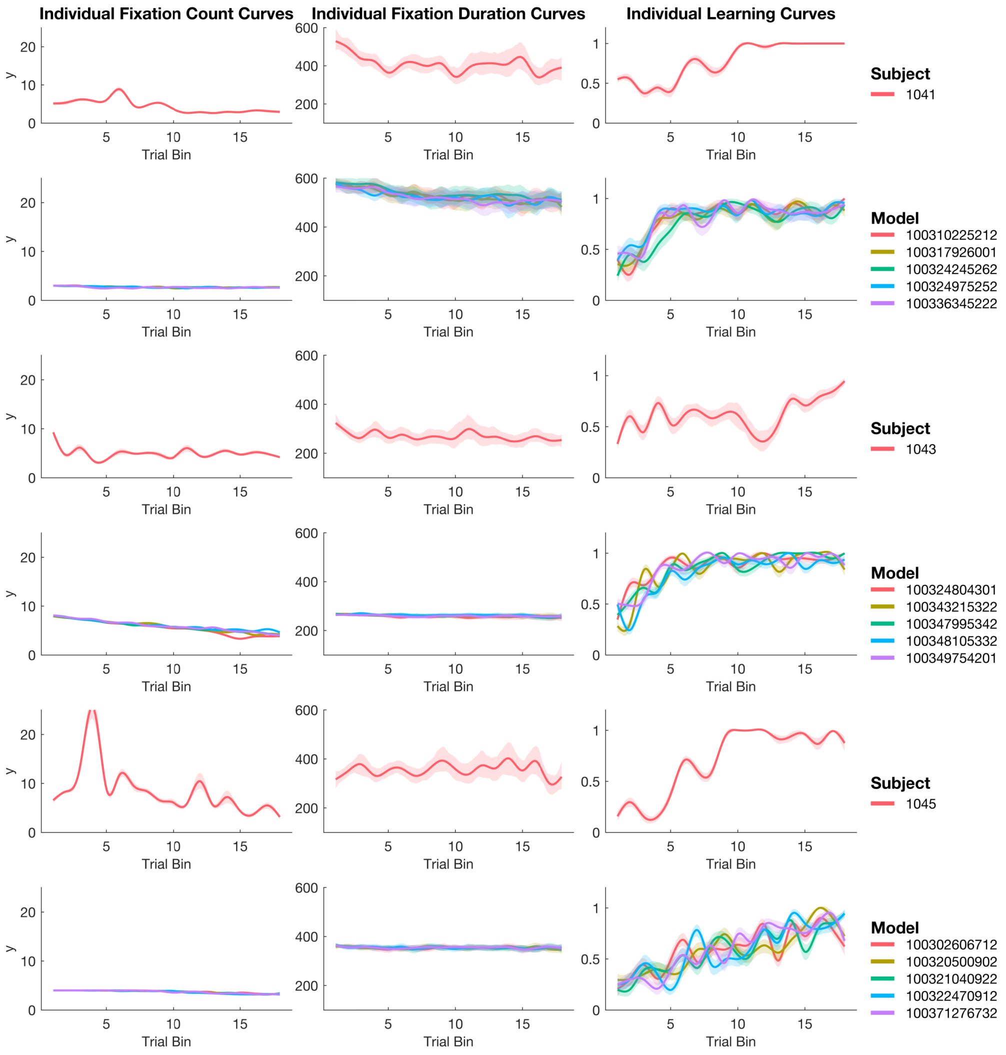

Figure 25. Subjects: 1041, 1043, 1045. Subject fixation counts, fixation durations, and learning curves are contrasted with their best fitting sample of models in the next row.

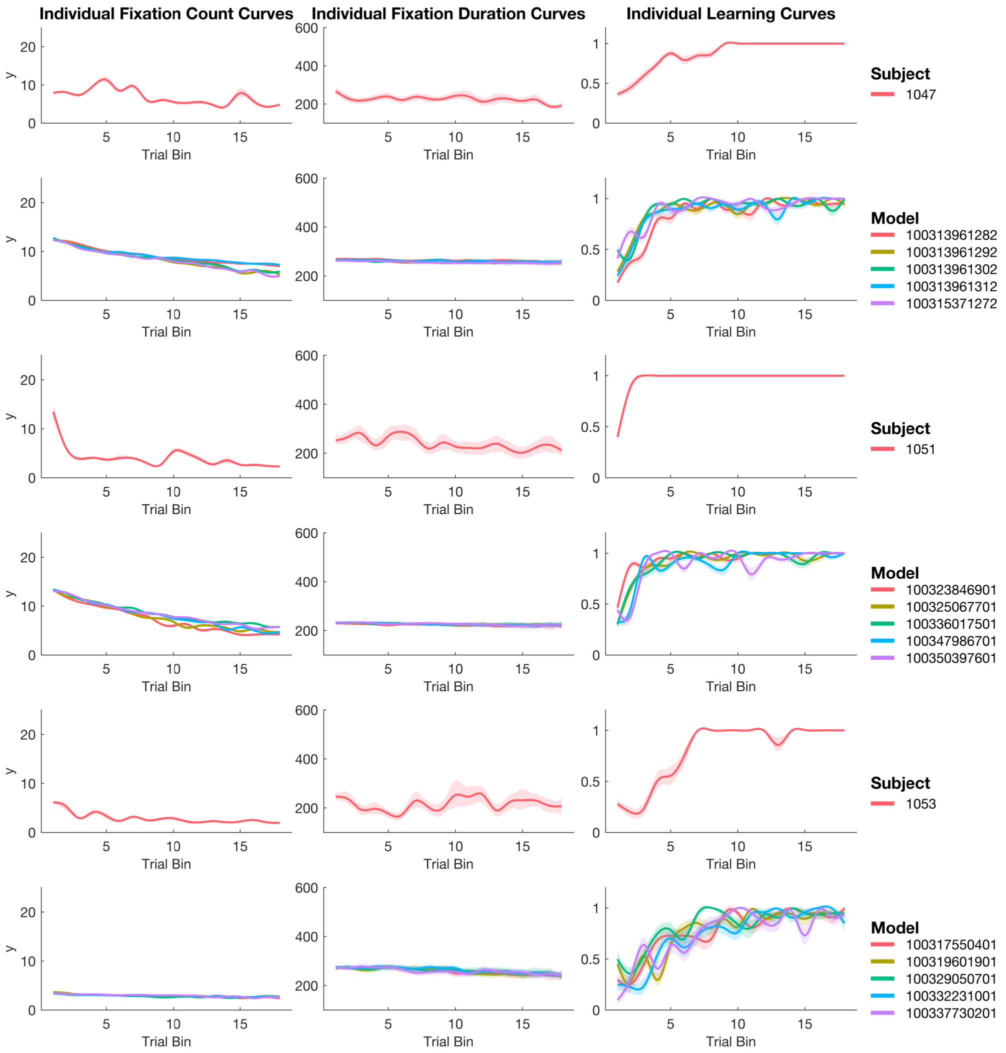

Figure 26. Subjects: 1047, 1051, 1053. Subject fixation counts, fixation durations, and learning curves are contrasted with their best fitting sample of models in the next row.

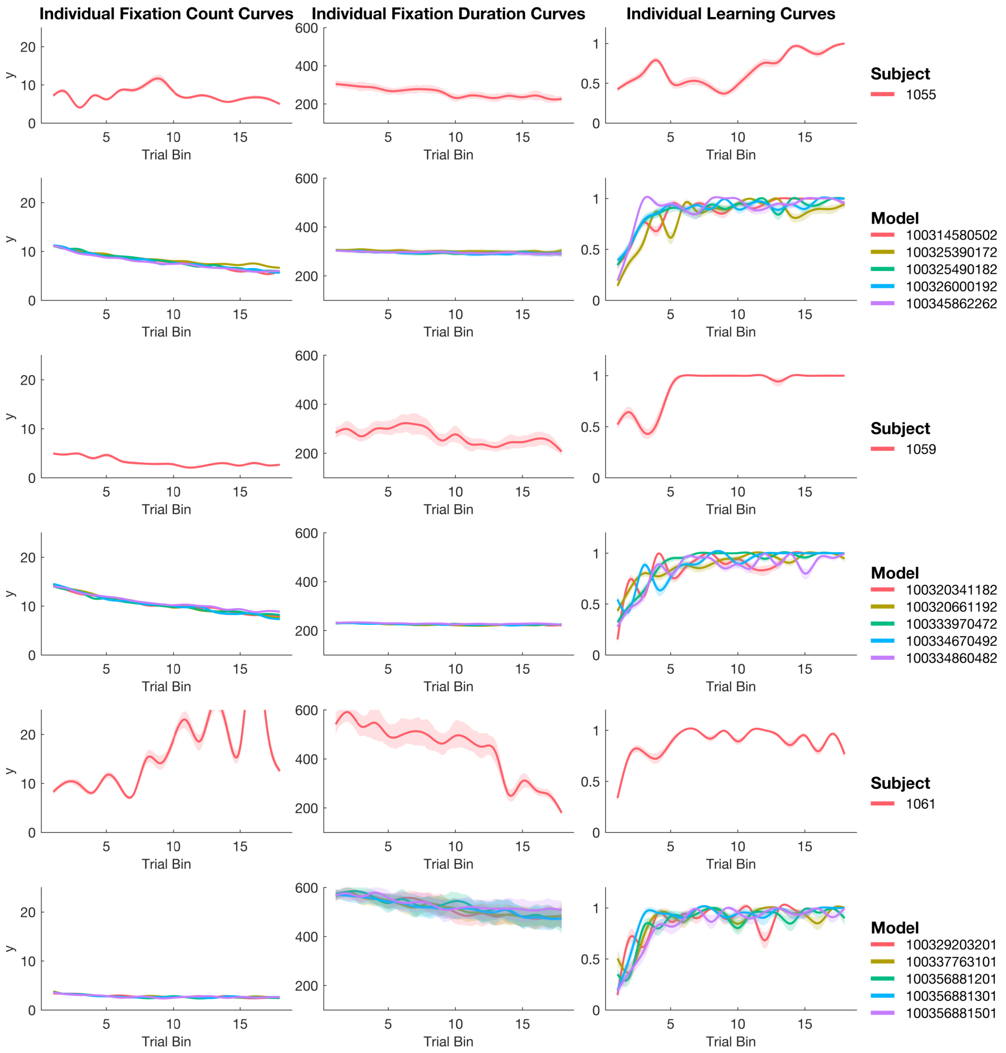

Figure 27. Subjects: 1055, 1059, 1061. Subject fixation counts, fixation durations, and learning curves are contrasted with their best fitting sample of models in the next row.

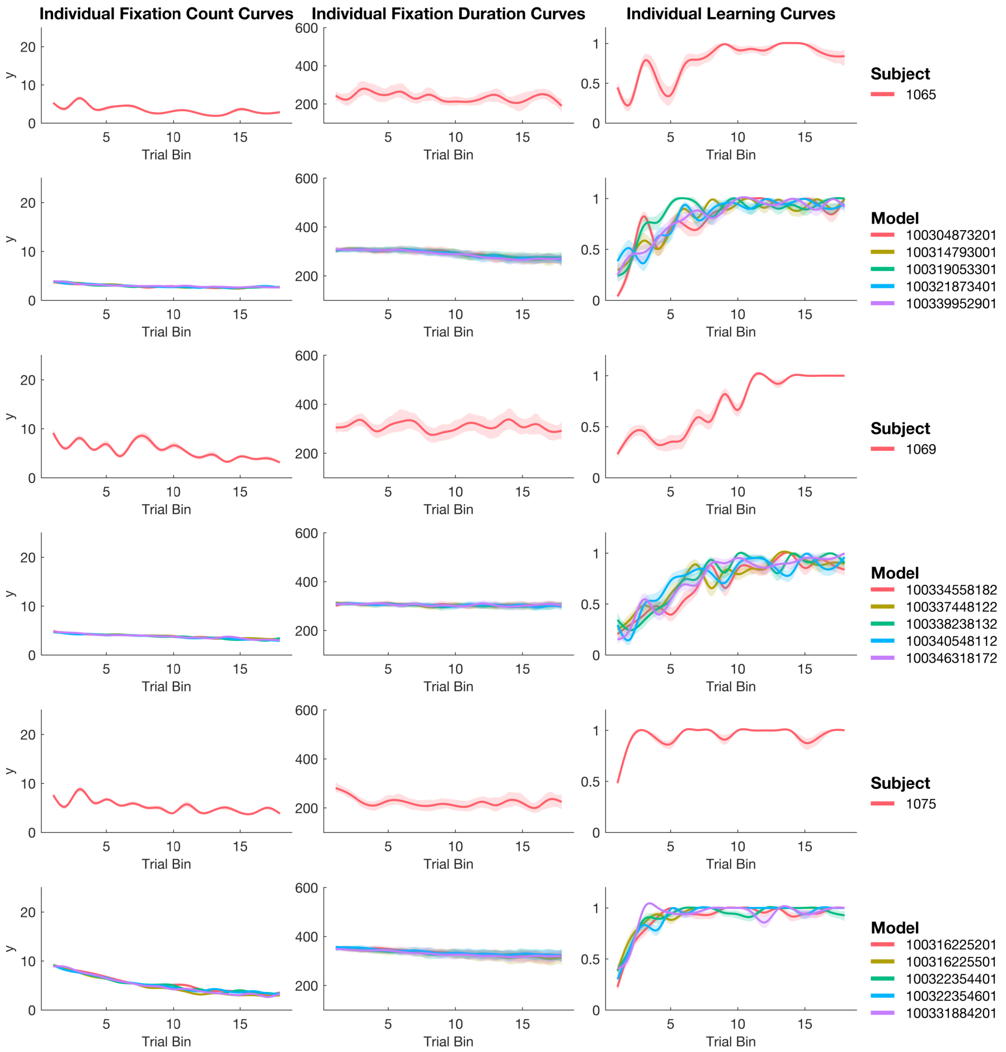

Figure 28. Subjects: 1065, 1069, 1075. Subject fixation counts, fixation durations, and learning curves are contrasted with their best fitting sample of models in the next row.

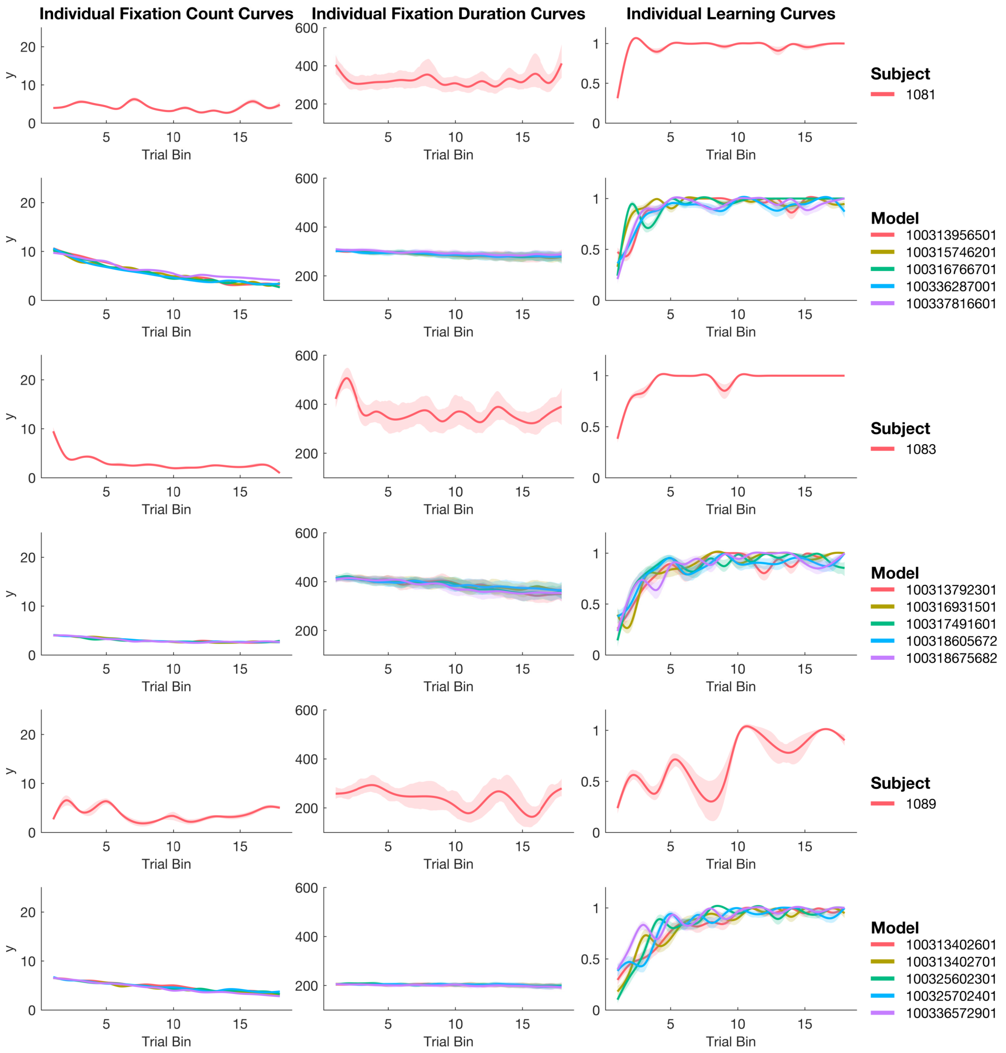

Figure 29. Subjects: 1081, 1083, 1089. Subject fixation counts, fixation durations, and learning curves are contrasted with their best fitting sample of models in the next row.

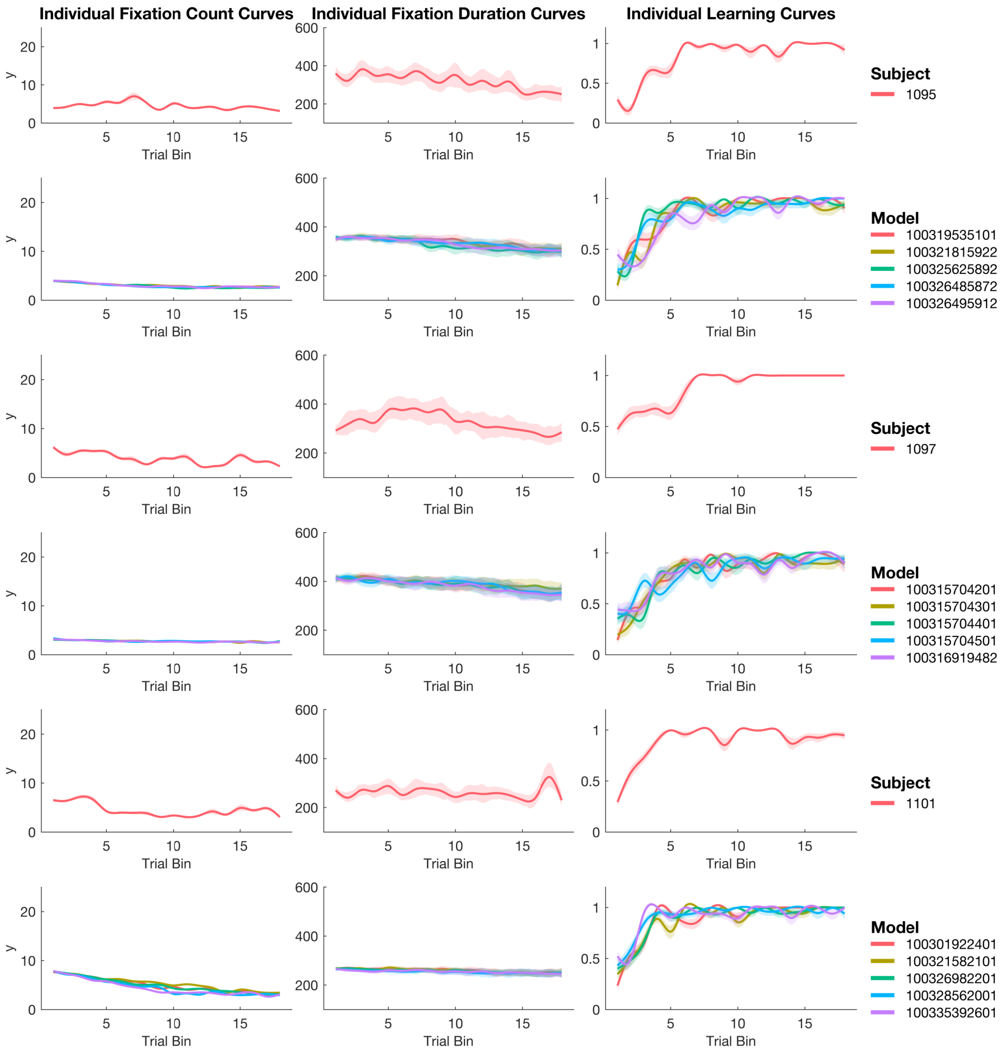

Figure 30. Subjects: 1095, 1097, 1101. Subject fixation counts, fixation durations, and learning curves are contrasted with their best fitting sample of models in the next row.

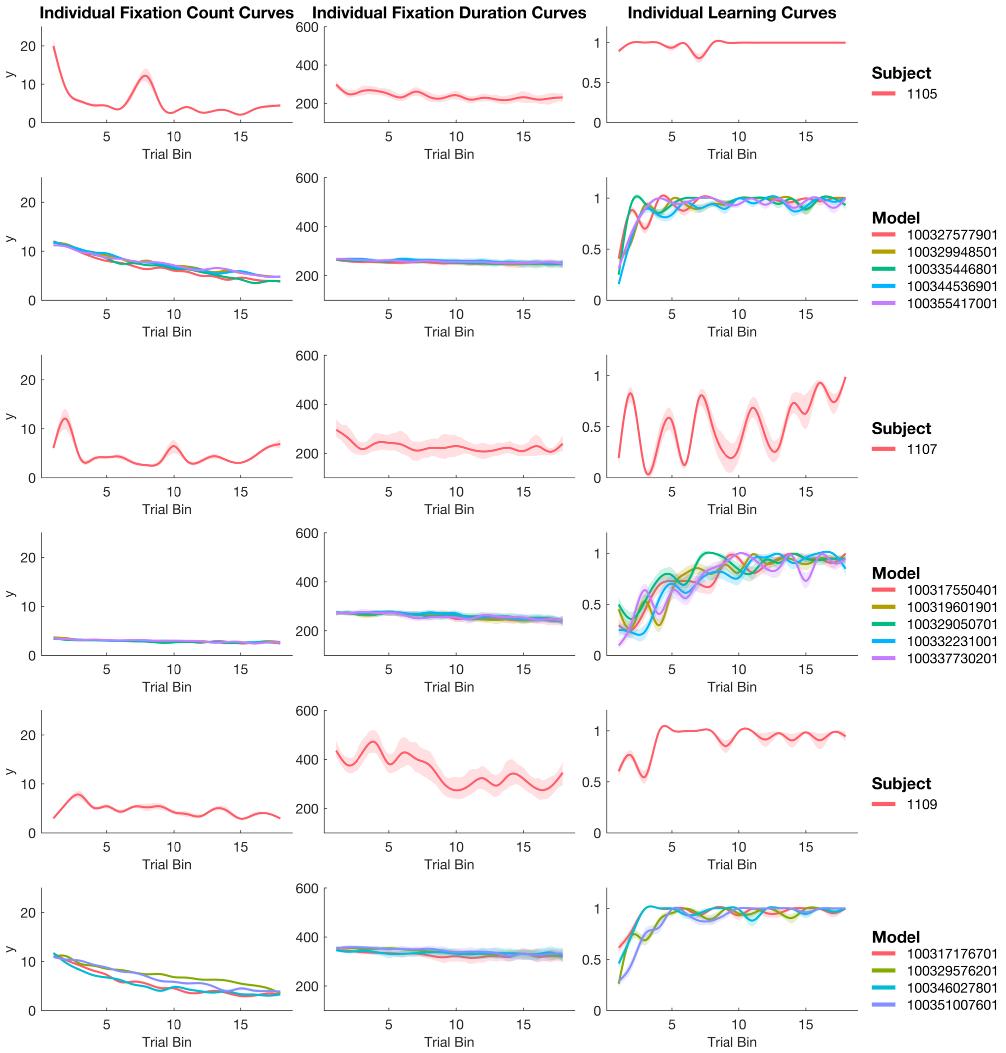

Figure 31. Subjects: 1105, 1107, 1109. Subject fixation counts, fixation durations, and learning curves are contrasted with their best fitting sample of models in the next row.

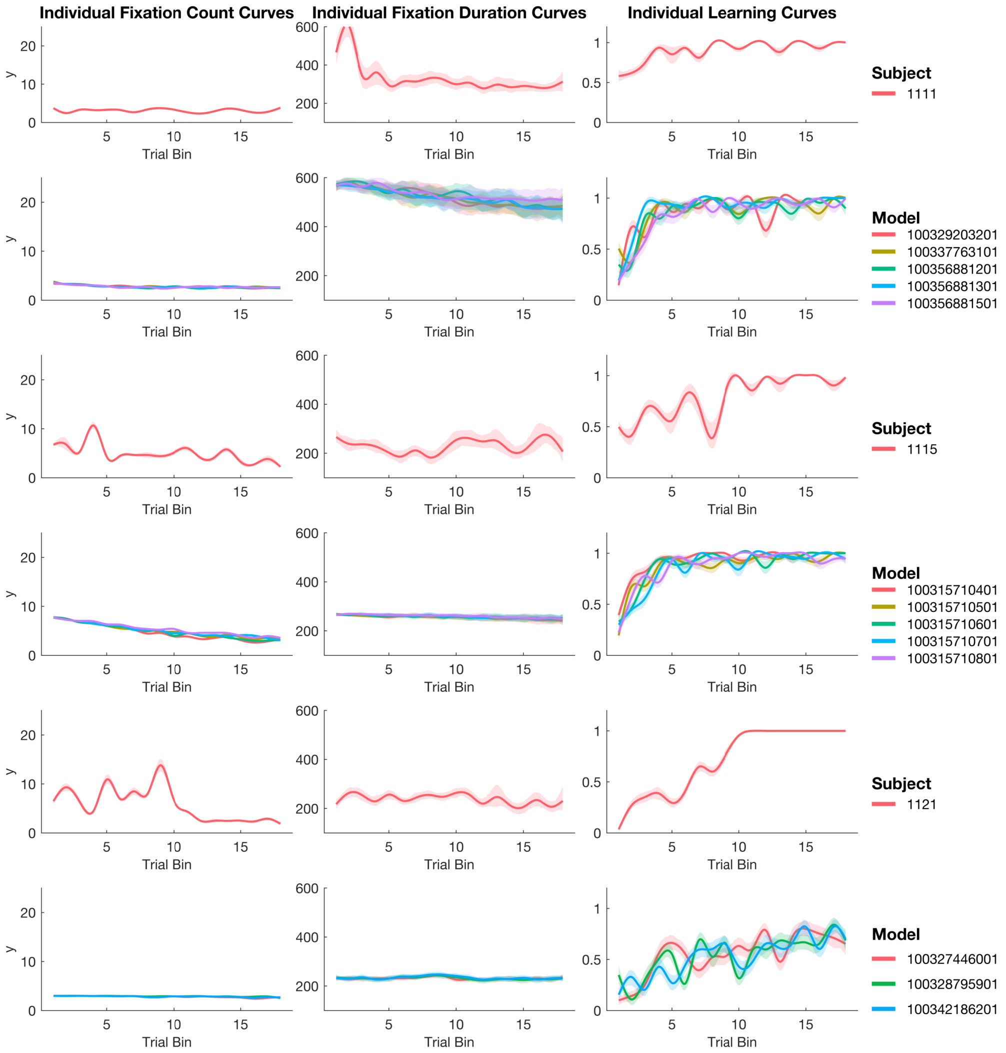

Figure 32. Subjects: 1111, 1115, 1121. Subject fixation counts, fixation durations, and learning curves are contrasted with their best fitting sample of models in the next row.

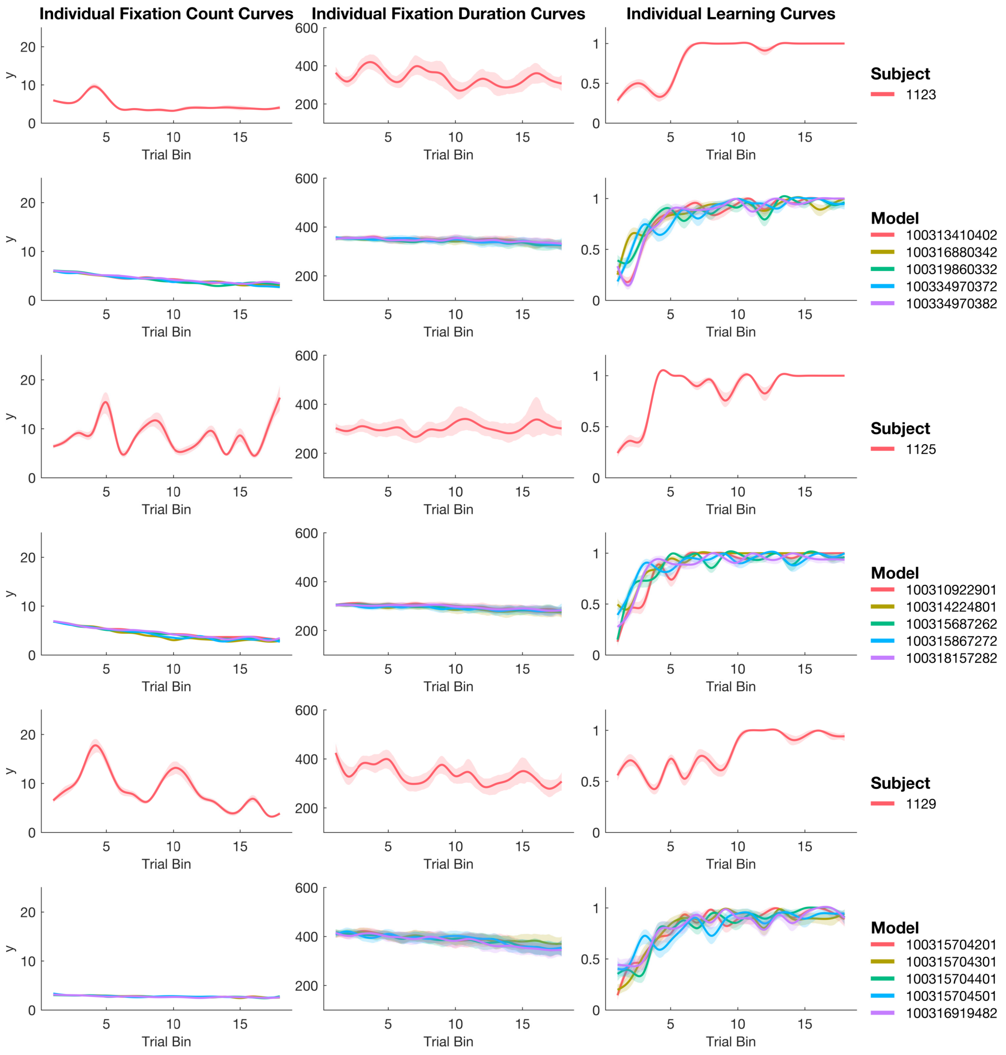

Figure 33. Subjects: 1123, 1125, 1129. Subject fixation counts, fixation durations, and learning curves are contrasted with their best fitting sample of models in the next row.

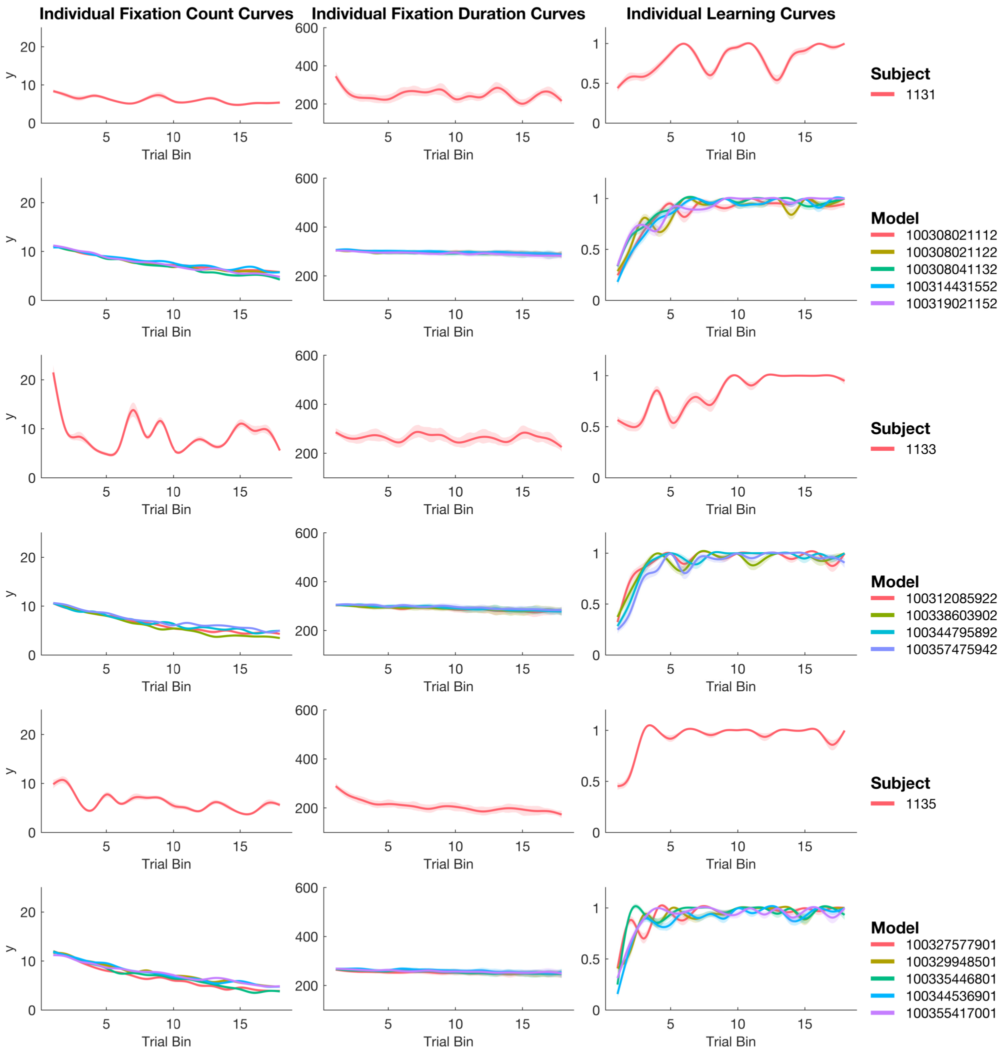

Figure 34. Subjects: 1131, 1133, 1135. Subject fixation counts, fixation durations, and learning curves are contrasted with their best fitting sample of models in the next row.
